# Polyoxygenated cyathane diterpenoids from the mushroom *Cyathus africanus*, and their neurotrophic and anti-neuroinflammatory activities

**DOI:** 10.1038/s41598-018-20472-4

**Published:** 2018-02-01

**Authors:** Jing Wei, Wan-Hui Guo, Chen-Yu Cao, Rong-Wei Kou, Yuan-Zhen Xu, Marcin Górecki, Lorenzo Di Bari, Gennaro Pescitelli, Jin-Ming Gao

**Affiliations:** 1 Shaanxi Key Laboratory of Natural Products & Chemical Biology, Shaanxi Engineering Center of Bioresource Chemistry & Sustainable Utilization, College of Chemistry & Pharmacy, Northwest A&F University, Yangling, 712100 Shaanxi People’s Republic of China; 2 Dipartimento di Chimica e Chimica Industriale, Università di Pisa, via Moruzzi 13, I-56124 Pisa, Italy; 3 College of Biology Pharmacy & Food Engineering, Shangluo University, Shangluo, 726000 Shaanxi People’s Republic of China

## Abstract

In a previous study, we reported ten new polyoxygenated cyathane diterpenoids, neocyathins A–J, and their anti-neuroinflammatory effects from the liquid culture of the medicinal Basidiomycete *Cyathus africanus*. In the present study, eight new highly polyoxygenated cyathane diterpenoids, named neocyathins K–R (**1**–**8**), were isolated from the solid culture of *C. africanus* cultivated on cooked rice, together with three known congeners (**9**–**11**). The structures and the absolute configurations of the new compounds were elucidated through comprehensive NMR and HRESIMS spectroscopic data, electronic circular dichroism (ECD) data, and chemical conversion. Compounds **1** and **2** represent the first reported naturally occurring compounds with 4,9-*seco-*cyathane carbon skeleton incorporating an unprecedented medium-sized 9/7 fused ring system, while the 3,4-*seco-*cyathane derivative (**3**) was isolated from *Cyathus* species for the first time. All compounds were evaluated for their neurotrophic and anti-neuroinflammatory activity. All the isolates at 1–25 μM displayed differential nerve growth factor (NGF)-induced neurite outgrowth-promoting activity in PC-12 cells, while one of the compounds, allocyathin B_2_ (**11**), inhibited NO production in lipopolysaccharide (LPS)-stimulated microglia BV-2 cells. In addition, molecular docking studies showed that compound **11** generated interactions with the inducible nitric oxide synthase (iNOS) protein.

## Introduction

The prevalence of neurodegenerative diseases such as Alzheimer’s disease (AD) will continue to rise steadily. Despite the advancement of current treatments with respect to the past, the management of these diseases remains largely ineffective. Therefore, it is vital to explore novel bioactive natural products to mitigate neurodegenerative disorders. Neurotrophic factors such as nerve growth factor (NGF) have generated much excitement over the past decade due to their therapeutic potential in regulating the proliferation, survival, migration, and differentiation of cells in the nervous system^1^. However, NGF cannot cross the brain blood barrier because it is a high-molecular mass polypeptide and is easily metabolized by peptidases under physiological conditions^2^. To overcome this issue, considerable efforts have been made to find small molecules that have neurotrophic properties and/or that are capable of enhancing the action of endogenous neurotrophic factors. Neuroinflammation, partly due to uncontrolled microglial activation, may contribute to the pathogenesis of neurodegenerative diseases, such as Alzheimer’s disease (AD). Microglial cells are inflammatory cells involved in the regulation of neurodegeneration^3^. Activated microglial cells in the central nervous system (CNS) can produce inflammatory mediators such as nitric oxide (NO); the overproduction of NO in the CNS, resulting from the production of inducible nitric oxide synthase (iNOS), can result in uncontrol-led neuroinflammation^4^. Therefore, promoting counter regulatory mechanisms is essential to avoid inflammation-mediated injury in the CNS^5^, and would thus require therapeutic agents that possess anti-inflammatory action, targeting over-activated microglia cells.

Basidiomycetes are known to produce a broad spectrum of secondary metabolites. A number of cyathane diterpenoids with an unusual 5/6/7 tricyclic skeleton, including their xylosides, were isolated from a variety of higher Basidiomycetes of the genera *Cyathus*, *Hericium*, and *Sarcodon*^6,7^. These diterpenes were demonstrated to display a wide range of biological properties, including anti-inflammatory, antitumor, and antagonism toward the kappa-opioid receptor^7–9^. Several cyathane diterpenoids were found to stimulate the NGF-induced neurite outgrowth activity in PC-12 cells^10–12^, indicating their potential as therapeutic agents to treat neurodegenerative ailment.

**Figure 1:**
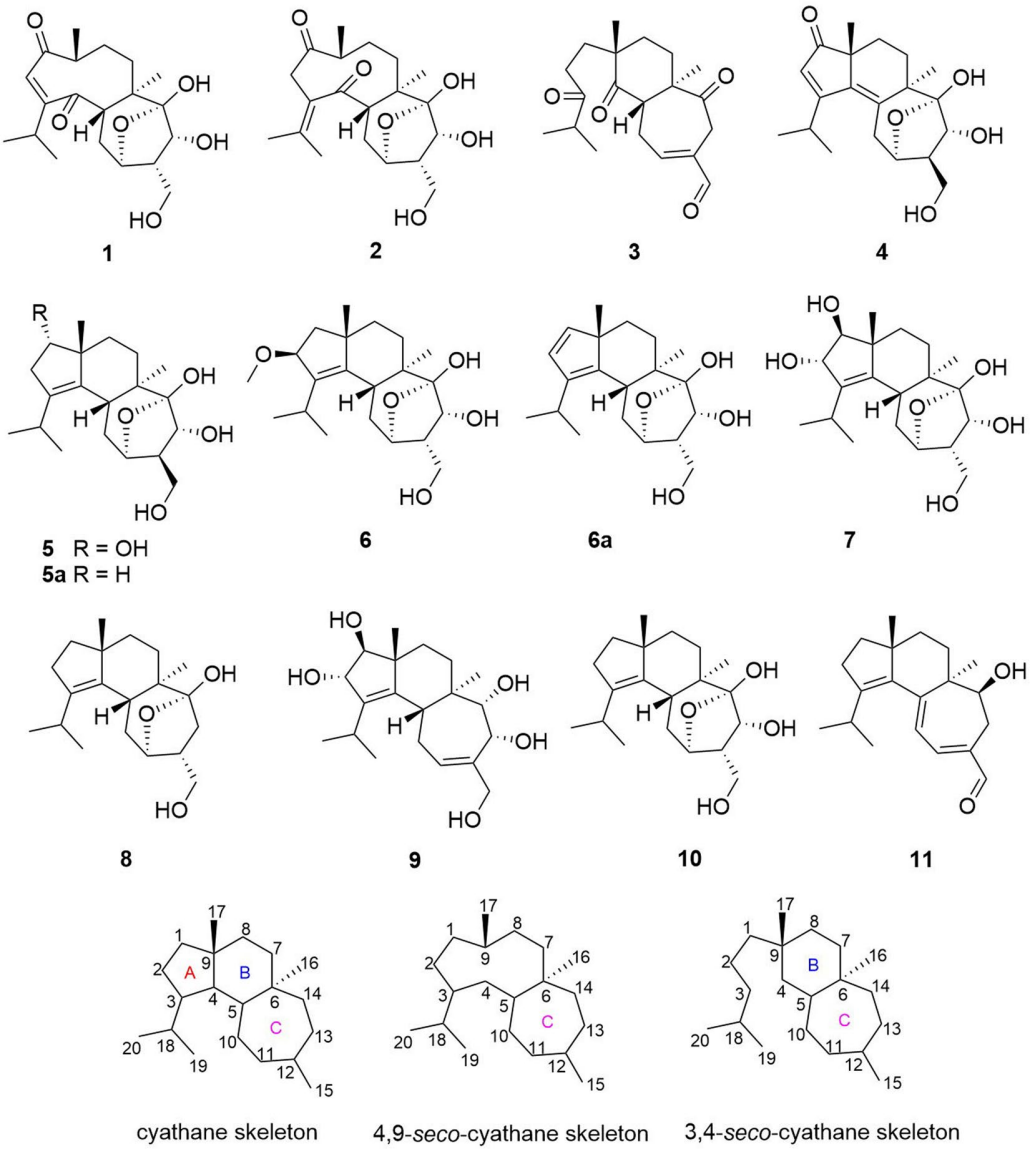
Structures of compounds, cyathane skeleton, and 4,9-*seco*-cyathane skeleton.

The fungi *Cyathus* are a genus in the family of *Nidulariaceae*, collectively known as the bird’s nest fungi. Although generally inedible, *Cyathus* species are well known as prolific producers of bioactive cyathane diterpenoids. We have been actively searching for novel bioactive compounds from basidiomycete fungi^13–17^. To gain access to this potential reservoir of bioactive compounds, the cryptic biosynthetic pathways have to be induced. Our previous phytochemical investigations of the medicinal mushroom *C. africanus* grown in liquid culture led to the discovery of 14 cyathane diterpenoids including ten new ones, neocyathins A–J, and they showed differential effects on the expression of iNOS and cyclooxygenase-2 (COX-2) in LPS-stimulated and Aβ_1–42_-treated BV-2 cells^18^. Many findings have proved that one of these strategies, OSMAC (one strain, many compounds) involving the alteration of cultivation parameters, is an effective strategy to explore the biosynthetic potential of the fungi^19–21^. To further explore its potential in production of biologically active metabolites, the OSMAC strategy was applied to maximize the chemical diversity of this fungus. The fungus *C. africanus* was grown in the solid-substrate medium in static condition, and further detailed chemical investigation led to the isolation of eight new cyathane diterpenoids (Fig. 1), namely, neocyathins K–R (**1**–**8**), along with three known congeners (**9**–**11**), different from compounds isolated from the liquid culture^18^, further demonstrating that using different media may produce different metabolites. In this paper, the isolation, structure elucidation, and biological activities of these compounds from *C. africanus* growing on cooked rice are described.

## Results and Discussion

### Chemistry

The chromatographic separation of the crude *C. africanus* extract using column chromatography on silica gel, Sephadex LH-20, and semipreparative HPLC, led to the isolation of eight new cyathane diterpenoids, namely, neocyathins K–R (**1**–**8**, Fig. 1), along with three known analogues recognized as cyathin V (**9**)^22^, (12 *S*)-11α,14α-epoxy-13α,14β,15-trihydroxycyath-3-ene (**10**)^23^, and allocyathin B_2_ (**11**)^8,24^.

Compound **1** was isolated as a yellow oil. Its molecular formula was determined to be C_20_H_30_O_6_ on the basis of analysis of the NMR and HRESIMS data at *m/z* 389.1933 [M+Na]^+^, indicating six degrees of unsaturation. The IR strong absorptions at 3431 and 1693 cm^−1^ and UV absorption maxima at 230 nm of **1** indicated the presence of hydroxyl and α,β-unsaturated conjugated ketone functionalities. The ^1^H NMR spectrum (Table 1) revealed the presence of four methyls [δ_H_ 0.99 (3 H, d, *J* = 6.4 Hz, H_3_-17), 1.12 (3 H, d, *J* = 6.9 Hz, H_3_-19), 1.15 (3 H, d, *J* = 6.9 Hz, H_3_-20), 1.21 (3 H, s, H_3_-16)], one olefinic proton [δ_H_ 5.90 (1 H, d, *J* = 1.0 Hz, H-2)], two oxymethines [δ_H_ 4.18 (1 H, m, H-11), 4.22 (1 H, d, *J* = 7.8 Hz, H-13)], and two oxymethylene protons [δ_H_ 3.55 (1 H, dd, *J* = 8.9, 11.0 Hz, H-15a), 3.78 (1 H, dd, *J* = 5.7, 11.0 Hz, H-15b)]. The ^13^C NMR data (Table 2) showed 20 carbon resonances ascribed to four methyls (one singlet and three doublets), four methylenes (one oxygenated), seven methines (two oxygenated and one olefinic), and five nonprotonated quaternary carbons (one olefinic, two carbonyl and a ketal carbon). The NMR spectroscopic data of carbons (C-10 to C-15) of **1** were similar to those (ring C) of **10** (Fig. 1), a known cyathane diterpene obtained from *Strobilurus tenacellu*s^23^, later isolated also from *Cyathus gansuensis*^25^.

**Table 1:** ^1^H (500 MHz) NMR Data for Compounds 1–8, and 6a in methanol-*d*_4_.

No.	1	2	3	4	5	6	6a^a^	7	8
1			1.69 m		3.73 m	1.84 d (13.7) 1.56 m	6.27 (d, 5.4)	3.46 d (6.0)	1.69 m 1.44 m
2	5.90 d (1.0)	3.65 d (13.8) 3.27 d (14.2)	2.61 m 2.52 m	6.00 s	2.69 m 2.16 m	4.24 d (6.5)	6.36 (d, 5.4)	4.36 d (6.0)	2.26 m 1.65 m
5	2.90 dd (12.6, 4.5)	2.91 dd (12.6, 4.1)	3.88 m		2.26 m	2.40 dd (12.9, 3.8)	2.40 (dd, 3.5, 13.2)	2.45 dd (12.9, 4.0)	2.49 m
7	1.98 m 1.29 m	1.87 m 1.21 m	2.37 td (13.8, 4.8) 1.49 m	1.80 m 1.66 m	1.55 m	1.61 m 1.47 m	1.19 (dt, 4.5, 13.7); 1.57 (dt, 4.1, 13.7)	1.57 m 1.52 m	1.40 m 1.28 m
8	1.62 m	1.62 m 2 H	1.91 td (13.8, 4.8) 1.76 m	1.73 m 1.54 m	2.12 m 1.15 m	1.64 m 1.44 m	1.38 (m); 1.72 (m)	1.75 m 1.45 m	1.59 2 H m
9	2.98 m	2.71 m							
10	2.11 dt (13.0, 3.6) 1.35 m	2.15 m 1.28 m	2.95 m 2.59 m	2.76 2 H m	2.19 m 1.72 dd (12.7, 3.2)	2.25 m 1.54 m	1.47 (m); 2.26 (dt, 13.8, 4.1)	2.27 td (12.9, 3.6) 1.60 m	2.17 td (12.9, 3.6); 1.55 m
11	4.18 m	4.16 m	6.87 m	4.42 m	4.29 m	4.18 m	4.07 (bs)	4.19 m	4.18 m
12	2.24 m	2.23 m		2.46 m	2.44 m	2.30 m	2.36 (m)	2.29 m	2.04 m
13	4.22 d (7.8)	4.23 (7.8)	3.46 2 H m	3.65 d (5.2)	4.02 d (4.8)	4.42 d (7.7)	4.52 (m)	4.42 d (7.7)	2.54 m 1.13 dd (13.6, 4.6)
15	3.78 dd (11.0, 5.7) 3.55 dd (11.0, 8.9)	3.77 dd (11.0, 5.7) 3.53 dd (11.0, 9.0)	9.36 s	3.88 dd (10.6, 6.5) 3.60 t (10.6)	3.89 dd (10.9, 6.5) 3.63 t (10.9)	3.82 dd (10.9, 5.9) 3.59 dd (10.9, 8.7)	3.65 (m); 3.76 (m)	3.81 dd (10.9, 5.9) 3.59 dd (10.9, 8.7)	3.47 m
16	1.21 s	1.12 s	0.95 s	1.25 s	1.04 s	1.00 s	0.89 (s)	0.99 s	0.97 s
17	0.99 d (6.4)	0.97 d (6.6)	1.30 s	1.20 s	0.99 s	1.20 s	1.02 (s)	1.12 s	1.04 s
18	2.58 m		2.70 m	3.12 m	3.00 m	2.99 m	2.99 (m)	3.02 m	2.97 m
19	1.12 d (6.9)	1.91 s	1.10 d (6.9)	1.27 d (6.8)	1.03 d (6.8)	1.03 d (6.8)	1.04 (d, 6.6)	1.05 d (6.8)	1.01 d (6.8)
20	1.15 d (6.9)	1.98 s	1.10 d (6.9)	1.24 d (6.8)	0.93 d (6.8)	1.06 d (6.8)	1.09 (d, 6.6)	1.11 d (6.8)	0.92 d (6.8)
OC*H*_3_						3.25 s			

^a^at 600 in CD_3_CN.

**Table 2:** ^13^C (125 MHz) NMR Data for Compounds 1–8, and 6a in methanol-*d*_4_.

No.	1	2	3	4	5	6	6a^a^	7	8
1	209.3	214.9	33.2	212.5	78.1	46.0	145.8	81.9	40.9
2	126.0	45.7	36.3	126.7	39.1	86.3	129.1	74.8	29.5
3	164.6	133.3	217.3	182.1	138.5	141.4	144.0	141.7	141.1
4	210.7	210.1	214.7	140.4	135.1	144.4	142.9	143.5	137.6
5	55.0	53.0	48.0	136.9	39.4	38.8	39.2	39.7	39.7
6	45.0	45.6	56.9	47.6	44.3	44.7	39.1	44.1	45.4
7	32.1	31.5	32.1	27.5	29.3	30.2	32.3	29.8	30.5
8	32.4	29.5	34.4	24.7	26.9	38.6	32.9	36.5	38.2
9	43.1	46.7	47.7	49.4	52.8	48.2	53.4	49.8	49.8
10	33.4	31.1	28.6	29.0	28.0	31.9	31.9	32.4	33.2
11	75.8	75.9	153.9	73.7	74.4	76.7	76.4	76.5	77.8
12	50.0	50.3	136.2	56.0	56.0	49.3	48.9	49.2	45.1
13	70.2	70.5	35.2	73.3	71.8	70.1	71.4	70.1	36.0
14	107.9	108.0	210.8	106.3	106.5	107.6	107.9	107.5	109.3
15	61.8	61.9	194.2	61.2	60.7	62.1	62.9	62.2	66.5
16	11.9	12.4	14.9	22.1	13.0	13.0	13.1	13.0	12.5
17	17.7	15.8	23.8	27.0	24.2	26.7	20.9	21.3	24.1
18	34.0	143.9	41.9	30.7	27.3	27.2	26.9	27.2	27.4
19	21.7	23.8	18.6	22.8	22.3	23.2	24.1	23.4	22.6
20	21.5	22.8	18.6	22.7	21.8	23.0	23.6	22.6	21.6
O*C*H_3_						56.1			

^a^at 150 MHzin CD_3_CN.

**Figure 2:**
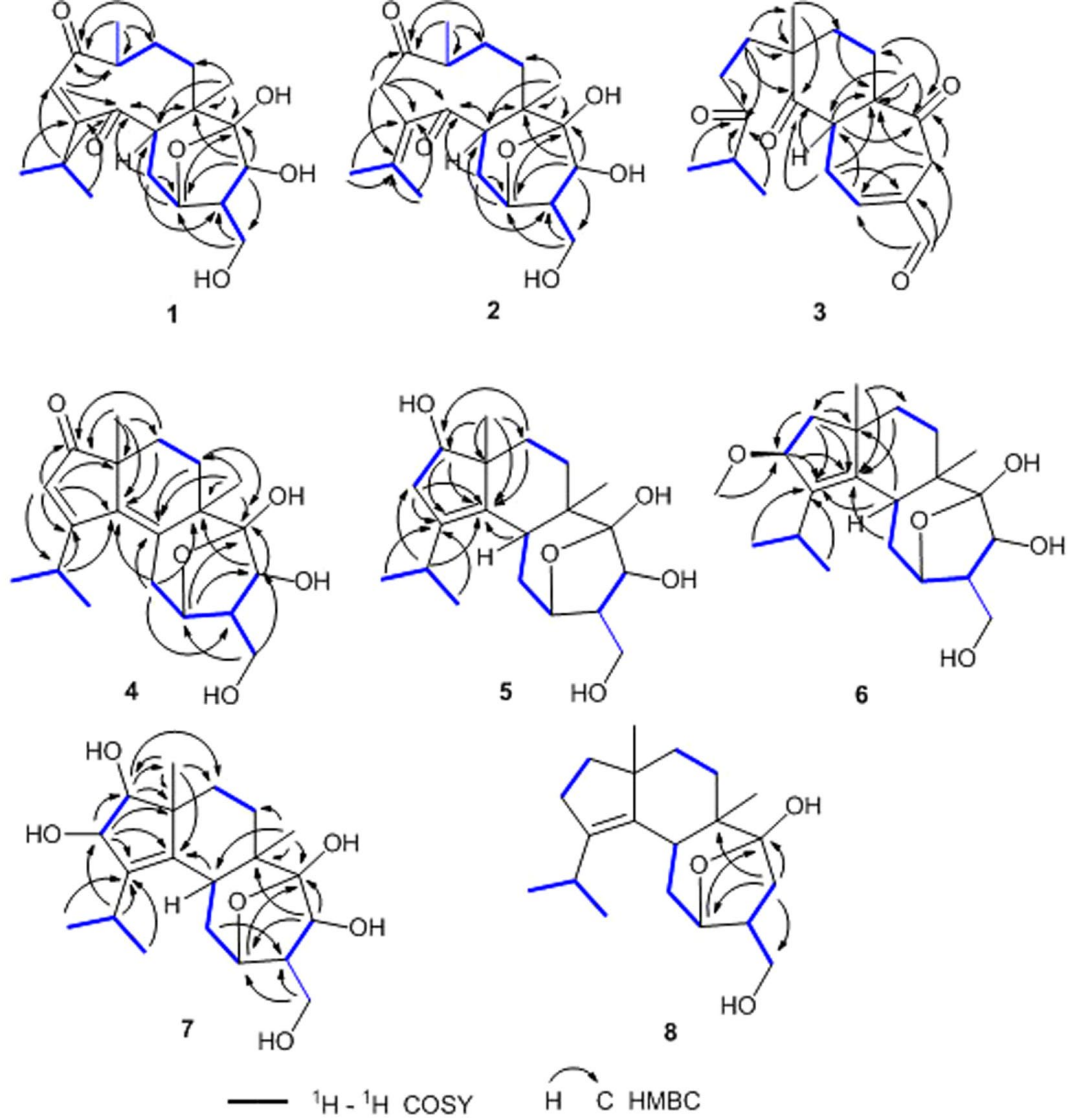
^1^H-^1^H COSY and key HMBC correlations of compounds **1**–**8**.

Analysis of the 1D NMR together with the HMBC and COSY spectra (Fig. 2) allowed the structural establishment of **1**. The ^1^H-^1^H COSY spectrum of **1** displayed the presence of H-5/H_2_-10/H-11, H_2_-15/H-12/H-13, H_2_-7/H_2_-8/H-9/H_3_-17, and H_3_-19/H-18/H_3_-20 coupled spin systems. The planar structure of **1** was determined using HMBC data. HMBC correlations of H_3_-17/C-1 (δ_C_ 209.3), C-8, C-9 (δ_C_ 43.1), H-2/C-3 (δ_C_ 164.6), C-4 (δ_C_ 210.7), C-9, H-16/C-5, C-6, C-7 (δ_C_ 32.1), and H-5 (δ_H_ 2.90)/C-4, C-6 (δ_C_ 45.0) implied the existence of a nine-membered ring with doubly α,β-unsaturated 1,4-diketone moiety. HMBC correlations of H-18/C-2, C-3, C-4, and H-19 and H-20/C-3, C-4 suggested the isopropyl group to be located at C-3. Further HMBC correlations of H-10/C-11 (δ_C_ 75.8), C-12, H-11/C-5, C-14 (δ_C_ 107.9), H-13 (δ_H_ 4.22)/C-6, C-11, C-14, C-15 (δ_C_ 61.8), H-15/C-11, C-12, and H-16/C-14 indicated the presence of a seven-membered ring and an oxygen bridge between C-11 and C-14. In addition, the ^1^H-^1^H COSY cross-peak of H-9/H_3_-17 and the lack of HMBC cross-peak of H_3_-17/C-4 implied a bond cleavage between C-9 and C-4. Thus, the planar structure of **1** was deduced as shown in Fig. 2.

**Figure 3:**
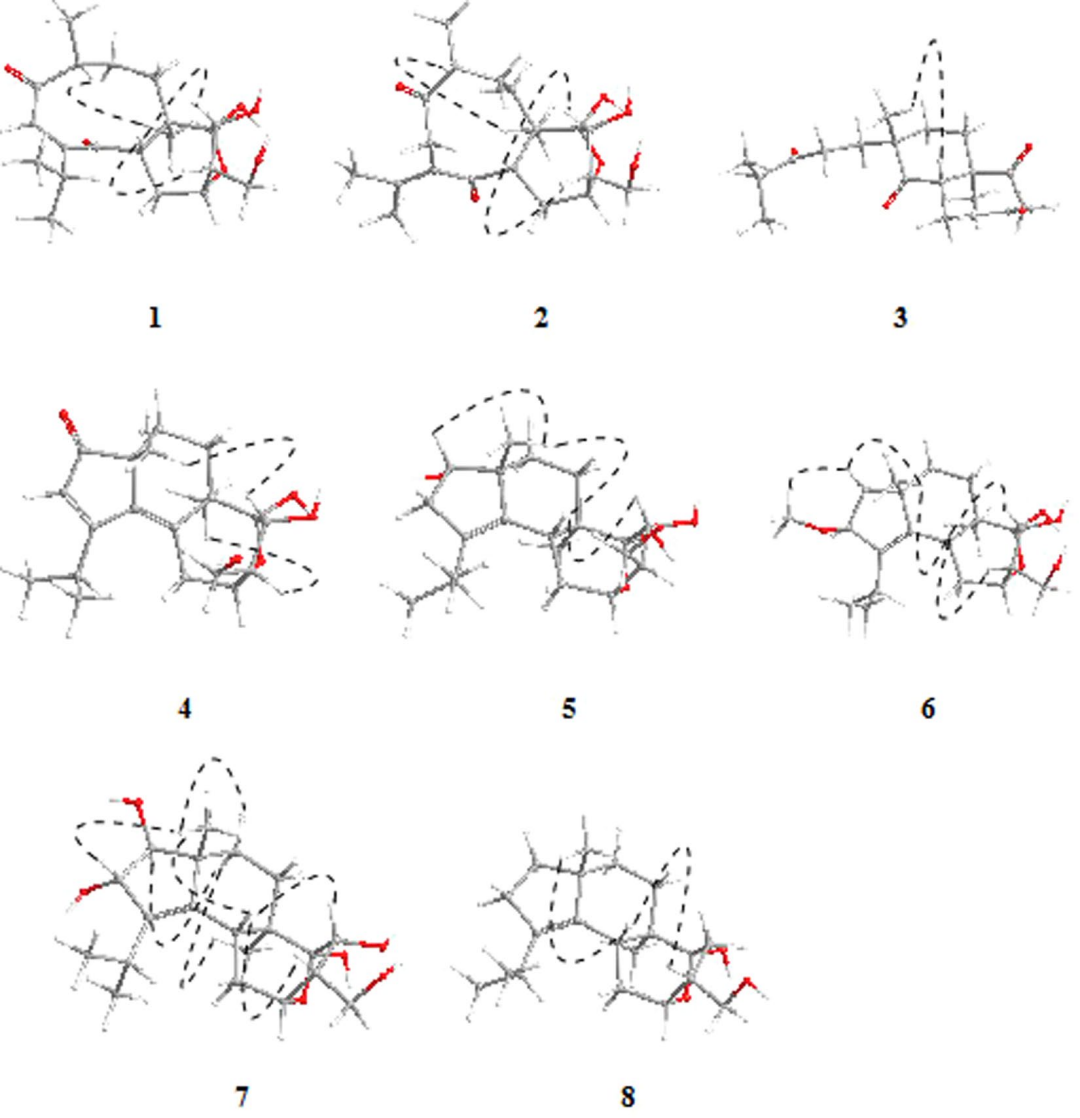
Selected key NOESY correlations of **1–8**. The molecular models of **1–8** in minimal energy were obtained by conformational searches with the DFT/TDDFT calculations.

The relative configuration of **1** was assigned by NOESY data (Fig. 3) with the help of molecular modeling. Observed NOE’s were rationalized using the most stable structures found by the computational procedure described just below. In the NOESY spectrum, the key correlations of H-5/H-12, H-13, and H-9/H_3_-16 indicated the relative configuration of **1** to be (5S*, 6R*, 9R*, 11R*, 12S*, 13R*, 14S*).

**Figure 4:**
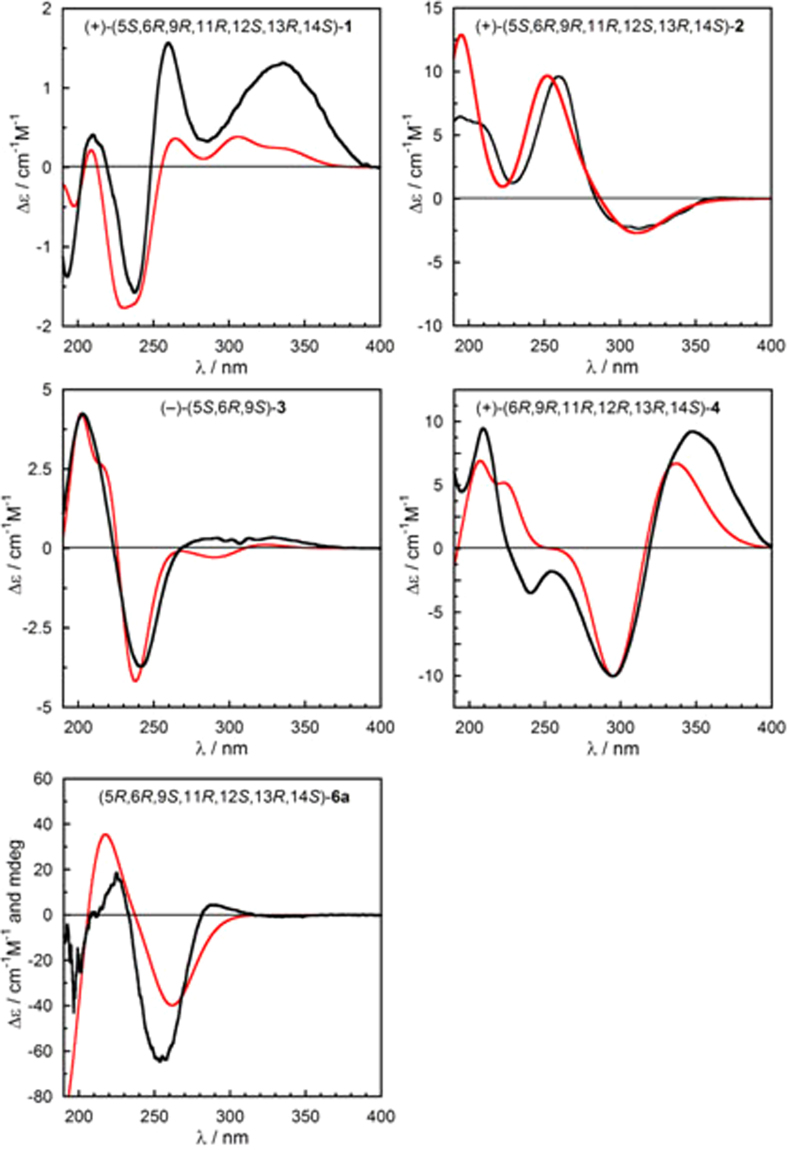
Comparison of experimental (black traces) and calculated ECD spectra (red traces) for **1**–**4**, and **6a**. For computational details, see main text and Supplementary data.

To assign the absolute configuration of all novel compounds, we employed electronic circular dichroism (ECD). The ECD spectrum of **1** measured in acetonitrile (Fig. 4) showed positive Cotton effects (CEs) at 336 nm (Δε + 5.4), 260 nm (Δε + 6.4), 210 nm (Δε + 1.6) and negative CEs at 237 nm (Δε − 6.4), 193 nm (Δε − 5.6). To calculate the ECD spectrum of **1** and other compounds (vide infra), we followed a consolidated computational procedure^26^ detailed in the Computational Section. In brief, after a conformational analysis with molecular mechanics, we run density functional theory (DFT) geometry optimizations including a polarizable continuum solvent model (PCM), and ECD calculations on all relevant energy minima with time-dependent DFT (TDDFT), employing various functionals and PCM^27,28^. Experimental and Boltzmann-averaged calculated ECD spectra were quantitatively compared using a similarity analysis procedure^29^. In the case of compound **1**, 6 conformers were obtained by B3LYP/6–31+G(d,p) geometry optimizations (PCM for acetonitrile). ECD calculations run at B3LYP/TZVP level (PCM for acetonitrile) yielded an average ECD curve in good agreement with the experiment, when the 5*S*, 6*R*, 9*R*, 11*R*, 12*S*, 13*R*, 14*S* configuration was assumed. In fact, the similarity factor (SF)^29^ was 0.8560 for the above configuration and 0.0138 for the enantiomer, thereby implying the absolute configuration of **1** to be (+)-(5*S*, 6*R*, 9*R*, 11*R*, 12*S*, 13*R*, 14*S*)-**1**. Accordingly, the structure of **1** was established as shown, and named neocyathin K.

Compound **2** was purified as a yellow oil and shared the same molecular formula C_20_H_30_O_6_ as **1**, which was deduced from the HRESIMS data. Its IR absorptions at 3420 and 1681 cm^−1^ provided evidence for hydroxy and carbonyl groups. The ^1^H and ^13^C NMR data of **2** resembled those of **1** (Tables 1 and 2). The marked difference between the two compounds was that the isopropyl group[δ_H_/δ_C_:2.58(m)/34.0, 1.12 (d, J = 6.9 Hz)/21.7; 1.15 (d, J = 6.9 Hz)/21.5] at C-3 in **1** was changed into an isopropylidene group[δ_C_143.9, δ_H_/δ_C_: 1.91 (s)/23.8; 1.98 (s)/22.8] containing an exocyclic olefin at Δ^3,18^ in **2**, which caused significant upfield shifts of its C-2 and C-3 resonances (δ_C_ 126.0 vs. 45.7 and 164.6 vs. 133.3, respectively) and downfield shifts of its C-18, C-19, and C-20 resonances (δ_C_ 34.0 vs. 143.9, 21.7 vs. 23.8 and 21.5 vs. 22.8, respectively) compared with those in **1**, suggesting that these two compounds differ by a shifted double bond. In addition, analysis of the NMR data of **2** revealed the presence of an isopropylidene group with resonances corresponding to one tetrasubstituted double bond [δ_C_ 133.3(C-3) and 143.9 (C-18)] and two olefinic methyl singlets [δ_H_1.91 (H_3_-19) and 1.98 (H_3_-20); δ_C_23.8 (C-19) and 22.8 (C-20)]. This deduction was further corroborated by the HMBC cross-peaks (Fig. 2) of H_2_-2 (δ_H_ 3.65, 3.27), H_3_-19, and H_3_-20 with C-3 (δ_C_ 133.3) and C-18 (δ_C_ 143.9). Assignment of the ^1^H and ^13^C NMR data was achieved through HSQC, HMBC, and ^1^H-^1^H COSY experiments.

The relative configuration of **2** was the same as **1**, as assigned by NOESY data (Fig. 3) and molecular modeling. The absolute configuration of **2** was established with the same procedure described above for **1**. Because of the different nature of the chromophores and different folding of the 9-membered ring, the ECD spectra of **1** and **2** were totally different (Fig. 4). Still, the ECD spectrum of **2** was very well reproduced by TDDFT calculations at B3LYP/def2-TZVP level for (5*S*, 6*R*, 9*R*, 11*R*, 12*S*, 13*R*, 14*S*) configuration (SF = 0.9626, SF for enantiomer = 0.0011). Therefore, the absolute configuration of **2** was assigned as (+)-(5*S*, 6*R*, 9*R*, 11*R*, 12*S*, 13*R*, 14*S*)-**2** as shown, and named neocyathin L.

**Figure 5:**
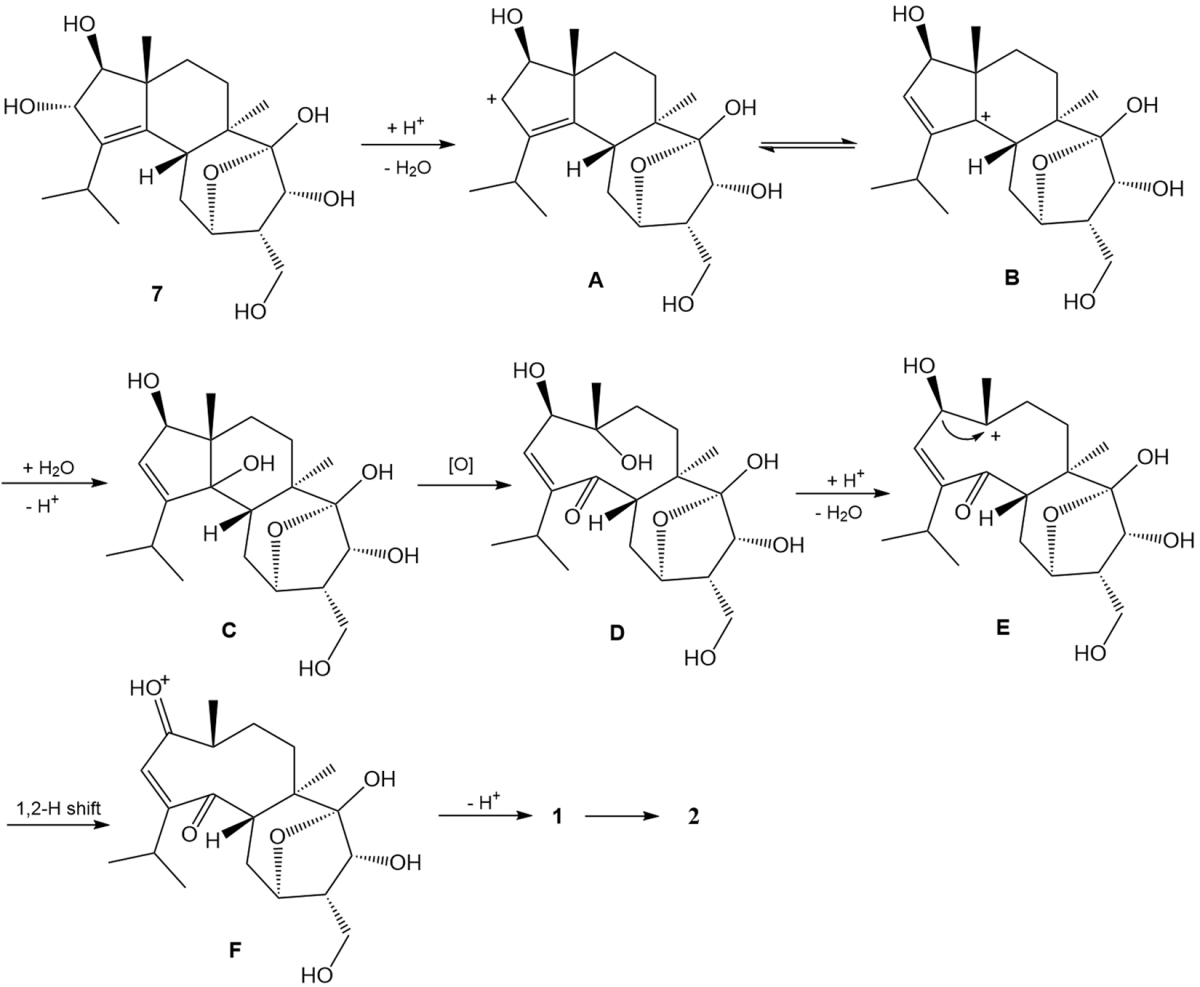
Putative biosynthetic pathway toward **1** and **2**.

To our knowledge, neocyathins K and L (**1**, **2**) represent the first examples of diterpenoids with a rare fused medium-sized 9/7 ring system within carbon skeleton, which aroused our interest in its plausible biogenesis. Analysis of the structure of **1** indicates that it might be formed by oxidative cleavage of compound **7** (Fig. 5). Neocyathin K (**1**) is possibly biosynthesized from compound **7**, a new cyathane diterpene occurring in the same fungus (its structure elucidation is described below). As shown in Fig. 5, the putative biosynthesis of **1** starts from the protonation of 2-OH of **7**, which undergoes elimination of water and double-bond migration to give a carbocation B. This latter reacts with water to generate an intermediate C, which is then subjected to oxidative cleavage, yielding the bicyclic compound D. Subsequently, D is susceptible to elimination, 1,2-H shift, and deprotonation to afford **1**; and upon migration of the Δ^2,3^ double bond, **1** was converted into **2**.

Compound **3** was isolated as a yellow oil. The molecular formula of **3** was determined to be C_20_H_28_O_4_ based on the HRESIMS at m/z 331.1893 [M–H]^−^. The IR strong absorption at 1702 cm^−1^ and UV absorption maximum at 229 nm of **3** indicated the presence of α,β-unsaturated conjugated aldehyde or ketone group. The ^1^H NMR and ^13^C NMR data (Tables 1 and 2) revealed 20 carbon resonances, corresponding to four methyls (two singlets and two doublets), six methylenes, four methines (one olefinic and one aldehyde carbon), and six nonprotonated carbons (one olefinic, three carbonyl). Further analysis of the 1D NMR together with the HMBC and COSY spectra allowed the structural assignment of **3**. In the COSY spectrum, H_2_-1/H_2_-2, H-5/H_2_-10/H-11, H_2_-7/H_2_-8, and H-19/H-18/H-20 substructures were observed. The HMBC correlations of H-17 to C-4 (δ_C_ 214.7), C-8, C-9, H-19 and H-20/C-3 (δ_C_ 217.3), C-18, H-1/C-3, C-4, C-9, C-17, and H-2/C-3 implied the presence of the C_6_ aliphatic side chain, (CH_3_)_2_CHCOCH_2_CH_2_–, attached at C-9 (δ_C_ 47.7), and a bond cleavage between C-3 and C-4 with respect to **1**. HMBC correlations of H-5/C-4, C-6, C-14 (δ_C_ 210.8), H-8/C-6, H-10/C-6, C-12 (δ_C_ 136.2), H-11/C-5, H-13/C-6, C-11 (δ_C_ 153.9), C-14, and H-15 (δ_H_9.36)/C-11, C-12, C-13, suggested the presence of ring B containing one C-4 ketone group and of ring C bearing one α, β-unsaturated aldehyde and one isolated C-14 ketone group. In addition, the chemical shifts of carbons C-10 to C-15 were very similar to that of sarcodonin G, a known diterpene from the fungus *Sarcodon scabrosus*^30^, implying that **3** and sarcodonin G both had the same ring C. The above evidence indicated **3** to be a 3,4-*seco*-cyathin type of diterpenoid. Thus, the planar structure of **3** was elucidated as shown.

The relative configuration of **3** was determined through a NOESY experiment. The NOESY cross-peak of H-5/H_3_-17 indicated that the relative configuration of **3** was (5S*, 6R*, 9S*) (Fig. 3). The absolute configuration of **3** was established with the procedure described above for **1**. Because of the apparent flexibility of the chain attached at C-9, several minima were obtained for **3** by DFT calculations at B3LYP/6–31+G(d,p)/PCM level. However, at least the most stable ones had a very similar calculated ECD spectrum, which is dominated by the relatively rigid and chiral ring system comprising the enal and two carbonyl chromophores. Thus, the experimental ECD spectrum of compound **3** matched very well with the calculated average at CAM-B3LYP/TZVP level for (5 *S*,6 *R*,9 *S*) configuration (Fig. 4; SF = 0.9684, SF for enantiomer = 0.0043). The absolute configuration of **3** could be assigned as (−)-(5 *S*,6 *R*,9 *S*)-**3** as shown, and the compound was named neocyathin M. This is the first report of the isolation of a 3,4-*seco*-cyathane derivative from *Cyathus* species.

The molecular formula of **4** was determined to be C_20_H_28_O_5_ based on the HRESIMS at *m/z* 371.1831 [M+Na]^+^, indicating seven degrees of unsaturation. The NMR spectroscopic data (Tables 1 and 2) of **4** were similar to those of cyathin O^25^. The difference was the replacement of the methylene at C-1 (δ_C_ 53.4) in cyathin O by a carbonyl group (δ_C_ 212.5) in **4**, which was supported by the HMBC correlations of H_3_-17 and H-2/C-1 (Fig. 2). The existence of a conjugated diene at C-2 (δ_C_ 126.7), C-3 (δ_C_ 182.1), and C-4 (δ_C_ 140.4) and C-5 (δ_C_ 136.9) in **4** was supported by the HMBC correlations of H_3_-17/C-4, C-9, H-2/C-3, C-4, and H-16/C-5 (Fig. 2). Assignment of the ^1^H and ^13^C NMR data was achieved through HSQC, HMBC, and ^1^H-^1^H COSY experiments. Thus, the planar structure of **4** was established as shown.

The relative configuration of **4** was determined by a NOESY experiment. The NOESY cross-peaks of H-13/H_3_-17 and H-12/H_3_-16 were observed, suggesting the relative configuration of **4** to be 6R*, 9R*, 11R*, 12R*, 13R*, 14S* (Fig. 3). The experimental ECD spectrum of **4** matched well with the calculated spectrum for (6*R*, 9*R*,11*R*, 12*R*, 13*R*, 14*S*)-**4** at CAM-B3LYP/TZVP/PCM//B3LYP-6–31+G(d,p)/PCM level (Fig. 4; SF = 0.8945, SF for enantiomer = 0.0174), obtained as the Boltzmann average of 8 DFT structures. The absolute configuration of **4** was then established to be (+)-(6 *R*, 9 *R*, 11 *R*, 12 *R*, 13 *R*, 14 *S*)-**4** as shown, and named neocyathin N.

The molecular formula of **5** was determined to be C_20_H_32_O_5_ based on the HRESIMS at *m/z* 375.2148 [M+Na]^+^, indicating that **5** contained one more oxygen atom than the previously reported (12 *R*)-11α,14α-epoxy-13α,14β,15-trihydroxy-cyath-3-ene (**5a**)^23^. The NMR spectroscopic data of **5** were similar to those of **5a**. Compound **5** differed from **5a** for the replacement of the methylene at C-1 (δ_C_ 40.0) in **5a** by an oxygenated methine (δ_C_ 78.1) in **5**, suggesting the presence of a hydroxy group at C-1, which was confirmed by the HMBC correlations of H_3_-17/C-1, H-1/C-3 (δ_C_ 138.5), C-4 (δ_C_ 135.1) and the ^1^H-^1^H COSY correlations of H-1/H-2 (Fig. 2). The major differences between the two compounds resided in the chemical shifts of C-2, C-3, C-4, C-8, and C-9. Owing to the γ-effect exerted by the hydroxyl group at C-1, in fact, C-3, C-4, and C-8 in **5** resonated at high fields (138.5 vs 140.0 ppm, 135.1 vs 136.6 ppm, 26.9 vs 37.6 ppm), while C-2 and C-9 (β-effect) were downfield shifted (39.1 vs 29.2 ppm, 52.8 vs 48.3 ppm). Assignment of the ^1^H and ^13^C NMR data was accomplished through HSQC, HMBC, and ^1^H-^1^H COSY experiments. Thus, the planar structure of **5** was established as shown in Fig. 2.

The relative configuration of **5** was determined through a NOESY experiment. The NOESY cross-peaks of H-5/H_3_-17, H-5/H-13, and H-1/H_3_-17 indicated its relative configuration to be 1S*, 5R*, 6R*, 9R*, 11R*, 12R*, 13R*, 14S* (Fig. 3). The 12R* configuration (β position) is supported by the comparison of the chemical shift of C-12 (δ_C_ 56.0 ppm) with those of co-isolated compounds. In fact, compound **4** (beta position) had the same δ_C12_ of 56.0 ppm, while all other analogs with α position had δ_C12_ in the range 48.9–50.3 ppm. The absolute configuration of **5** could not be directly assigned at the present stage; however, we infer it to be the same of related compound **5a** and co-occurrent analogs **1–4** and **6** presently investigated. Thus, the structure of **5** was established as shown, and named neocyathin O.

The molecular formula of **6** was determined to be C_21_H_34_O_5_ based on the HRESIMS at *m/z* 389.2300 [M+Na]^+^. The ^1^H NMR and ^13^C NMR data (Tables 1 and 2) revealed 21 carbon resonances ascribed to five methyls (one oxygenated, two singlets and two doublets), five methylenes (one oxygenated), six methines (three oxygenated), and five nonprotonated carbons (two olefinic and a ketal carbon).

The NMR spectroscopic data of **6** were similar to those of the known compound **10**, except for the presence of a methoxy at C-2 (δ_C_ 86.3), which was supported by the HMBC correlations of H-2 (δ_C_ 4.24) with O*C*H_3_ (δ_C_ 56.1), and of H-1 and OCH_3_ (δ_H_ 3.25) with C-2 (Fig. 2). The major differences between the two compounds resided in the chemical shifts of C-1, C-4, and C-17. Due to the β-effect exerted by the methoxy group at C-2, carbons C-1, C-17, and C-4 in **6** resonated downfield (46.0 vs 40.9 ppm; 26.7 vs 24.1 ppm; 144.4 vs 137.0 ppm). Assignment of the ^1^H and ^13^C NMR data was accomplished through HSQC, HMBC, and ^1^H-^1^H COSY experiments.

The relative configuration of **6** was assigned by the NOESY cross-peaks of H-1b/H_3_-17, H_3_-21. To assign the absolute configuration of **6**, we measured ECD data (in CH_3_CN) and vibrational CD (VCD in CDCl_3_) of **6**; however, both spectra were very weak. Upon re-measuring NMR spectra in CH_3_CN, unexpectedly, a new semi-synthetic compound **6a** was obtained, which was fully characterized by 1D and 2D NMR data (Tables 1 and 2). Compound **6a** was apparently obtained by an unusual elimination of one molecule of MeOH in the presence of little acidic CD_3_CN. Compound **6a** produced by the accidental reaction contained a diene chromophore with moderately intense ECD spectrum (Fig. 4); its absolute configuration could be therefore assigned by ECD calculations, and be used to assign the parent **6** as well.

In comparison with **6** (Tables 1 and 2), compound **6a** lacks the oxygenated methyl group at δ_H_/δ_C_ 3.25/56.1 ppm, the methine at 4.24/86.3 ppm and the methylene group at 1.56–1.84/46.0 ppm. Instead, the signals for an additional double bond are observed at δ_H_/δ_C_ 6.27/145.8 and 6.36/129.1 ppm, with a *J* = 5.4 Hz typical for a 5-membered endocyclic coupling. The unsaturation is confirmed by [M+Na]^+^ at *m/z* 334.2168 (calcd. for C_20_H_30_O_4_, 334.2144), accounting for six degrees of unsaturation, one more than **6**. Moreover, the UV spectrum showed the absorption band for a conjugated diene (255 nm). Compound **6a** is similar to the reported cyathin I^31^ (Table S1, Supplementary data). The relative configuration of **6a** was confirmed by the ROESY cross-peaks of H-12 with H-11 and H-13, and of H-15 with H-11; and by the lack of a cross-peak between H-15 and H-13. To be noticed that **6a** and cyathin I have a similar chemical shift of C-12 (δ_C_ 48.9 vs 49.5 ppm) despite a different configuration (α-CH_2_OH *vs* β-CH_2_OH). This means that the chemical shift cannot be taken as a proof of the configuration at C-12, as assumed before^23^. To assign the absolute configuration of **6a**, we calculated ECD spectra at CAM-B3LYP/def2-TZVP/PCM level on five conformers optimized at ωB97X-D/6–311 G+(d,p) level. The average calculated spectrum for (5*R*, 6*R*, 9*S*, 11*R*, 12*S*, 13*R*, 14*S*) configuration is in good agreement with the experimental one (Fig. 4; SF = 0.8452; SF for enantiomer = 0.0120). Thus, the absolute configuration of the parent compound **6** is assigned as (+)-(2*S*, 5*R*, 6*R*, 9*S*, 11*R*, 12*S*, 13*R*, 14*S*)-**6**, and the compound was named neocyathin P.

The molecular formula of **7** was determined to be C_20_H_32_O_6_ based on the HRESIMS at *m/z* 391.2085 [M+Na]^+^. The NMR spectroscopic data of **7** were similar to those of the known compound **10**, except for the presence of two oxygenated methines at C-1 (δ_C_ 81.9) and C-2 (δ_C_ 74.8), which was supported by the HMBC correlations of H_3_-17/C-1,C-4, H-1/C-9, C-17,H-2/C-1,C-3, C-4, and of H-18/C-2 (Fig. 2). The major differences between the two compounds resided in the chemical shifts of C-4, C-8, and C-17. Due to the γ-effect exerted by the hydroxyl group at C-1, C-8, C-17 in **7** resonated at higher fields (36.5 vs 38.1, 21.3 vs 24.1 ppm), while due to the β-effect exerted by the hydroxyl group at C-2, C-4 was downfield shifted (143.5 vs 137.0 ppm). Assignment of the ^1^H and ^13^C NMR data was accomplished through HSQC, HMBC, and ^1^H-^1^H COSY experiments.

The relative configuration of **7** was assigned by the key NOESY cross-peaks of H_3_-17/H-2, H-8b, and H-8a/H-1, H_3_-16 as (1S*, 2S*, 5R*, 6R*, 9R*, 11R*, 12S*, 13R*, 14S*) (Fig. 3). The absolute configurations of compounds **7–9** could not be directly assigned at the present stage; however, based on biogenetic considerations, we assume their configuration to be the same co-occurrent analogs **1–4** and **6**. Thus, the structure of **7** was established as shown, and named neocyathin Q.

The molecular formula of **8** was determined to be C_20_H_32_O_3_ based on the HR-ESIMS at *m/z* 343.2266 [M+Na]^+^. The ^1^HNMR and ^13^C NMR data (Tables 1 and 2) revealed 20 carbon resonances, corresponding to four methyls (two singlets and two doublets), seven methylenes (one oxygenated), four methines (one oxygenated), and five nonprotonated carbons (two olefinic and a ketal carbon). The NMR spectroscopic data of **8** were similar to those of compound **9**, except for the absence of a hydroxyl group at C-13 (δ_C_ 36.0), which was supported by the HMBC correlations of H_2_-13 to C-6, C-11, C-14, C-15 (δ_C_ 66.5), and of H_2_-15 to C-13 (Fig. 2). Assignment of the ^1^H and ^13^C NMR data was accomplished through HSQC, HMBC, ^1^H-^1^H COSY, and NOESY experiments. The relative configuration of **8** was determined as (5R*, 6R*, 9R*, 11R*, 12R*, 14R*) (Fig. 3). Thus, the structure of **8** was established as shown, and named neocyathin R.

The molecular formula of **9** was determined as C_20_H_32_O_5_ based on the HRESIMS. It showed good similarity to the previously reported cyathin V^22^ in the NMR, UV, IR, and specific rotation data. However, we revised the relative configuration of the 14-OH group at C-14 in **9** as α-configured due to the cross-peak between H-5 and H-14 in the NOESY spectrum, and not as β-configured. Thus, the relative configuration of **9** should be 1S*, 2S*, 5R*, 6R*, 9R*, 13R*, 14S*.

### Biological activity

**Figure 6:**
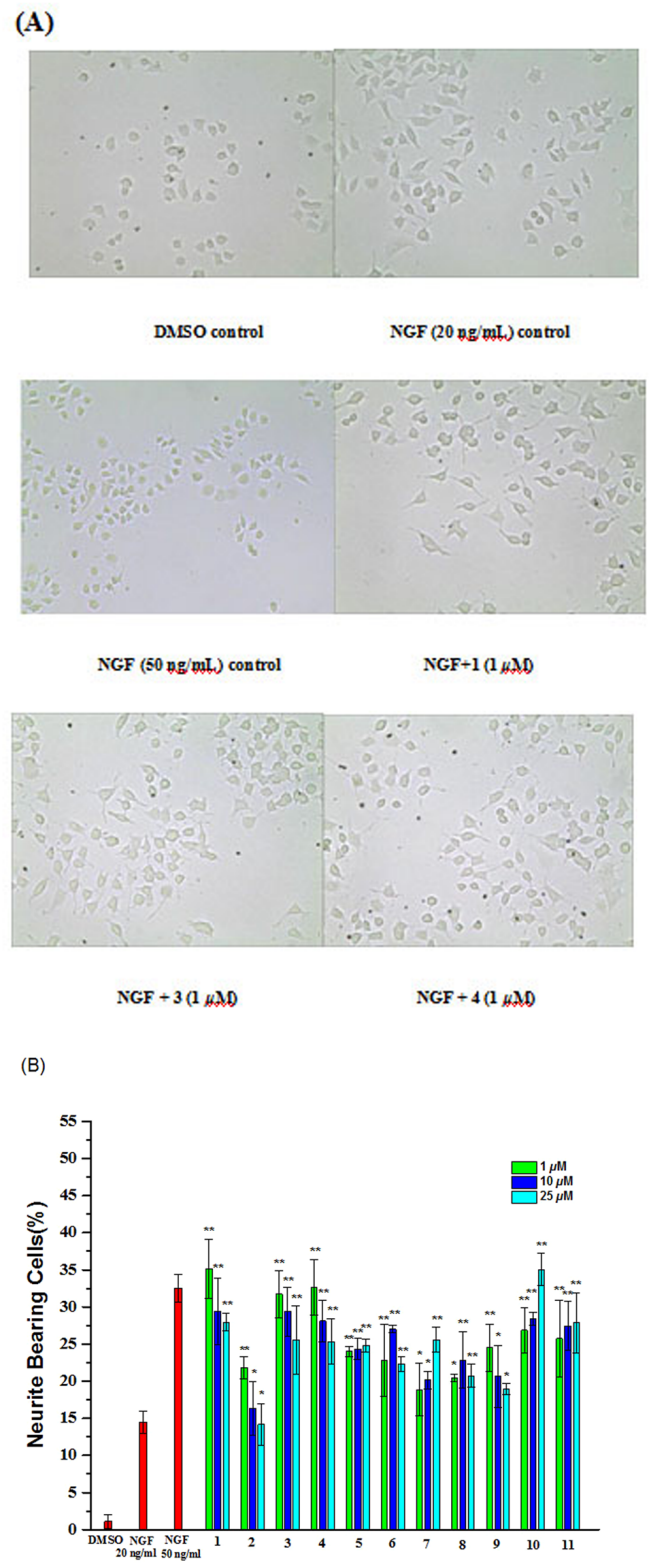
Effects of compounds **1–11** on the NGF-promoted neurite outgrowth in PC-12 cells. PC-12 cells were seeded into poly-L-lysine-coated 24-well plates in normal serum medium for 24 h until adherence, shifted to low serum medium (2% HS and 1% FBS). PC-12 cells were exposed to 0.1% DMSO and 20 ng/mL NGF as a negative control and 50 ng/mL NGF as a positive control, respectively. Cell morphology was observed and photographed as described in Measurement of neurite outgrowth. (**B**) Neurite bearing cells were analyzed as described in Measurement of neurite outgrowth. Data represent the mean ± SD of three independent experiments. ^*^p < 0.05 and ^**^p < 0.01 represent significant differences compared with NGF-treated PC-12 cells (positive control) (ANOVA followed by Dunnett’s posthoc multiple comparisons test).

Compounds **1–11** were evaluated for their neurotrophic properties (neuritogenic activity) using rat pheochromocytoma (PC-12) cells as a model system of neuronal differentiation, and NGF (50 ng/mL) was used as a positive control^12^. To explore the biological activities of these cyathane diterpenoids, we firstly examined the cytotoxicity of the isolated compounds **1–10** in PC-12 cells by the 3-(4,5-dimethylthiazol-2-yl)-2,5-diphenyltetrazolium bromide (MTT) method. As shown in Fig. S53 (Supporting Information), all test compounds at the concentrations of 10 μM for 48 h did not show any toxicity. The cells were incubated with compounds **1**–**11** at concentrations of 1, 10, and 25 μM in the absence and presence of NGF, and the results are listed in Fig. 5. Compounds **1–11** in combination with NGF (20 ng/mL) were found to exert a significant increase in neurite-bearing cells compared to NGF-treated PC-12 cells (Fig. 6) at concentrations ranging from 1 to 25 μM, while none of them was active without NGF. Among the derivatives tested, at 1 **μ**M, compound **1** was the most potent promoter of neurite outgrowth activity, which was significantly stronger than that of positive control, NGF (50 ng/mL), whereas compounds **3** and **4** (at 1 μM) behaved similarly to 50 ng/mL NGF. To further examine effects of four different concentrations of 0.1, 0.5, 1.0, and 10 **μ**M of **1** on neurite outgrowth-promoting activity in PC-12 cells (Figure S54, Supporting Information), we found that **1** possessed the best activity at 1 **μ**M. In contrast, the test compounds alone had no effect on neurite outgrowth in PC-12 cells in the absence of NGF (20 ng/mL). In addition, at 1–25 μM, in case of two 4,9-*seco*-cyathane-type derivatives (**1** and **2**), the neurite network induced by **1** was more remarkable than that generated by **2**, implying that the former might possess stronger neurotrophic activity than the latter. The results suggest that the double bond at C-2 and C-3 may play an important role in activity. With respective to cyathane-type derivatives (**6**, **8**, and **10**), at all same concentrations, compound **10**, bearing an OH group at C-13, promoted relatively stronger neurite outgrowth potency in comparison with **8**, indicating the OH group is contributable to the activity; while compound **6**, containing an additional OMe group at C-2, displayed weaker activity than **10**, which revealed that the OMe group of **6** is detrimental to this activity. These observations of the structure-activity relationship (SAR) analysis have demonstrated that subtle changes in structures of these 11 compounds appear to have a great influence upon the activity. The findings first shed new light on the potential of the polyoxygenated cyathane diterpenoids from the genus *Cyathus*.

To evaluate the anti-inflammatory activities of all isolated compounds, their effects on NO production in LPS-activated murine microglial BV-2 cells were examined, and quercetin was used as positive control, according to previously reported methods^32^. The MTT cytotoxicity test suggested that all compounds had no significant cytotoxicity on BV-2 cells survival at their effective concentrations. The viability of cells treated by compounds was assessed by an MTT method (results not shown). All compounds affected the cell viability at concentrations greater than 50 μM. As a result, allocyathin B_2_ (**11**) was observed to exhibit significant inhibitory effects on NO production with an IC_50_ value of 19.8 μM (natural product quercetin: IC_50_ = 4.3 μM), whereas the remaining compounds had no effect. This may be due to the presence of the conjugated system in **11** that contributes to inhibition of NO production.

**Figure 7:**
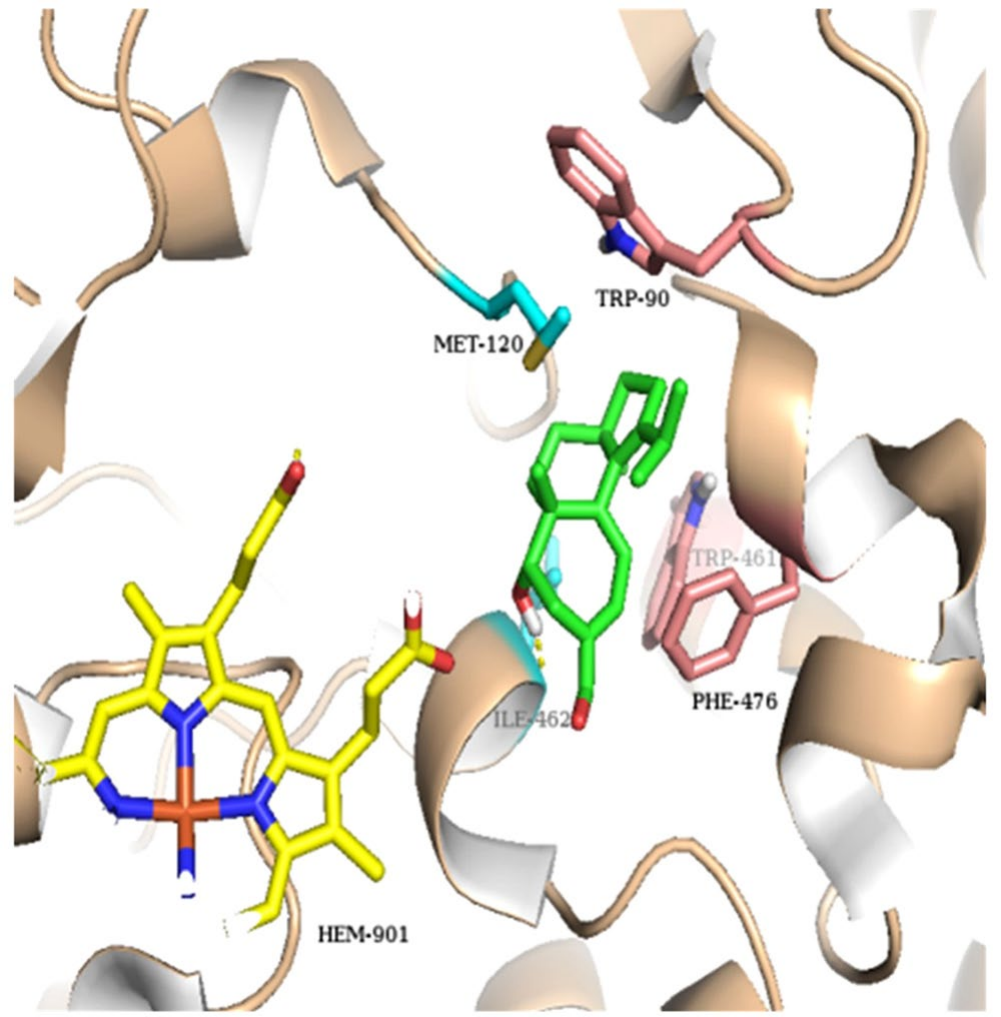
Molecular docking simulations obtained at lowest energy conformation, highlighting potential hydrogen contacts of compound **11** for iNOS. (Colored by atom: carbon of **11** is green; carbon of HEM is yellow; carbon of residues in chain A is cyan; carbon of residues in chain B is pink; nitrogen is blue; oxygen is red; hydrogen is gray; sulfur is orange). Hydrogen-bonding interactions is shown by yellow dashes.

To further elucidate the inhibitory effects of the active compound **11** on iNOS protein, we performed a molecular docking simulation to understand the interaction of **11** with iNOS as previously described^33^. As a result, molecular docking studies revealed that it showed strong interactions with the iNOS protein in the active cavity (Fig. 7). The logarithm of the free binding energy and the binding residues is shown in Table S2 (Supplementary data). Compound **11**, with strong NO inhibitory activity, matched well in the protein-binding pocket by hydrogen bonding with Ile462 (Fig. 7). The results of molecular docking increase our understanding about the possible mechanism of NO inhibitory activities of the compound. Based on these, we postulated that the bioactive diterpenoid might exhibit anti-neuroinflammatory activities by binding to the active cavities of iNOS. Interestingly, **11** displayed two substantial activities as a NO production inhibitor and a neurite outgrowth-inducer.

## Conclusions

In conclusion, we have identified eight new cyathane diterpenoids, neocyathins K–R (**1**–**8)**, from the solid culture of *C. africanus*. Neocyathins K (**1**) and L (**2**) represent the first reported naturally occurring 4,9-*seco*-cyathane compounds possessing a rare 9/7 ring system, and the 3,4-*seco*-cyathane derivative, neocyathin M (**3**), has been isolated from genus *Cyathus* for the first time. Compounds **1**–**11** displayed neurotrophic activity in PC12 cells via NGF-induced neurite outgrowth in PC-12 cells, while only allocyathin B_2_ (**11**) exhibited anti-neuroinflammatory activity via suppression of NO generation in LPS-stimulated BV-2 cells. Molecular docking showed that **11** have strong interactions with iNOS protein by hydrogen bonds targeting residues of the active sites of iNOS, which were in good agreement with NO inhibitory effects. The present results suggested that the bioactive terpenoids may be useful for the development of neurotrophic agents and that allocyathin B_2_ (**11**) may exert a difunctional role in the mitigation of neurodegenerative diseases. Furthermore, the present work and our previous publications have demonstrated that the solid-state fermentation technique is a useful method to trigger the biosynthesis of secondary metabolites in fungi including mushrooms.

## Experimental

### General methods

Optical rotations were recorded on an Autopol III automatic polarimeter (Rudolph Research Analytical). UV and IR spectra were obtained on a Thermo Scientific Evolution-300 UV-visible spectrophotometer and a Bruker Tensor 27 FT-IR spectrometer with KBr pellets. ECD spectra were obtained on a Chirascan spectrometer. NMR spectra were obtained on Bruker Avance 800 MHz and Bruker Avance III 500 spectrometers with tetramethylsilane as an internal standard at room temperature. High-resolution (HR) ESIMS were recorded on a Thermo Fisher Scientific Q-TOF mass spectrometer. Electrospray ionization mass spectrometry ESIMS were recorded on a Thermo Fisher LTQ Fleet instrument spectrometer. Silica gel (300–400 mesh, Qingdao Marine Chemical Ltd., People’s Republic of China) and RP- 18 gel (20–45 μm, Fuji Silysia Chemical Ltd., Japan) were used for column chromatography (CC). Semipreparative HPLC was performed on a Waters 1525EF liquid chromatography system equipped with a Hypersil BDS C18 column (4.6 mm × 250 mm; 10.0 mm × 250 mm) and a Chiral column Chiralpak AD-H (4.6 mm × 250 mm). Fractions were monitored by TLC. Compounds were visualized by heating silica gel plates immersed in 10% H_2_SO_4_ in ethanol.

### Fungal Material

The fungus *C. africanus* was purchased from China General Microbiological Culture Collection Center (CGMCC) with the accession number CGMCC 5.1117. A specimen (No. CA20150823) was deposited at the College of Chemistry & Pharmacy, Northwest A&F University, Shaanxi, China. The fermentation was carried out in one hundred 500 mL Fernbach flasks each containing 80 g of rice and 120 mL of distilled water. All of the flasks were incubated at 28 °C for 90 days.

### Extraction and Isolation

The fermented rice sample was extracted thoroughly with methanol, and the solutions were filtered and evaporated under reduced pressure. The methanol extract of fermented rice was diluted with water to 5 L, then extracted with ethyl acetate (5 L × 3). The EtOAc layer was concentrated under reduced pressure to give a crude extract (43.0 g), which was applied to a RP-18 column eluting with a gradient of MeOH–H_2_O (10–100%) to obtain five fractions, A–E. Fraction E was further fractionated by silica gel CC eluting with a gradient of CHCl_3_–MeOH (50:1–10:1) to yield five fractions (E1-E5). Fraction E2 was subjected to Sephadex LH-20 (MeOH) and further purified by chiral semipreparative HPLC (20%, hexane–isopropanol, 1 mL/min) to furnish **3** (t_*R*_ = 10.0 min, 4.8 mg). Fraction E3 was subjected to Sephadex LH-20 (MeOH) and further purified by chiral semipreparative HPLC (10%, hexane–isopropanol, 1 mL/min) to yield **5** (t_R_ = 6.0 min, 20.0 mg). Fraction D was further purified by silica gel CC eluting with a gradient of CHCl_3_–MeOH (50:1–10:1), to yield five fractions (D1-D5). Fraction D3 was subjected to Sephadex LH-20 (MeOH) and silica gel CC (CHCl_3_–MeOH, 20:1) and further purified by semipreparative HPLC (68%, MeOH-H_2_O, 6 mL/min) to yield **2** (t_R_ = 18.0 min, 1.7 mg). Fraction D4 was subjected to Sephadex LH-20 (MeOH) and further purified by semipreparative HPLC (80%, MeOH–H_2_O, 10 mL/min) to yield **10** (t_R_ = 32.0 min, 8.2 mg). Fraction D5 was subjected to Sephadex LH-20 (MeOH) and silica gel CC (CHCl_3_–MeOH, 50:1) and further purified by chiral semipreparative HPLC (15%, hexane-isopropanol, 1 mL/min) to yield **8** (t_R_ = 20.0 min, 3.9 mg) and **4** (t_R_ = 25.0 min, 5.1 mg). Fraction C was further purified by silica gel CC eluting with a gradient of CHCl_3_-MeOH (50:1–10:1), to yield five fractions (C1-C5). Fraction C2 was subjected to Sephadex LH-20 (MeOH) and silica gel CC (CHCl_3_–MeOH, 55:1) and further purified by semipreparative HPLC (72%, MeOH–H_2_O, 2 mL/min) to yield **9** (t_R_ = 40.0 min, 50.0 mg) and **7** (t_R_ = 50.0 min, 2.2 mg). Fraction B was further purified by silica gel CC eluting with a gradient of CHCl_3_–MeOH (50:1–10:1), to yield five fractions (B1–B5). Fraction B3 was subjected to Sephadex LH-20 (MeOH) and silica gel CC (CHCl_3_−MeOH, 40:1) and further purified by semipreparative HPLC (40%, MeOH–H_2_O, 2 mL/min) to yield **6** (t_R_ = 16.0 min, 1.2 mg) and **1** (t_R_ = 45.0 min, 2.0 mg), and **2** (t_R_ = 47.0 min, 3.0 mg).

#### Neocyathin K (**1**)

Yellow oil; [*α*]_D_^25^ + 69 (*c* 0.1, MeOH); UV (MeOH) *λ*_max_ (log *ε*) 230 (3.33) nm; ECD (*c* 0.9 mg/mL, MeCN) λ_max_ (Δ*ε*) 336 (+5.4), 260 (+6.4), 237 (−6.4), 210 (+1.6), 193 (−5.6) nm; IR (KBr) *ν*_max_ 3431, 2959, 2381, 2310, 1693, 1453, 1380, 1209, 1026 cm^−1^; ^1^H and ^13^C NMR data, see Tables 1 and 2; positive HRESIMS *m/z*389.1933 [M + Na]^+^ (calcd. for C_20_H_30_O_6_Na, 389.1940).

#### Neocyathin L (**2**)

Yellow oil; [*α*]_D_^25^ + 33 (*c* 0.1, MeOH); UV (MeOH) *λ*_max_ (log *ε*) 205 (3.50), 260 (3.40) nm; ECD (*c* 2.0 mg/mL, MeCN) λ_max_ (Δ*ε*) 312 (−21.1), 260 (+86.7), 228 (+11.1), 194 (+58.3) nm; IR (KBr) *ν*_max_ 3420, 2943, 1681, 1448, 1380, 1207, 1162, 1112, 1025 cm^−1^; ^1^H and ^13^C NMR data, see Tables 1 and 2; HRESIMS *m/z* 389.1917 [M + Na]^+^ (calcd. for C_20_H_30_O_6_Na, 389.1940).

#### Neocyathin M (**3**)

Yellow oil; [*α*]_D_^25^ −6 (*c* 0.03, MeOH); UV (MeOH) *λ*_max_ (log *ε*) 229 (3.59) nm; ECD (c 1.0 mg/mL, MeCN) λ_max_ (Δε) 242 (−19.0), 203 (+21.7) nm; IR (KBr) *ν*_max_ 2935, 2311, 1702, 1456, 1379 cm^−1^; ^1^H and ^13^C NMR data, see Tables 1 and 2; HRESIMS *m/z* 331.1893 [M-H]^−^ (calcd. for C_20_H_27_O_4_, 331.1910).

#### Neocyathin N (**4**)

Yellow oil; [*α*]_D_^25^ + 180 (*c* 0.1, MeOH); UV (MeOH) *λ*_max_ (log *ε*) 230 (3.36), 298 (3.40) nm; ECD (c 0.7 mg/mL, MeCN) λ_max_ (Δε) 348 (+31.4), 295 (−34.2), 209 (+32.2) nm; IR (KBr) *ν*_max_ 3386, 2959, 1690, 1374, 1105, 1002 cm^−1^; ^1^H and ^13^C NMR data, see Tables 1 and 2; HRESIMS *m/z* 371.1831 [M + Na]^+^ (calcd. for C_20_H_28_O_5_Na, 371.1834).

#### Neocyathin O(**5**)

Colorless oil; [*α*]_D_^25^+ 2 (*c* 0.1, MeOH); UV (MeOH) *λ*_max_ (log *ε*) 211 (2.82) nm; IR (KBr) *ν*_max_ 3408, 2953, 1641, 1455, 1227, 1014 cm^−1^; ^1^H and ^13^C NMR data, see Tables 1 and 2; HRESIMS *m/z* 375.2148 [M + Na]^+^ (calcd. for C_20_H_32_O_5_Na, 375.2147).

#### Neocyathin P(**6**)

White powder; [*α*]_D_^25^ + 4 (*c* 0.2, MeOH); UV (MeOH) *λ*_max_ (log *ε*) 213 (2.54) nm; IR (KBr) *ν*_max_ 3405, 2944, 2383, 1643, 1384, 1089 cm^−1^; ^1^H and ^13^C NMR data, see Tables 1 and 2; HRESIMS *m/z* 389.2300 [M + Na]^+^ (calcd. for C_21_H_34_O_5_Na, 389.2304).

#### Neocyathin Q (**7**)

Yellow oil; [*α*]_D_^25^ −18 (*c* 0.02, MeOH); UV (MeOH) *λ*_max_ (log *ε*) 208 (3.31) nm; IR (KBr) *ν*_max_ 3397, 2937, 2383, 1706, 1384, 1034 cm^−1^; ^1^H and ^13^C NMR data, see Tables 1 and 2; HRESIMS *m/z* 391.2085 [M + Na]^+^ (calcd. for C_20_H_32_O_6_Na, 391.2097).

#### Neocyathin R(**8**)

White powder; [*α*]_D_^25^ −22 (*c* 0.04, MeOH); UV (MeOH) *λ*_max_ (log *ε*) 208 (2.99) nm; IR (KBr) *ν*_max_ 3354, 2940, 2379, 1709, 1378, 1041 cm^−1^; ^1^H and ^13^C NMR data, see Tables 1 and 2; HR-ESIMS *m/z* 343.2266 [M + Na]^+^ (calcd. for C_20_H_32_O_3_Na, 343.2249).

#### Compound (**9**)

White powder; [*α*]_D_^25^ −79 (*c* 0.1, MeOH); UV (MeOH) *λ*_max_ (log *ε*) 213 (2.90) nm; IR (KBr) *ν*_max_ 3395, 2960, 2928, 1704, 1391, 1062 cm^−1^; ^1^H and ^13^C NMR data, see Tables 1 and 2; HR-ESIMS *m/z* 375.2153 [M + Na]^+^ (calcd. for C_20_H_32_O_5_Na, 375.2147).

#### Compound 6a

Yellowish solid; UV (CD_3_CN) λ_max_ 255.4 nm; ECD (approx. *c* 1.0 mg/mL, MeCN) λ_max_ (ellipticity, mdeg) nm 253 (−63.1), 225 (+16.5); IR (CD_3_CN) ν_max_ 1630, 1465, 1450, 1381, 1220, 1085, 1029, 1012, 918 cm^−1^; ^1^H NMR and ^13^C NMR, see Tables 1 and 2; HRESIMS *m/z* 334.2168 [M + Na]^+^ (calcd. for C_20_H_30_O_4_, 334.2144).

#### Compound (**9**)**:** Cyafricanin **9**

White powder; [*α*]_D_^25^ −79 (*c* 0.1, MeOH); UV (MeOH) *λ*_max_ (log *ε*) 213 (2.90) nm; IR (KBr) *ν*_max_3395, 2960, 2928, 1704, 1391, 1062 cm^−1^; ^1^H and ^13^C NMR data, see Tables 1 and 2; HR-ESIMS *m/z* 375.2153 [M + Na]^+^ (calcd. for C_20_H_32_O_5_Na,375.2147).

#### (12 S)-11α,14α-epoxy-13α,14β,15-trihydroxycyath-3-ene (**10**)

White crystalline powder^23^; ESIMS (positive) *m/z* 359.32 [M + Na]^+^, for ^1^H- and ^13^C- NMR data see Table S1.

#### Allocyathin B_2_ (**11**)

Yellow oil^8,24^; HR-ESIMS (negative) *m/z* 299.2010 [M-H]^−^, for ^1^H- and ^13^C- NMR data see Table S1.

### Computational Section

Conformational searches were run with Conflex v6.7 (CONFLEX Co., Tokyo, 2000) and Spartan’16 (Wave function, Inc., Irvine CA, 2016), with standard parameters and convergence criteria. TDDFT calculations were run with Gaussian’09 (Rev. D.01, Gaussian, Inc., Wallingford CT, 2013), with default grids and convergence criteria^34^. Conformational searches were run with the algorithms implemented in Conflex and Spartan using Merck molecular force field (MMFF94s). All structures thus obtained were optimized with DFT method using B3LYP or ωB97X-D functionals and 6–31+G(d,p) basis sets including the CPCM or IEF-PCM solvent models. Frequencies were calculated at the same level of theory to check the nature of optimized structures. All conformers with populations greater than 1% at 300 K and without imaginary frequencies were considered for ECD calculations. TDDFT calculations were run with several functionals (B3LYP, CAM-B3LYP, ω(B3LYP)) and TZVP or def2-TZVP basis sets, including CPCM or IEF-PCM solvent models for acetonitrile. ECD spectra were generated using the program SpecDis (v. 1.70, T. Bruhn, A. Schaumlöffel, Y. Hemberger, G. Pescitelli, Berlin, Germany, 2017, https://specdis-software.jimdo.com/). For each compound, all conformers with Boltzmann population >1% at 300 K were included in the calculations. The ECD curves were plotted using by applying a Gaussian band shape with 0.25–0.3 eV exponential half-width and UV corrections of 5–10 nm.

### Bioassay Methods

#### Measurement of Neurite Outgrowth in PC-12 Cells

Cell Culture: The rat adrenal pheochromocytoma cell line, PC-12, were obtained from China Center for Type Culture Collection (CCTCC). PC-12 cells were maintained in nutrient mixture F-12 (Ham) medium supplemented with 10% inactivated horse serum (HS), 5% inactivated fetal bovine serum (FBS), 100 U/mL penicillin G, 100 μg/mL streptomycin, and 2.5 g/L sodium bicarbonate at 37 °C in humidified air containing 5% CO_2_. The purity of the isolated compounds is over 95% by NMR.

Morphological analysis and quantification of neurite bearing cells were performed using phase-contrast microscope as described previously^12^. PC-12 cells were seeded on poly-L-lysine-coated 24-well plates at a density of 2 × 10^4^ cells/mL in normal serum medium for 24 h. The F-12 medium containing low serum (1% HS and 0.5% FBS) was replaced prior to exposure to vehicle (0.1% DMSO) or indicated reagents. The cells were treated with tested compounds at various concentrations ranging from 10 μM to 40 μM with 20 ng/mL of NGF. Cells without treatment served as a negative control. Cells treated with 20 ng/mL of NGF served as a positive control. One concentration experiment was repeated in three wells. After an additional 48 h of incubation, neurite outgrowth of PC-12 cells was observed under an inverted microscope using phase-contrast objectives and photographed by the digital camera. Five images were selected randomly under a microscope for each well. At least 100 cells in each of five randomly separated fields were scored and the proportion of cells with neuritis greater than or equal to the length of one cell body were positive for neurite outgrowth, and expressed as a percentage of the total cell number in five fields. Experiments were repeated at least three times and data are expressed as mean ± SD (**p < 0.01)^12^.

#### NO Production Assays

BV-2 cells were from Peking Union Medical College, Cell Bank (Beijing, China) and maintained in DMEM medium supplemented with 10% fetal bovine serum, penicillin (100 U/mL), and streptomycin (100 μg/mL) in a humidified incubator containing 95% air and 5% CO_2_ at 37 °C. In all experiments, cells were treated with LPS (1 μg/mL) with or without the indicated concentrations of compounds for 24 h. The productions of NO were determined by detecting cell culture supernatants for nitrite, a major stable product of NO, by Griess reagent^32^. Briefly, the medium from treated cells cultured in 96-well plates was removed and placed into a 96-well plate (50 μL). Then cell culture supernatants were reacted with 100 μL of Griess reagent (1% sulfanilamide/0.1% naphthylethylene diaminedihydrochloride/2% phosphoric acid) for 10 min at room temperature. The optical density was measured at 540 nm using a micro plate reader. Sodium nitrite was used as a standard curve in the assay.

### Molecular Docking Studies

Molecular docking simulations were performed using the software Autodock 4.2 Vina along with AutoDock Tools (ADT 1.5.6) using the hybrid Lamarckian Genetic Algorithm (LGA) as our previous studies^33^. The three dimensional (3D) crystal structure of iNOS (PDB code: 3E7G) and COX-2 (PDB code: 1CX2) were obtained from the RCSB Protein Data Bank. The standard 3D structure (PDB format) of compounds **11** and **14** were constructed by using the “SKETCH” option function in SYBYL-X, whose configurations were determined by their NOESY spectra and TDDFT ECD calculations. The cubic grid box of 45 Å size (x, y, z) with a spacing of 0.375 Å and grid maps were built. The docking parameters consisted of setting the population size to 150, the number of evaluations to 25,000,000, the number of generations to 270,000, and the number of top individuals that automatically survive to 20, while the number of docking run was set to 40 with other default values during each docking run. The results of the most favorable free energy of binding were chosen as the resultant complex structures.

## Electronic supplementary material

SUPPLEMENTARY INFORMATION

## References

[1] Fumagalli F, Molteni R, Calabrese F (2008). Neurotrophic Factors in Neurodegenerative Disorders. CNS Drugs.

[2] Calissano P, Matrone C, Amadoro G (2010). Nerve growth factor as a paradigm of neurotrophins related to Alzheimer’s disease. Develop. Neurobiol..

[3] Amor S, Puentes F, Baker D, van der Valk P (2010). Inflammation in neurodegenerative diseases. Immunology.

[4] Block ML, Zecca L, Hong JS (2007). Microglia-mediated neurotoxicity: uncovering the molecular mechanisms. Nat. Rev. Neurosci..

[5] McCarty MF (2006). Down-regulation of microglial activation may represent a practical strategy for combating neurodegenerative disorders. Med. Hypotheses.

[6] Gao JM (2006). New biologically active metabolites from Chinese higher fungi. Curr. Org. Chem..

[7] Tang HY, Yin X, Zhang CC, Jia Q, Gao JM (2015). Structure diversity, synthesis, and biological activity of cyathane diterpenoids in higher fungi. Curr. Med. Chem..

[8] Han J (2013). Anti-inflammatory and cytotoxic cyathane diterpenoids from the medicinal fungus *Cyathus africanus*. Fitoterapia.

[9] He L (2016). Identification of a new cyathane diterpene that induces mitochondrial and autophagy-dependent apoptosis and shows a potent *in vivo* anti-colorectal cancer activity. Eur. J. Med. Chem..

[10] Bai R, Zhang CC, Yin X, Wei J, Gao JM (2015). Striatoids A‒F, cyathane diterpenoids with neurotrophic activity from cultures of the fungus *Cyathus striatus*. J. Nat. Prod..

[11] Shi XW, Liu L, Gao JM, Zhang AL (2011). Cyathane diterpenes from Chinese mushroom *Sarcodon scabrosus* and their neurite outgrowth-promoting activity. Eur. J. Med. Chem..

[12] Zhang CC (2015). Chemical constituents from the mushroom *Hericium erinaceus* and their ability to stimulate NGF-mediated neurite outgrowth on PC12 cells. Bioorg. Med. Chem. Lett..

[13] Gao JM (2007). Ergosterol peroxides as phospholipase A_2_ inhibitors from the fungus *Lactarius hatsudake*. Phytomedicine.

[14] Liu HW, Hu L, Zhang AL, Gao JM (2013). 2013. Steroids and phenolic constituents from the fruiting bodies of the basidiomycete *Sarcodon joedes*. Nat. Prod. Res..

[15] Liu L, Shi XW, Zong SC, Tang JJ, Gao JM (2012). Scabronine M, a novel inhibitor of NGF-induced neurite outgrowth from PC12 Cells from the fungus *Sarcodon scabrosus*. Bioorg. Med. Chem. Lett..

[16] Lu QQ, Tian JM, Wei J, Gao JM (2014). Bioactive metabolites from the mycelia of the basidiomycete *Hericium erinaceum*. Nat. Prod. Res..

[17] Zhang C-C (2017). Chemical Constituents from *Hericium erinaceus* Promote Neuronal Survival and Potentiate Neurite Outgrowth via the TrkA/Erk1/2 Pathway. Int. J. Mol. Sci..

[18] Wei J (2017). Molecular diversity and potential anti-neuroinflammatory activities of cyathane diterpenoids from the Basidiomycete Cyathus africanus. Sci. Rep..

[19] Gao JM, Yang SX, Qin JC (2013). Azaphilones: chemistry and biology. Chem. Rev..

[20] Li X-J, Zhang Q, Zhang A-L, Gao J-M (2012). Metabolites from *Aspergillus fumigatus*, an endophytic fungus associated with *Melia azedarach*, and their antifungal, antifeedant, and toxic activities. J. Agric. Food Chem..

[21] Xiao, J. *et al*. Secondary metabolites from the endophytic *Botryosphaeria dothidea* of *Melia azedarach* and their antifungal, antibacterial, antioxidant, and cytotoxic activities. *J. Agric. Food Chem.* **62**, 3584–3590 (2014). 10.1021/jf500054f 24689437

[22] Han J (2015). Three new cyathane diterpenoids from the medicinal fungus *Cyathus africanus*. J. Asian Nat. Prod. Res..

[23] Shiono Y, Hiramatsu F, Murayama T, Koseki T, Funakoshi T (2008). Two cyathane-type diterpenoids from the liquid culture of *Strobilurus tenacellus*. Chem. Biodivers..

[24] Ayer WA, Lee SP (1979). Metabolites of bird’s nest fungi. Part 11. Diterpenoid metabolites of *Cyathus earlei*. Can. J. Chem..

[25] Wang B, Han J, Xu W, Chen Y, Liu H (2014). Production of bioactive cyathane diterpenes by a bird’s nest fungus *Cyathus gansuensis* growing on cooked rice. Food Chemistry.

[26] Pescitelli G, Bruhn T (2016). Good computational practice in the assignment of absolute configurations by TDDFT calculations of ECD spectra. Chirality.

[27] Autschbach J, Nitsch-Velasquez L, Rudolph M (2011). Time-dependent density functional response theory for electronic chiroptical properties of chiral molecules. Top. Curr. Chem..

[28] Mennucci B, Cappelli C, Cammi R, Tomasi J (2011). Modeling solvent effects on chiroptical properties. Chirality.

[29] Bruhn, T., Schaumlöffel, A., Hemberger, Y. & Pescitelli, G. SpecDis version 1.70, Berlin, Germany, https://specdis-software.jimdo.com/ (2017).

[30] Shibata H (1989). Studies on chemical components of mushrooms. Part I. Isolation and characterization of new bitter diterpenoids from the fungus *Sarcodon scabrosus*. Agric. Biol. Chem..

[31] Xu Z, Yan S, Bi K, Liu H (2013). Isolation and identification of a new anti-inflammatory cyathane diterpenoid from the medicinal fungus *Cyathus hookeri* Berk. Fitoterapia.

[32] Zeng KW, Zhao MB, Ma ZZ, Jiang Y, Tu PF (2012). Protosappanin A inhibits oxidative and nitrative stress via interfering the interaction of transmembrane protein CD14 with Toll-like receptor-4 in lipopolysaccharide-induced BV-2 microglia. Int. Immunopharmacol..

[33] Li D, Chi B, Wang WW, Gao JM, Wan J (2017). Exploring the possible binding mode of trisubstituted benzimidazoles analogues *in silico* for novel drug design targeting Mtb FtsZ. Med. Chem. Res..

[34] Frisch, M. J. *et al*. Revision A.03, Gaussian, Inc.: Wallingford CT, 2016.

