# Supplementary material for: Polyoxygenated cyathane diterpenoids from the mushroom Cyathus africanus, and their neurotrophic and anti-neuroinflammatory activities
Source: Sci Rep. 2018 Feb 1;8:2175. doi: 10.1038/s41598-018-20472-4 (PMC5794895; doi:10.1038/s41598-018-20472-4)
Supplement: Supplementary file 1 — SUPPLEMENTARY INFORMATION [file 41598_2018_20472_MOESM1_ESM.pdf]

Polyoxygenated cyathane diterpenoids from the mushroom *Cyathus africanus*, and their neurotrophic and anti-neuroinflammatory activities

Jing Wei,[1,3] Wan-Hui Guo,[1] Chen-Yu Cao,[1] Rong-Wei Kou,[1] Yuan-Zhen Xu,[1] Marcin Górecki,[2] Lorenzo Di Bari,[2] Gennaro Pescitelli,*[,2] & Jin-Ming Gao*[,1]

[1] Shaanxi Key Laboratory of Natural Products & Chemical Biology, College of Chemistry & Pharmacy, Northwest A&F University, Yangling 712100, Shaanxi, People's Republic of China.

[2] Dipartimento di Chimica e Chimica Industriale, Universit`a di Pisa, via Moruzzi 3, I-56124 Pisa, Italy.

[3] College of Biology Pharmacy & Food Engineering, Shangluo University, Shangluo 726000, Shaanxi, People's Republic of China.

Content

Figure S1. ^{1}H NMR and ^{13}C spectra of compound **1** in CD_3OD......3
Figure S2. HSQC and ^{1}H-^{1}H COSY spectra of compound **1**in CD_3OD......4
Figure S3. HMBC and NOESY spectra of compound **1** in CD_3OD......5
Figure S4. HRESIMS spectrum of compound **1**......5
Figure S5. IR and UV spectra of compound **1**......6
Figure S6. ^{1}H NMR and ^{13}C spectra of compound **2** in CD_3OD......7
Figure S7. HSQC and ^{1}H-^{1}H COSY spectra of compound **2**in CD_3OD......8
Figure S8. HMBC and NOESY spectra of compound **2** in CD_3OD......9
Figure S9. HRESIMS spectrum of compound **2**......10
Figure S10. IR and UV spectra of compound **2**......10
Figure S11. ^{1}H NMR and ^{13}C spectra of compound **3** in CD_3OD......11
Figure S12. HSQC and ^{1}H-^{1}H COSY spectra of compound **3**in CD_3OD......12
Figure S13. HMBC and NOESY spectra of compound **3** in CD_3OD......13
Figure S14. HRESIMS spectrum of compound **3**......14
Figure S15. IR and UV spectra of compound **3**......15

Figure S16. ^{1}H NMR and ^{13}C spectra of compound **4** in CD_3OD. 16
Figure S17. HSQC and ^{1}H-^{1}H COSY spectra of compound **4**in CD_3OD. 17
Figure S18. HMBC and NOESY spectra of compound **4** in CD_3OD. 18
Figure S19. HRESIMS spectrum of compound **4**. 19
Figure S20. IR and UV spectra of compound **4**. 19
Figure S21. ^{1}H NMR and ^{13}C spectra of compound **5** in CD_3OD. 20
Figure S22. HSQC and ^{1}H-^{1}H COSY spectra of compound **5** in CD_3OD. 21
Figure S23. HMBC and NOESY spectra of compound **5** in CD_3OD. 22
Figure S24. HRESIMS spectrum of compound **5**. 23
Figure S25. IR and UV spectra of compound **5**. 24
Figure S26. ^{1}H NMR and ^{13}C spectra of compound **6** in CD_3OD. 25
Figure S27. HSQC and ^{1}H-^{1}H COSY spectra of compound **6** in CD_3OD. 26
Figure S28. HMBC and NOESY spectra of compound **6** in CD_3OD. 27
Figure S29. HRESIMS spectrum of compound **6**. 28
Figure S30. IR and UV spectra of compound **6**. 28
Figure S31. ^{1}H NMR and ^{13}C spectra of compound **7** in CD_3OD. 29
Figure S32. HSQC and ^{1}H-^{1}H COSY spectra of compound **7** in CD_3OD. 30
Figure S33. HMBC and NOESY spectra of compound **7** in CD_3OD. 31
Figure S34. HRESIMS spectrum of compound **7**. 32
Figure S35. IR and UV spectra of compound**7**. 33
Figure S36. ^{1}H NMR and ^{13}C spectra of compound **8** in CD_3OD. 34
Figure S37. HSQC and ^{1}H-^{1}H COSY spectra of compound **8** in CD_3OD. 35
Figure S38. HMBC and NOESY spectra of compound **8** in CD_3OD. 36
Figure S39. HRESIMS spectrum of compound **8**. 37
Figure S40. IR and UV spectra of compound **8**. 37
Figure S41. ^{1}H NMR and ^{13}C spectra of compound **9** in CD_3OD. 38
Figure S42. HSQC and ^{1}H-^{1}H COSY spectra of compound **9** in CD_3OD. 39
Figure S43. HMBC and NOESY spectra of compound **9** in CD_3OD. 40
Figure S44. HRESIMS spectrum of compound **9**. 42
Figure S45. IR and UV spectra of compound **9**. 42
Figure S46. ^{1}H NMR and ^{13}C spectra of compound **10** in CD_3OD. 43
Figure S47. ESIMS spectrum of compound **10**. 44
Figure S48. ^{1}H NMR and ^{13}C spectra of compound **11** in CD_3OD. 44
Figure S49. ESIMS spectrum of compound **11**. 45
Figure S50. ^{1}H NMR and ^{13}C spectra of compound **6a** in CD_3CN. 46
Figure S51. HSQC and ^{1}H-^{1}H TOCSY spectra of compound **6a** in CD_3CN 47
Figure S52. HMBC and ROESY spectra of compound **6a** in CD_3CN 48
Figure S53. Cytotoxicity of the isolated compounds **1-10** to PC-12 cells 49
Figure S54. Effect of compound **1** on the NGF-promoted neurite outgrowth in PC-12 cells 49
Table S1. ^{1}H and ^{13}C data of **9-11**, and cyathin I in CD_3OD. 50
Table S2. Logarithms of Free Binding Energy of the NO Inhibitor 51

Figure S1. ^{1}H NMR and ^{13}C spectra of compound **1** in CD_3OD.

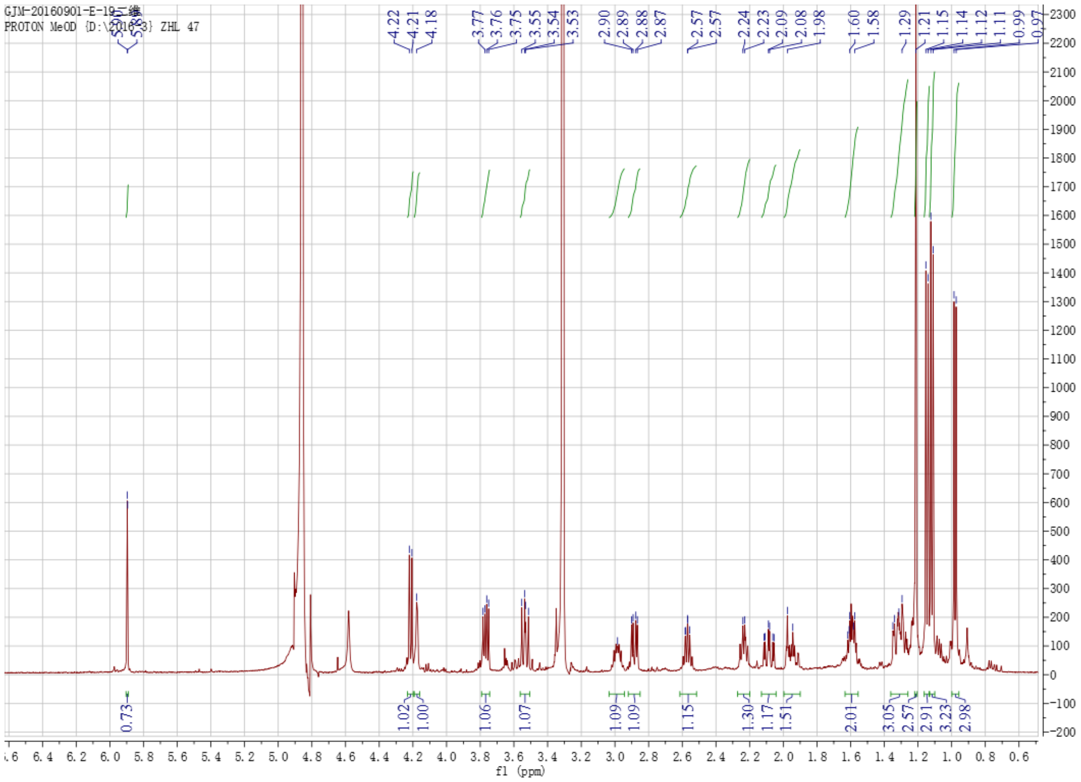

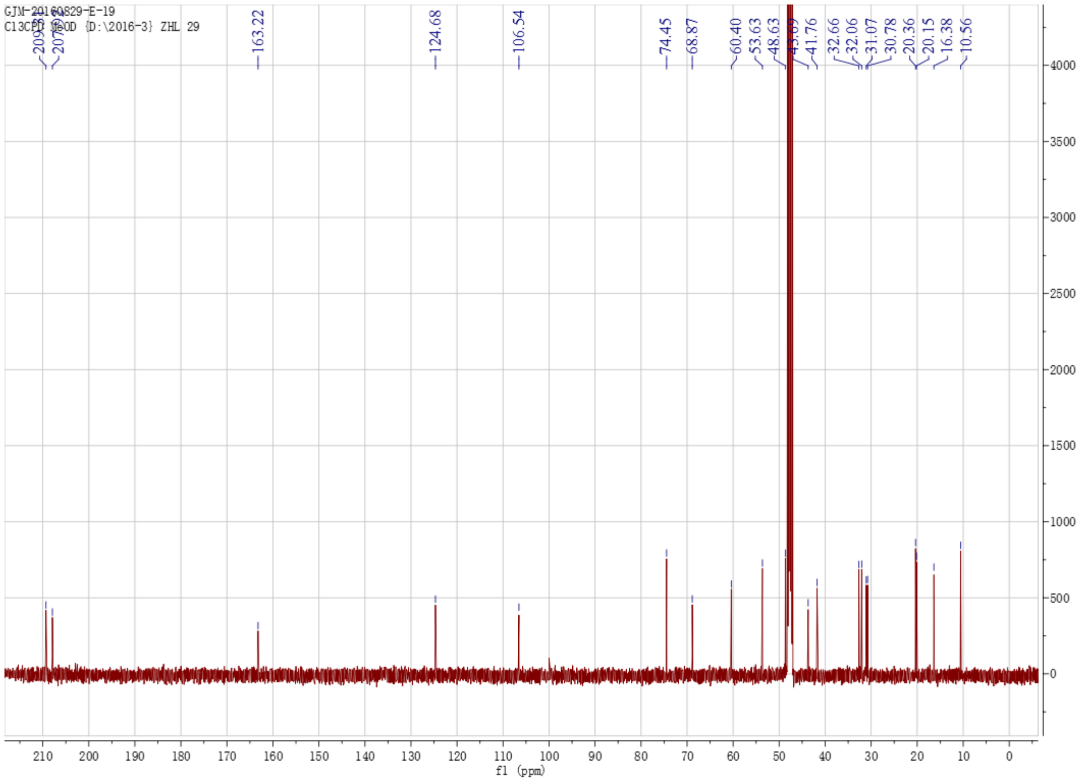

Figure S2. HSQC and ^{1}H-^{1}H COSY spectra of compound **1**in CD_3OD

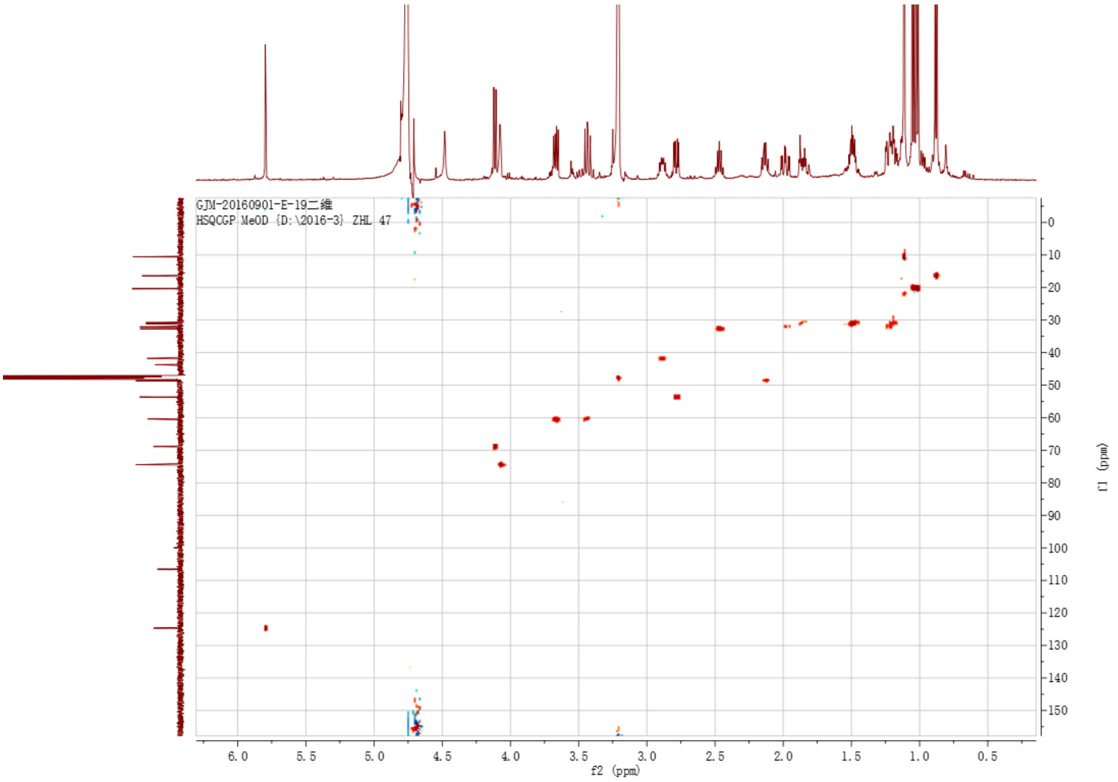

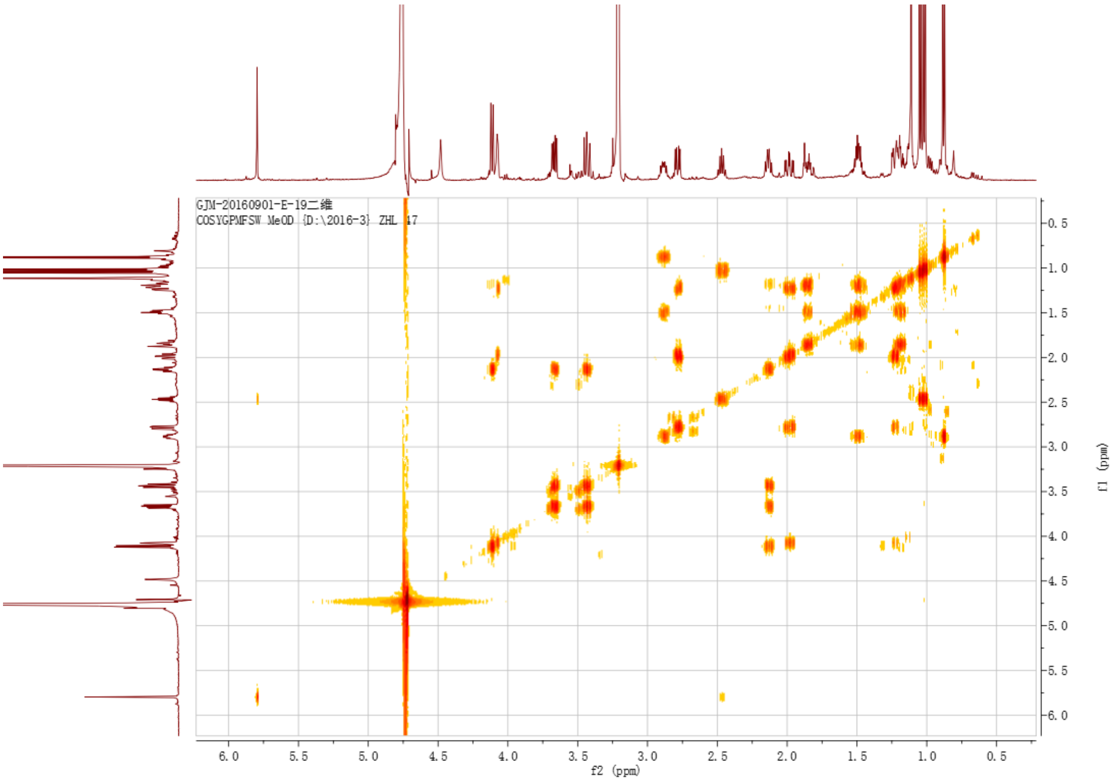

Figure S3. HMBC and NOESY spectra of compound **1** in CD_3OD

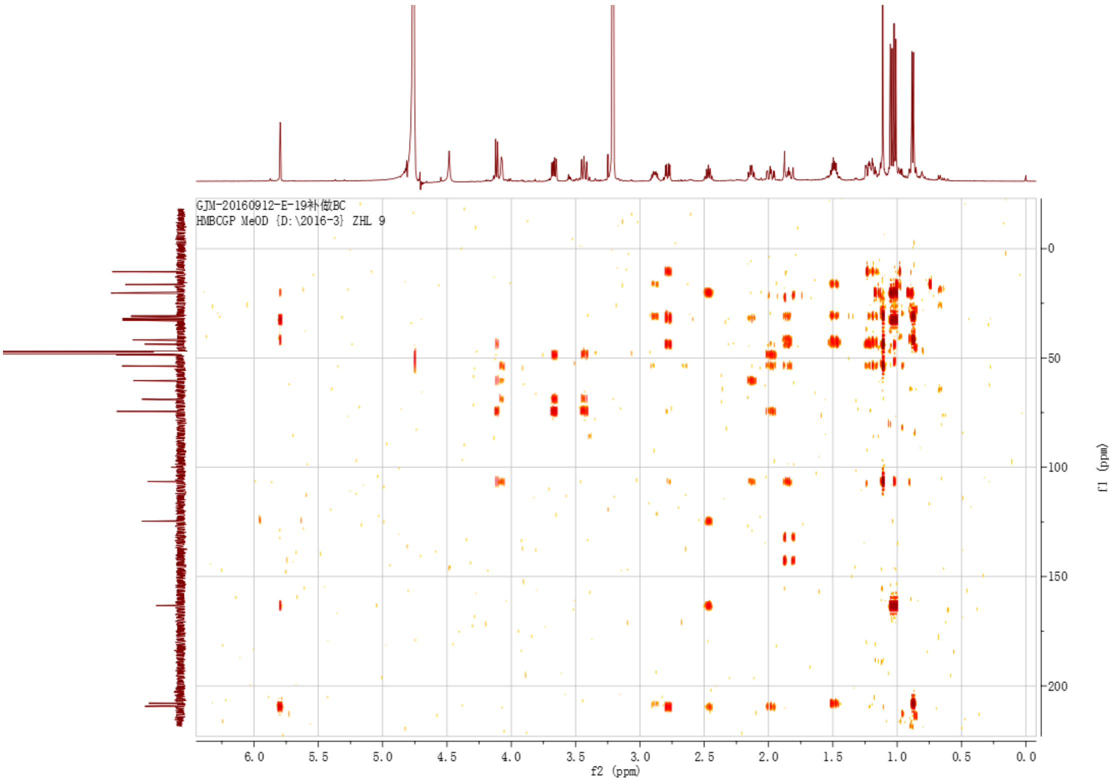

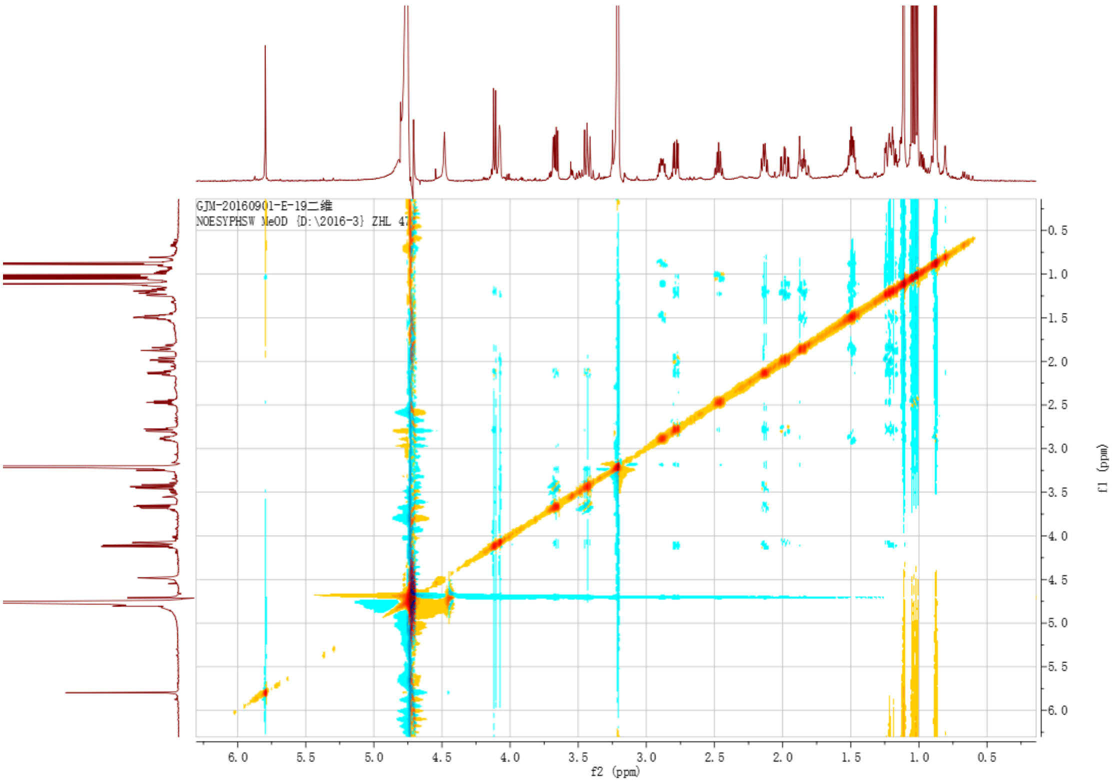

Figure S4. HRESIMS spectrum of compound **1**.

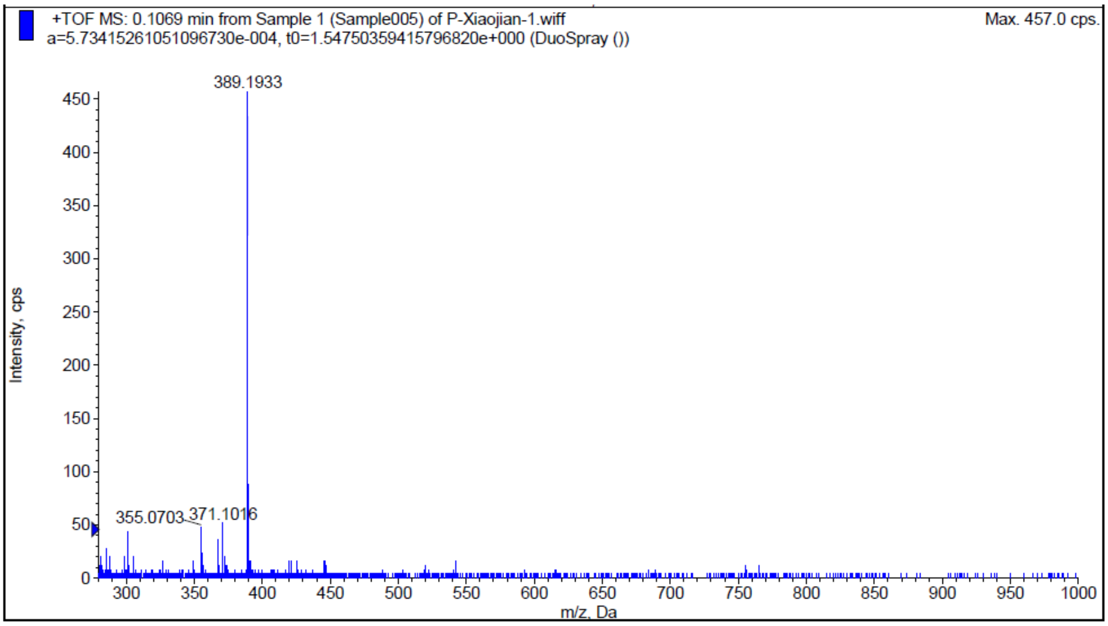

Figure S5. IR and UV spectra of compound **1**.

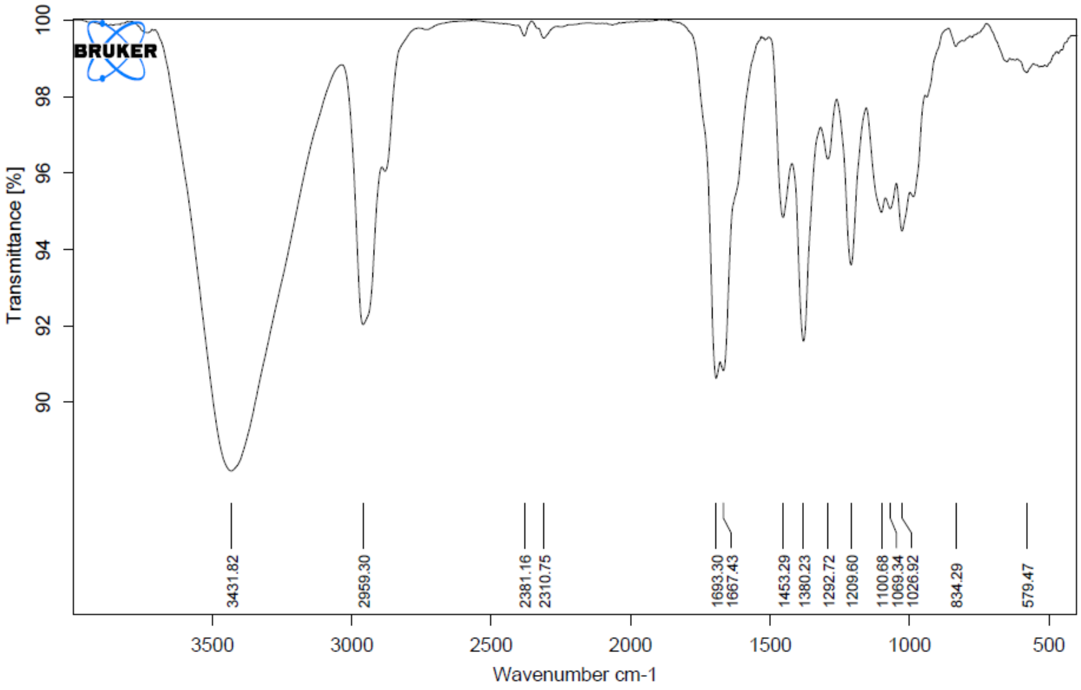

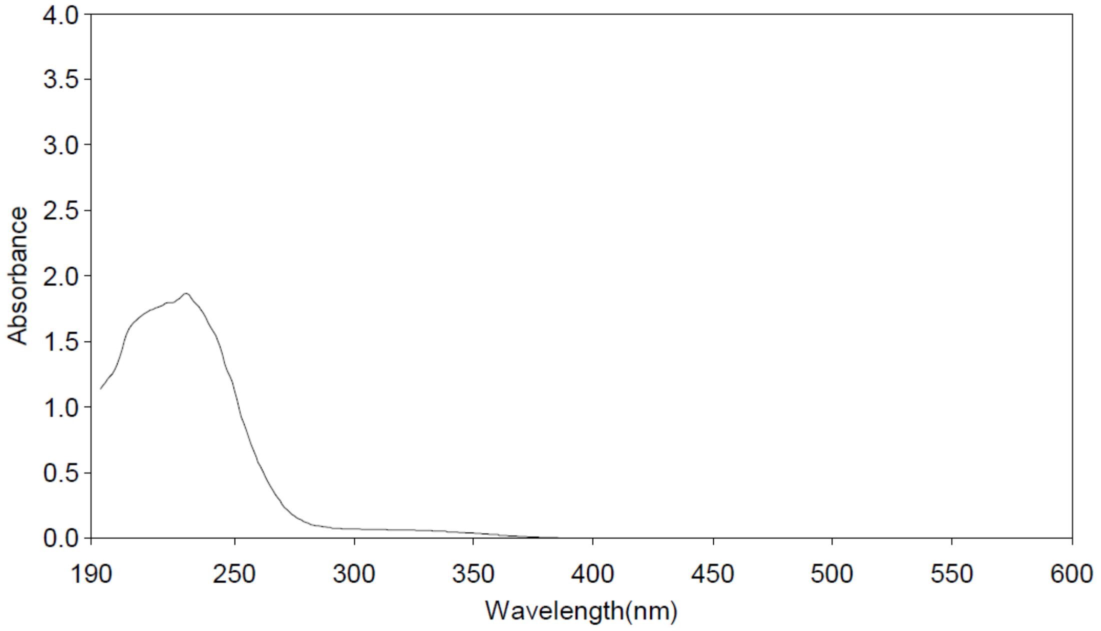

Figure S6. ^{1}H NMR and ^{13}C spectra of compound **2** in CD_3OD.

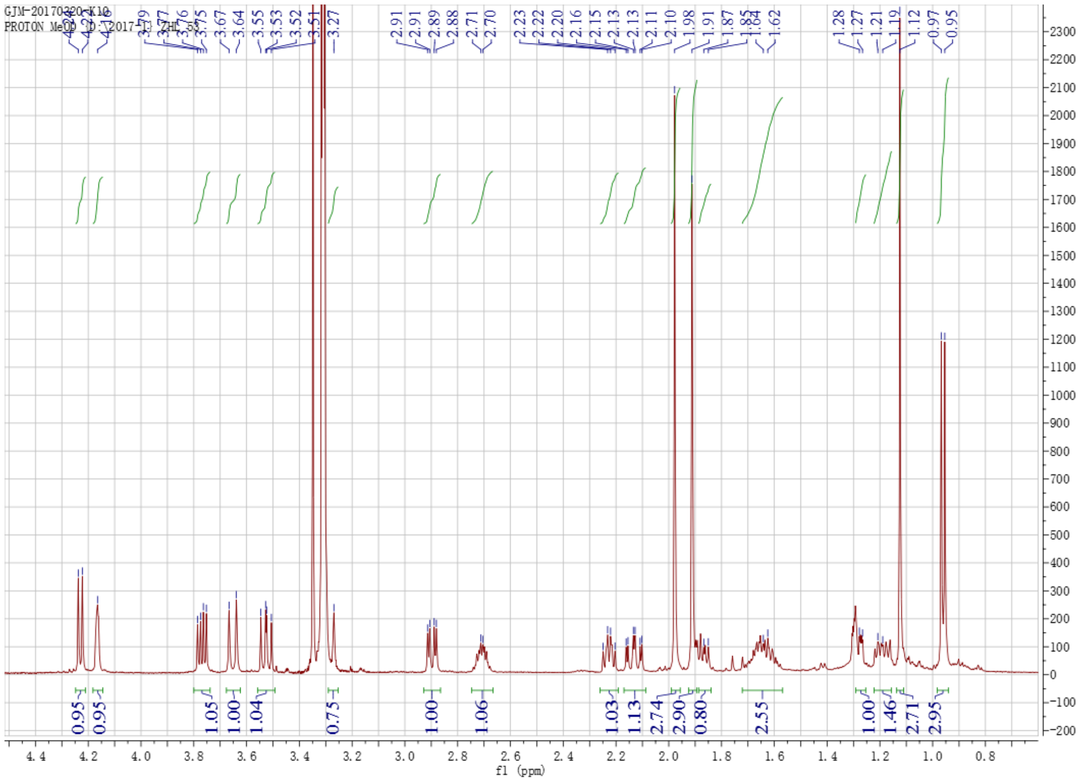

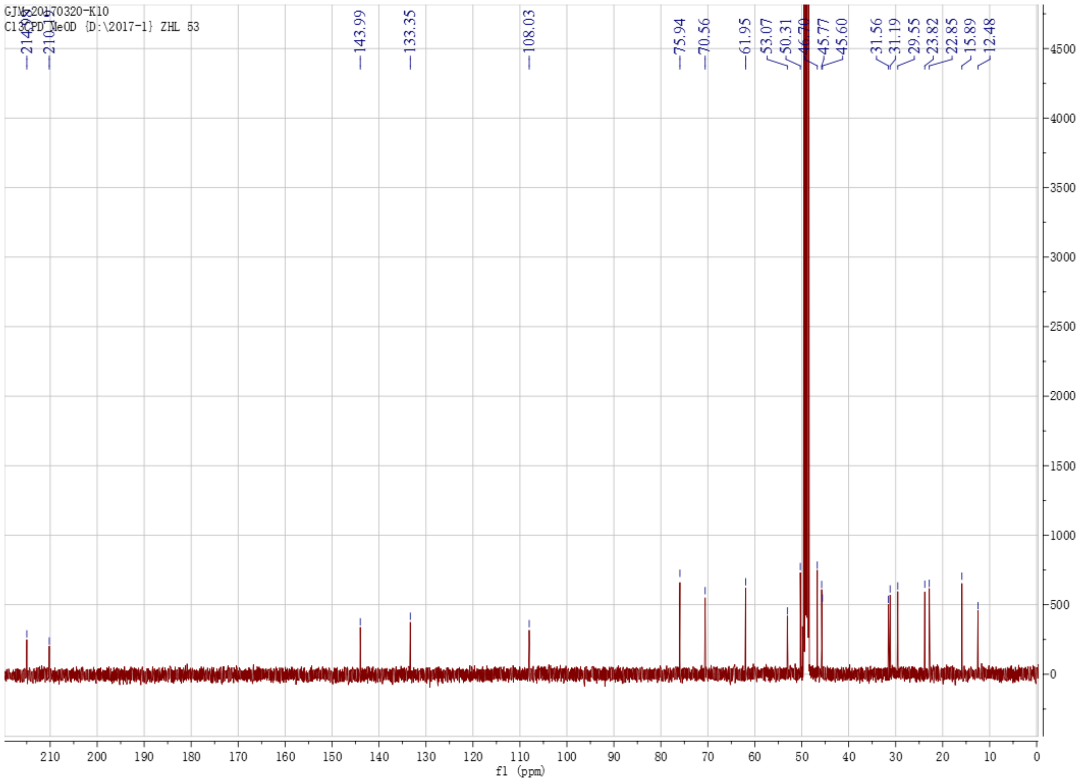

Figure S7. HSQC and ^{1}H-^{1}H COSY spectra of compound **2**in CD_3OD.

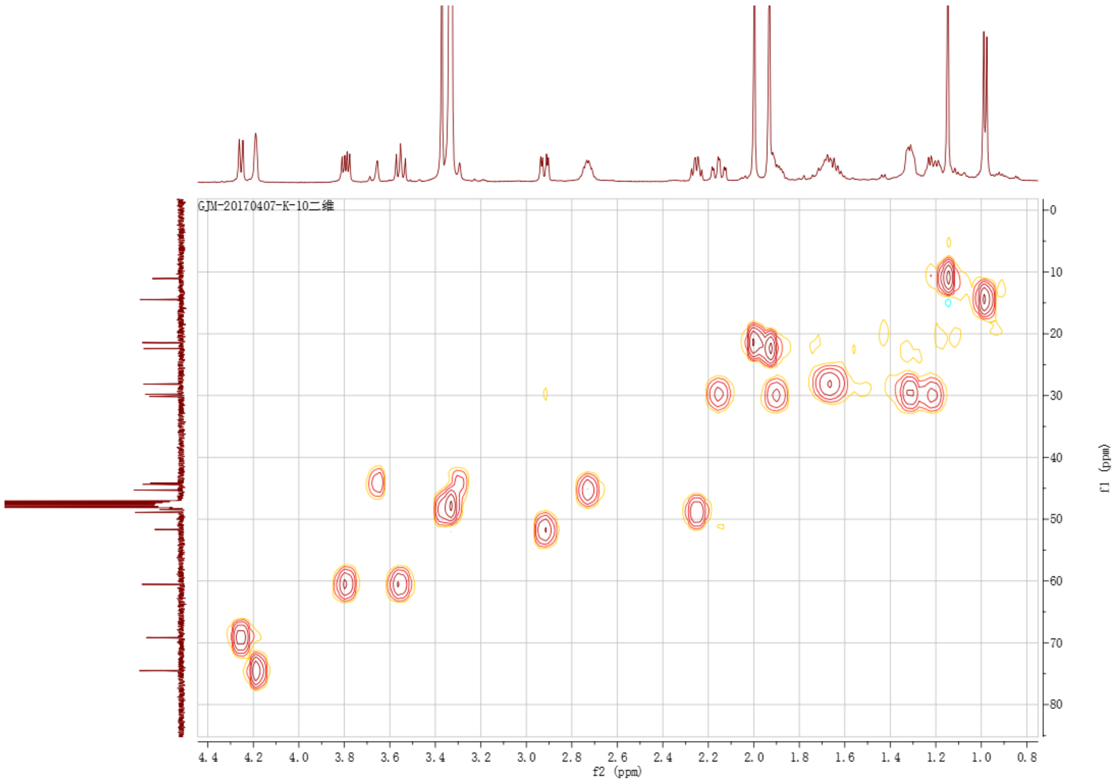

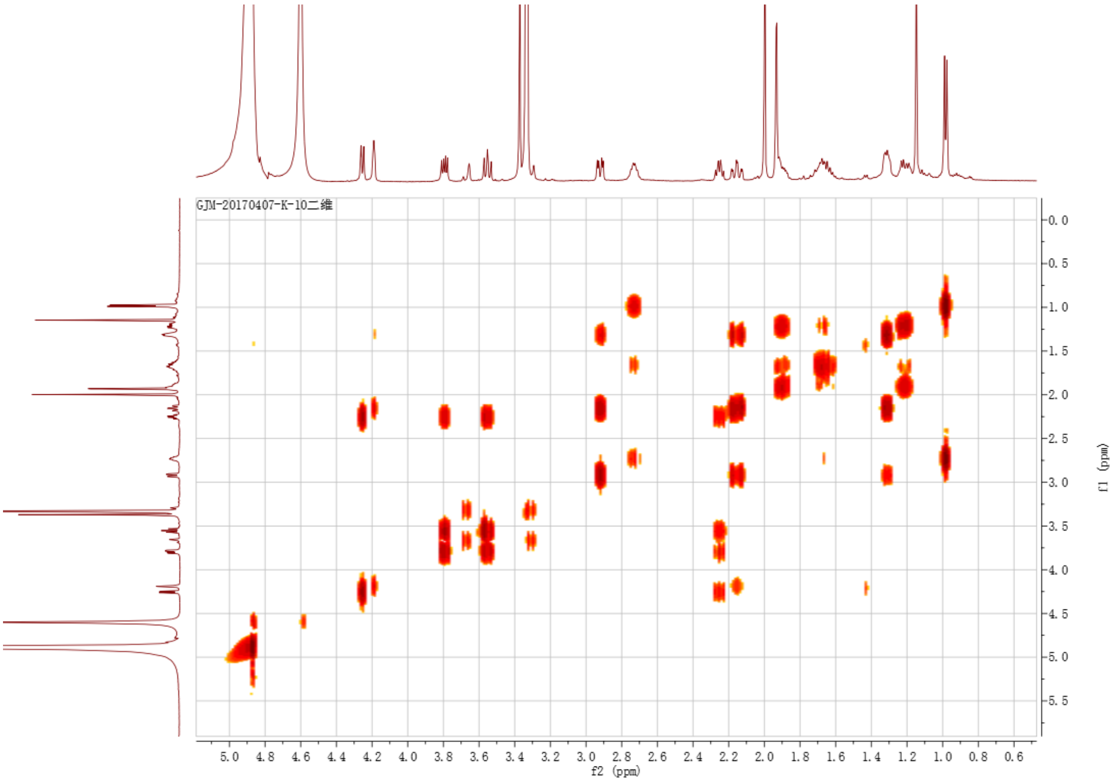

Figure S8. HMBC and NOESY spectra of compound **2** in CD_3OD.

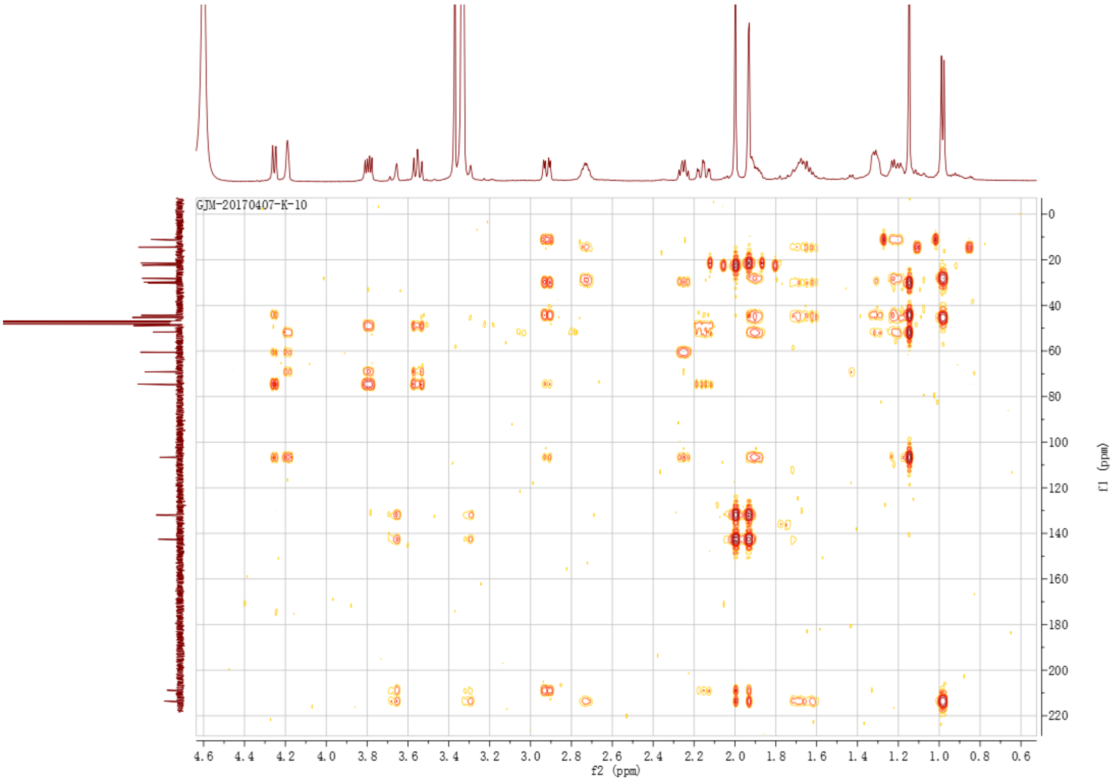

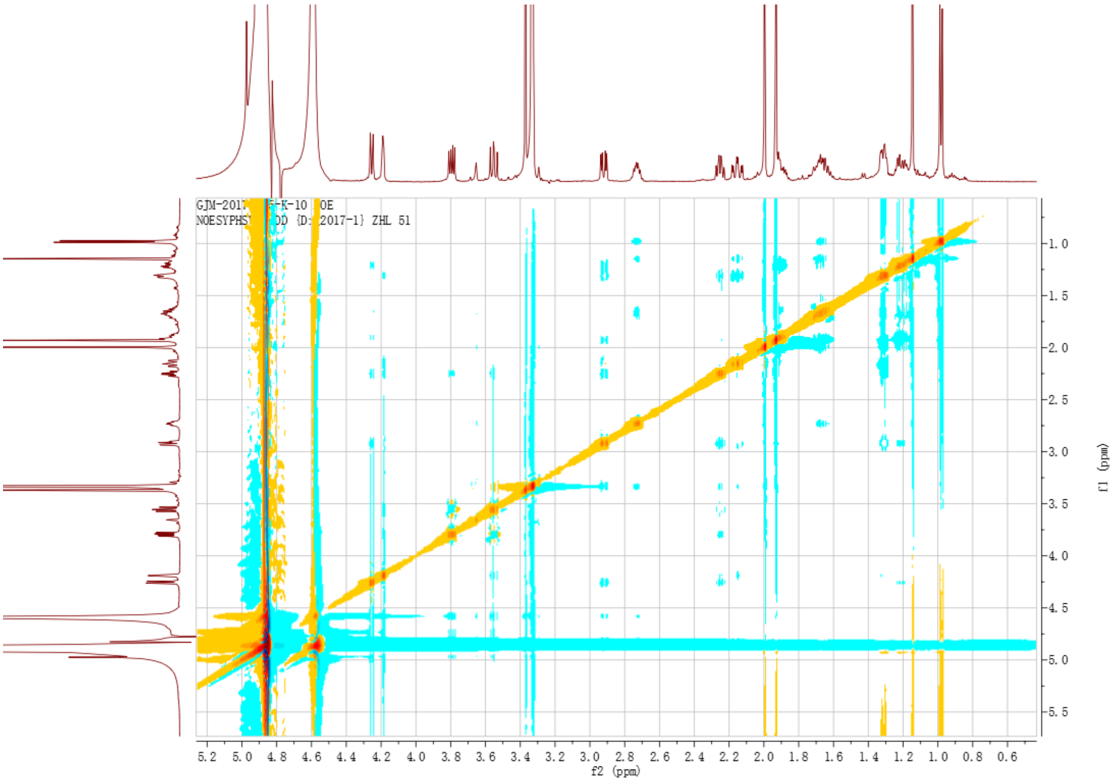

Figure S9. HRESIMS spectrum of compound **2**.

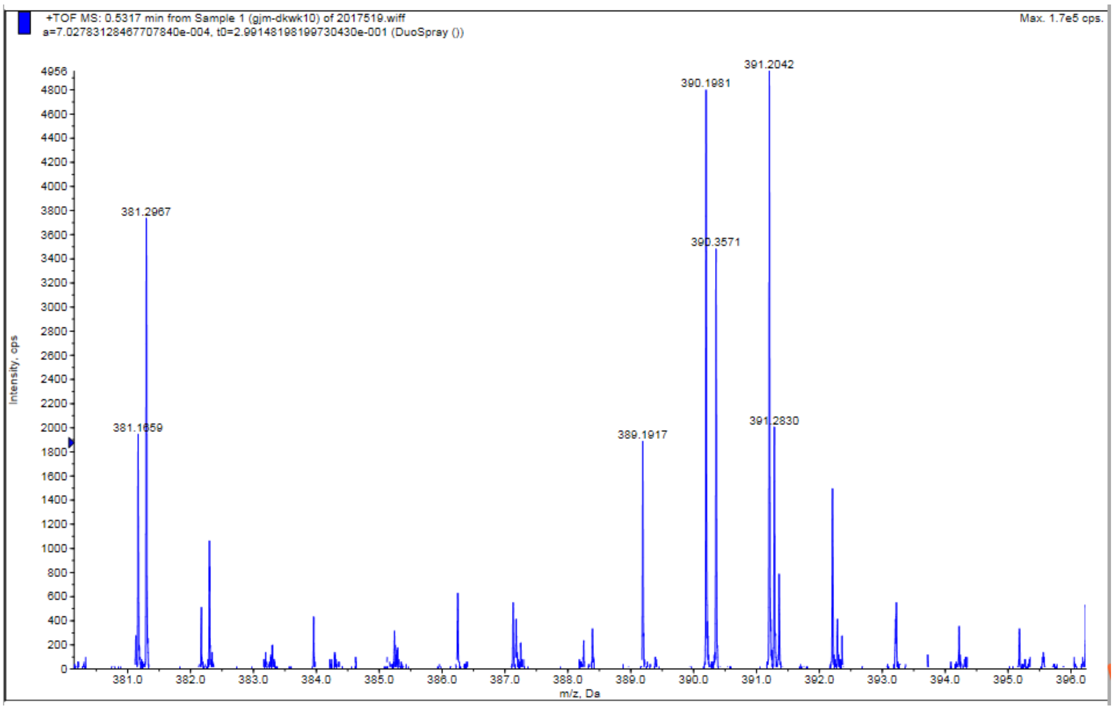

Figure S10. IR and UV spectra of compound **2**.

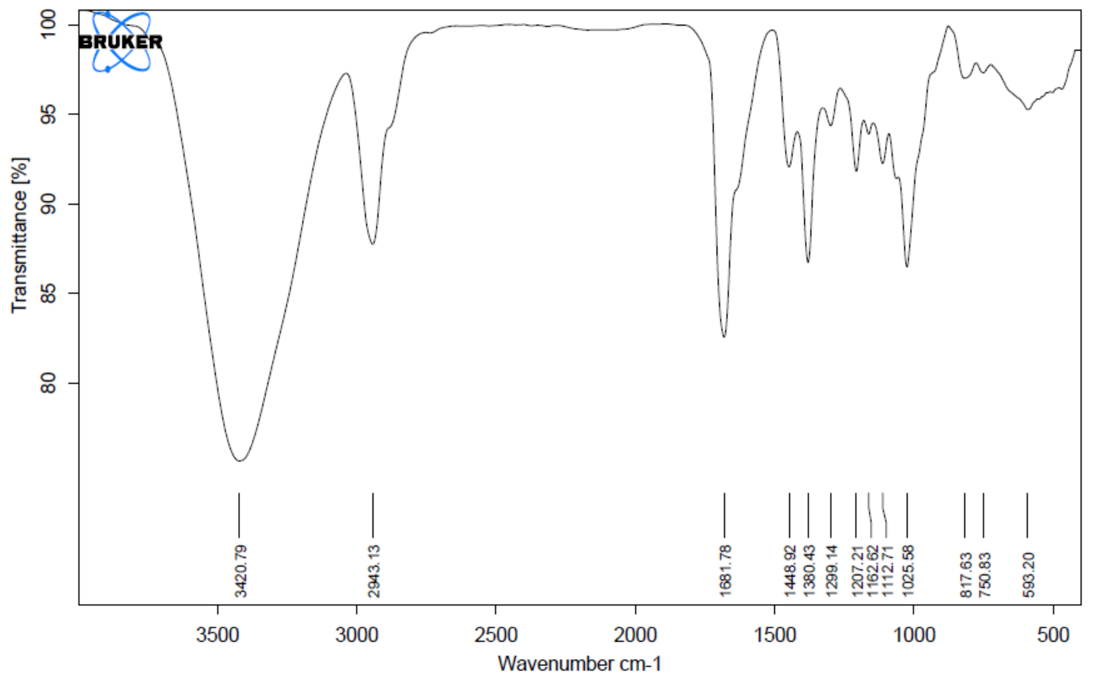

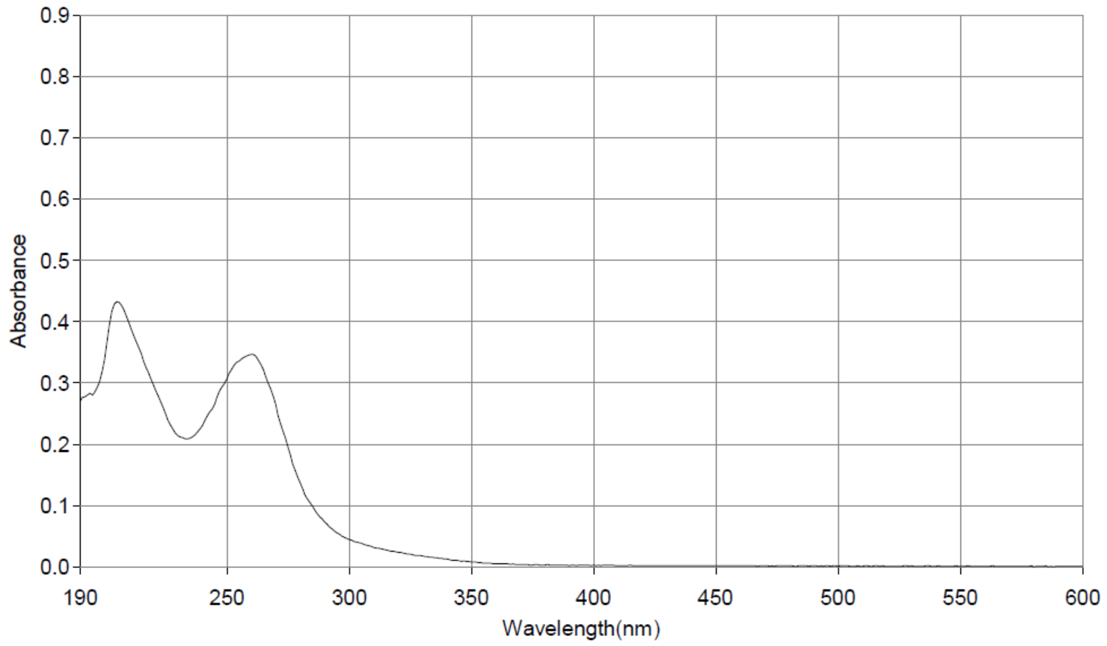

Figure S11. ^{1}H NMR and ^{13}C spectra of compound **3** in CD_3OD.

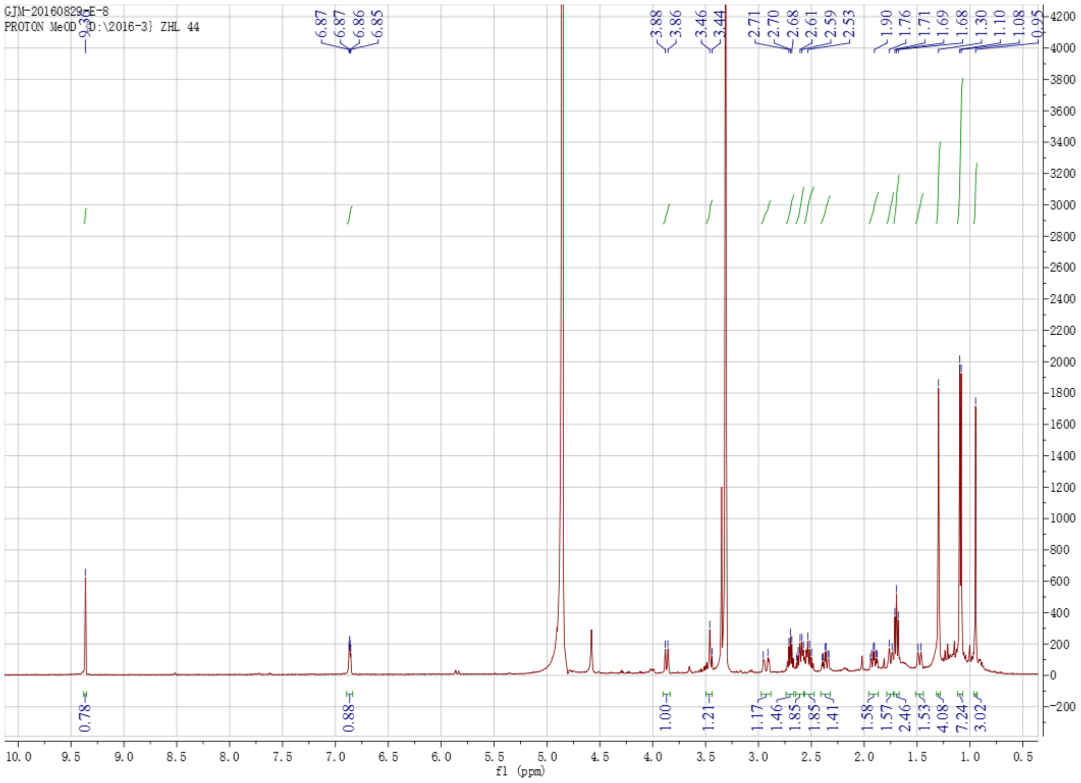

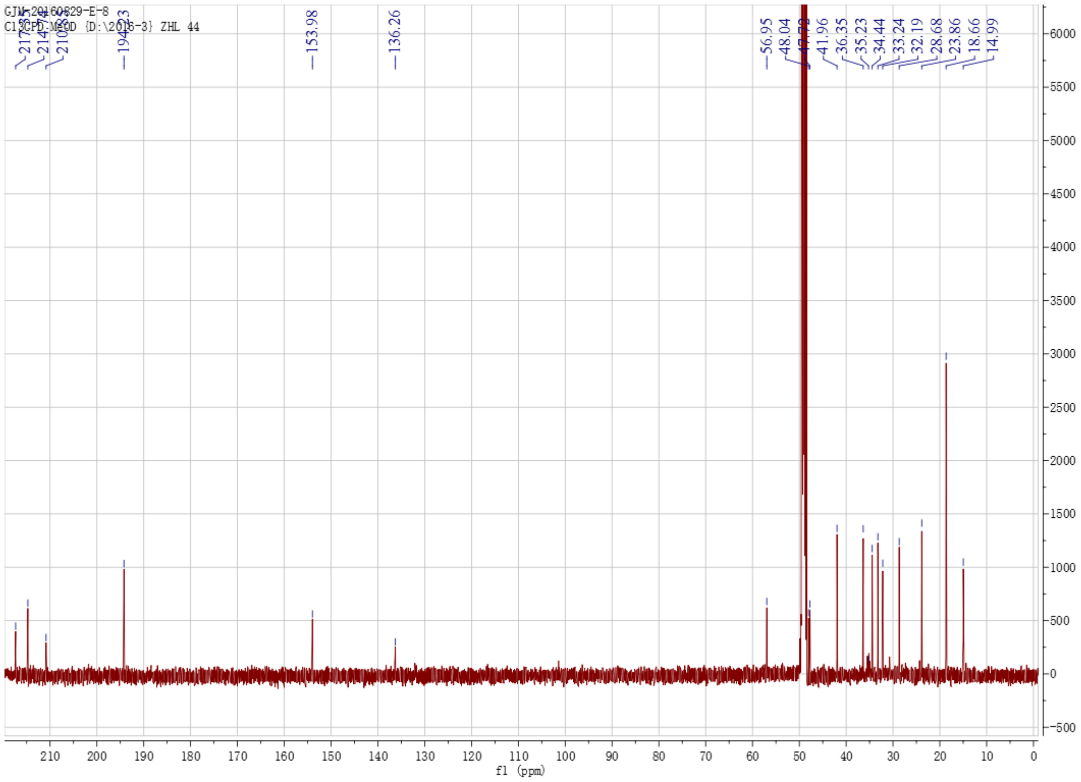

Figure S12. HSQC and ^{1}H-^{1}H COSY spectra of compound **3**in CD_3OD.

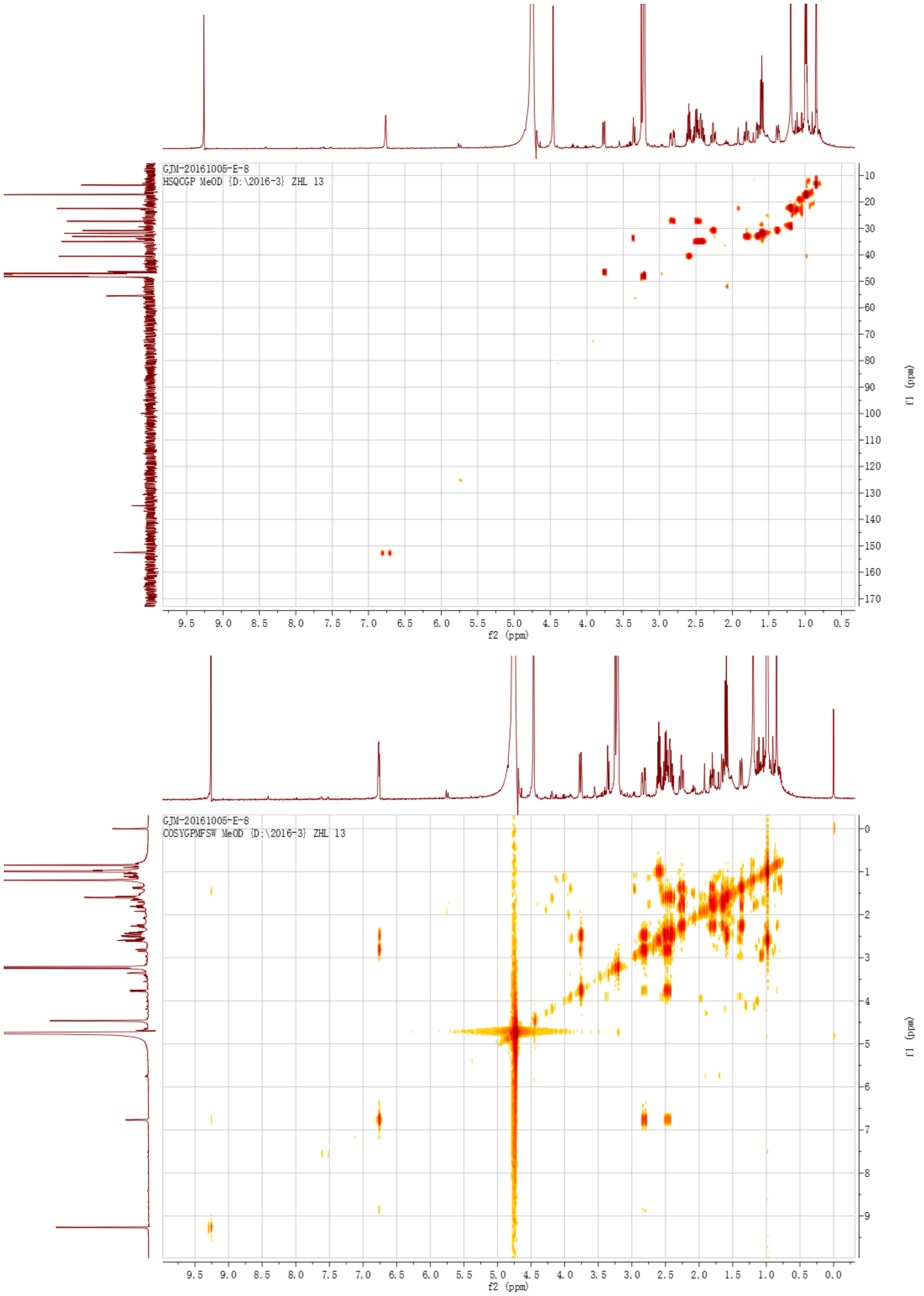

Figure S13. HMBC and NOESY spectra of compound **3** in CD_3OD.

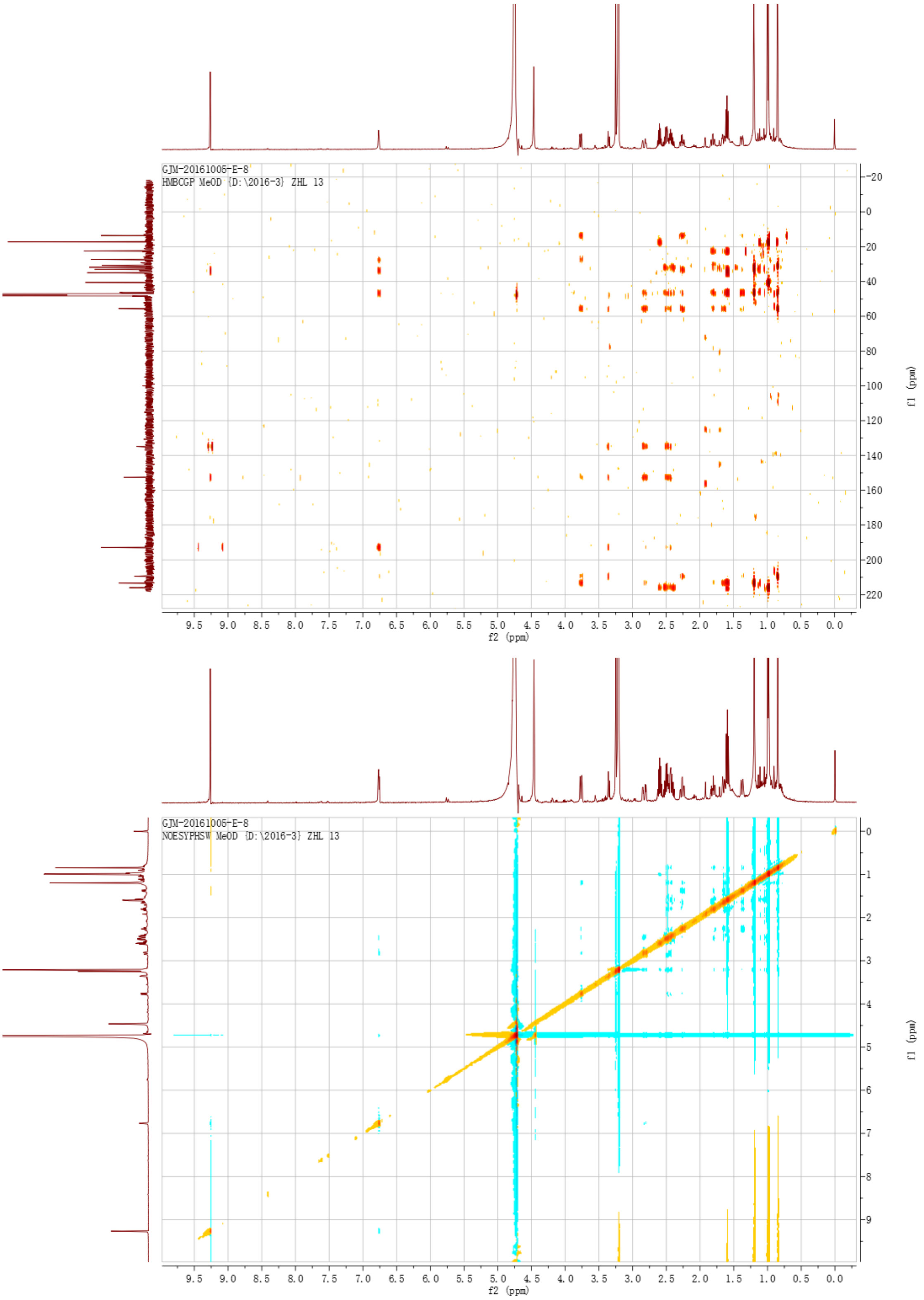

Figure S14. HRESIMS spectrum of compound **3**.

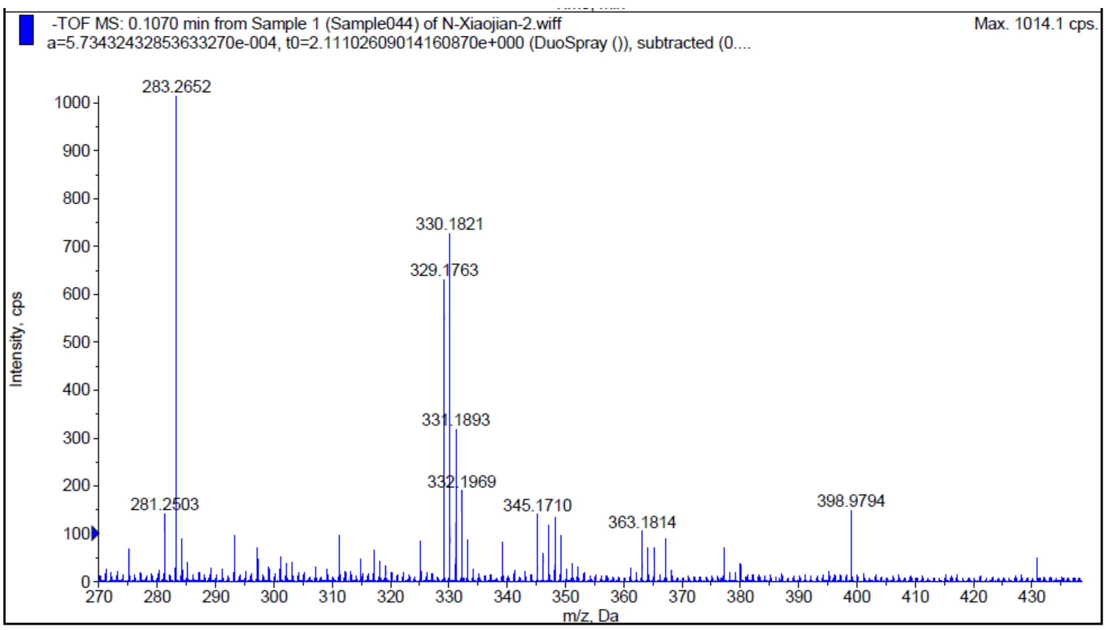

Figure S15. IR and UV spectra of compound **3**.

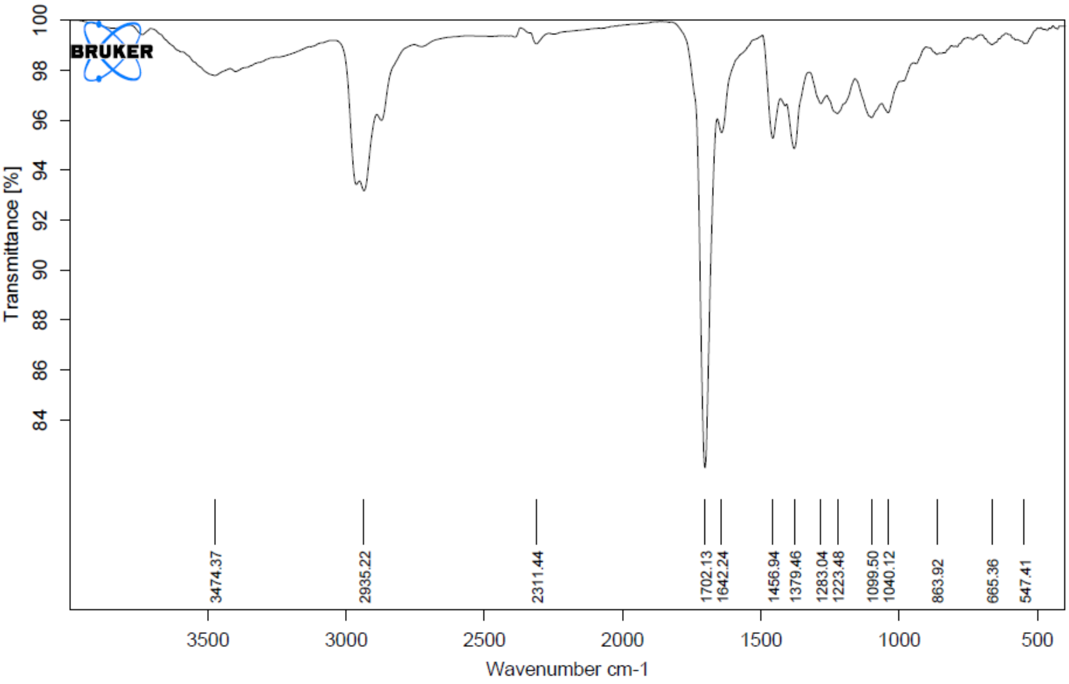

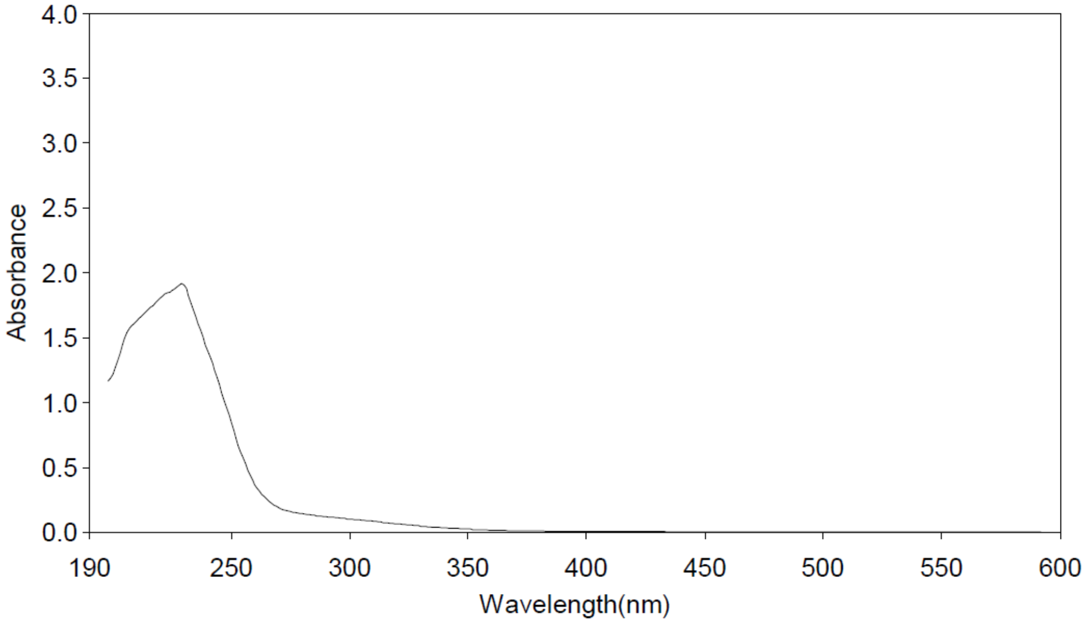

Figure S16. ^{1}H NMR and ^{13}C spectra of compound **4** in CD_3OD.

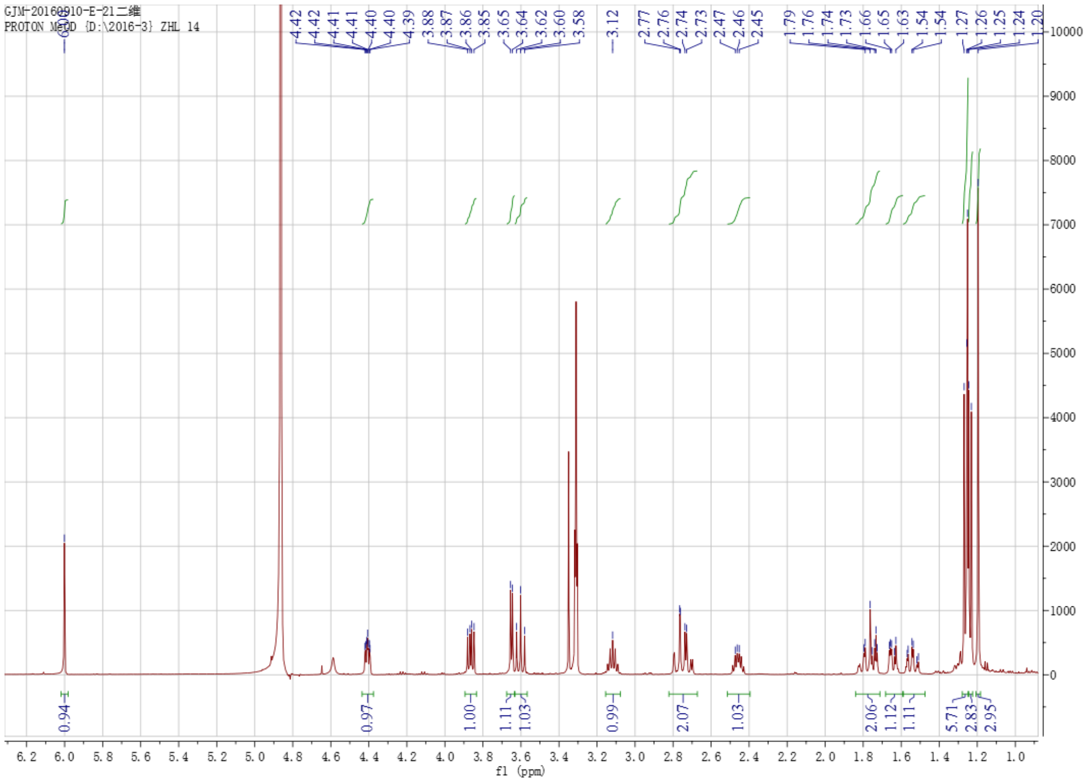

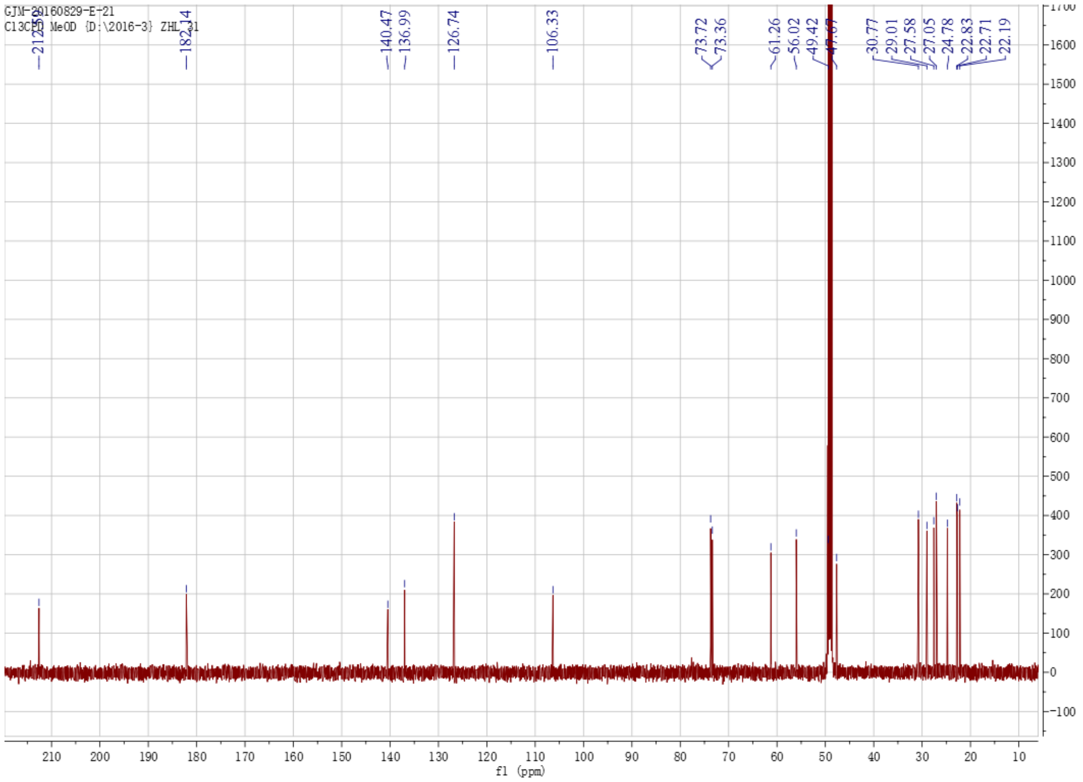

Figure S17. HSQC and ^{1}H-^{1}H COSY spectra of compound **4**in CD_3OD.

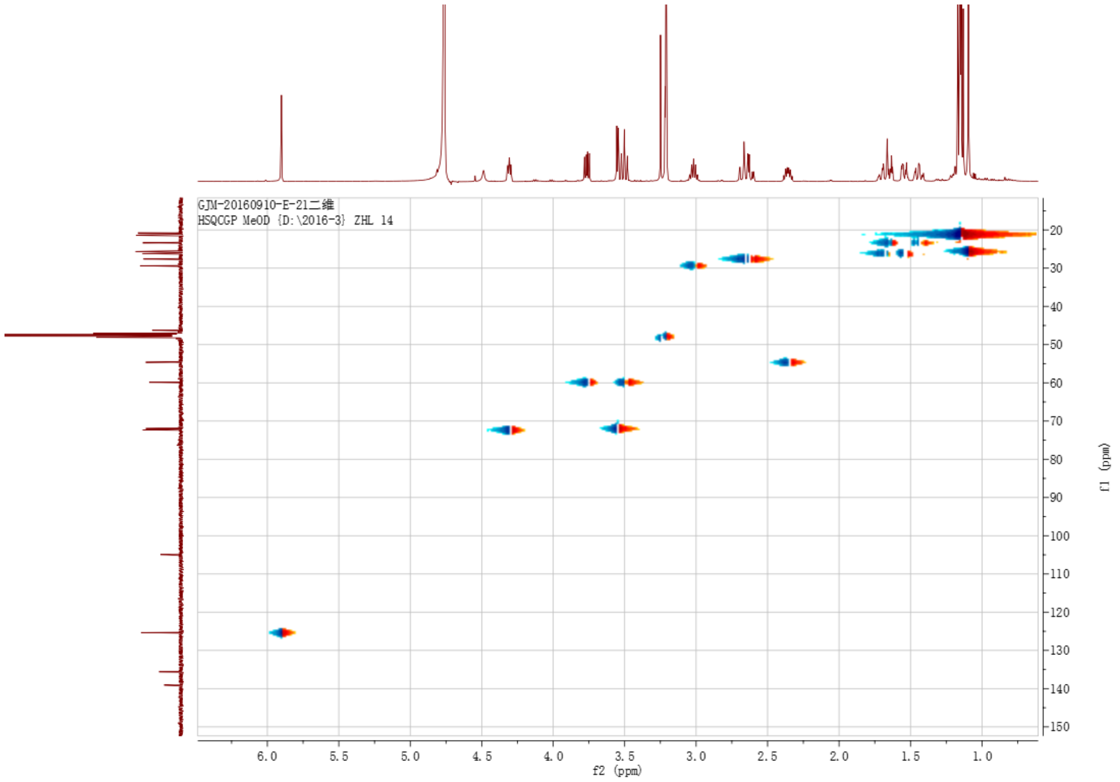

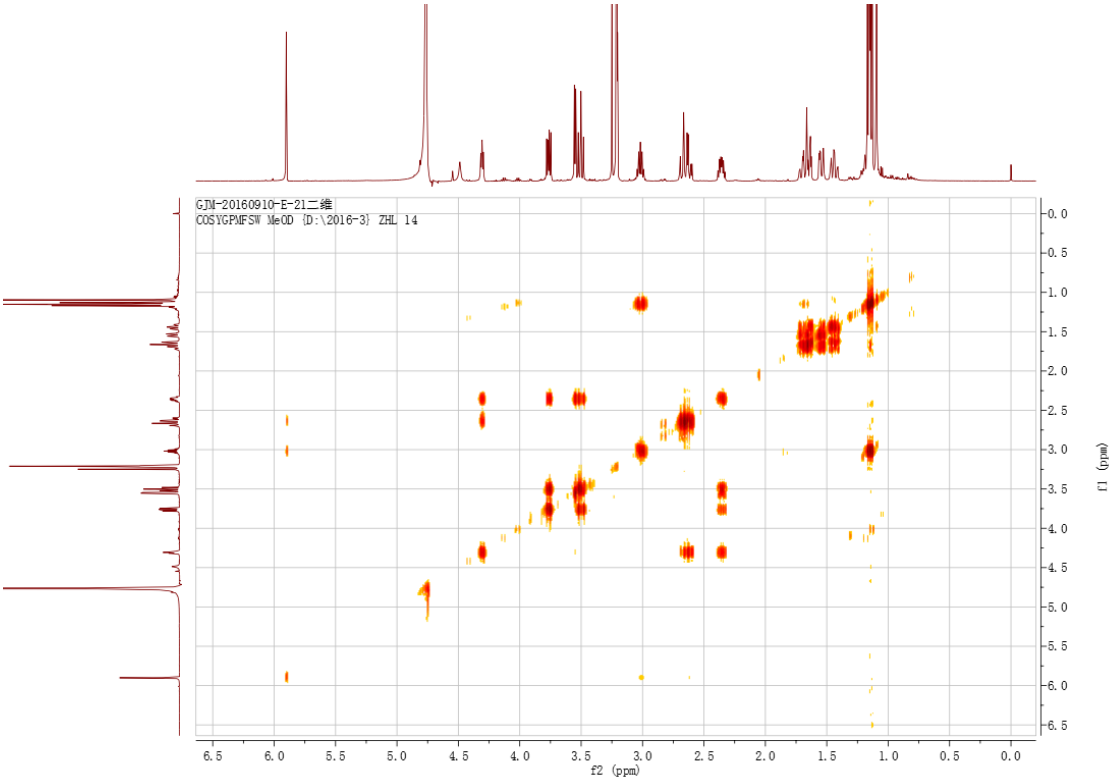

Figure S18. HMBC and NOESY spectra of compound **4** in CD_3OD.

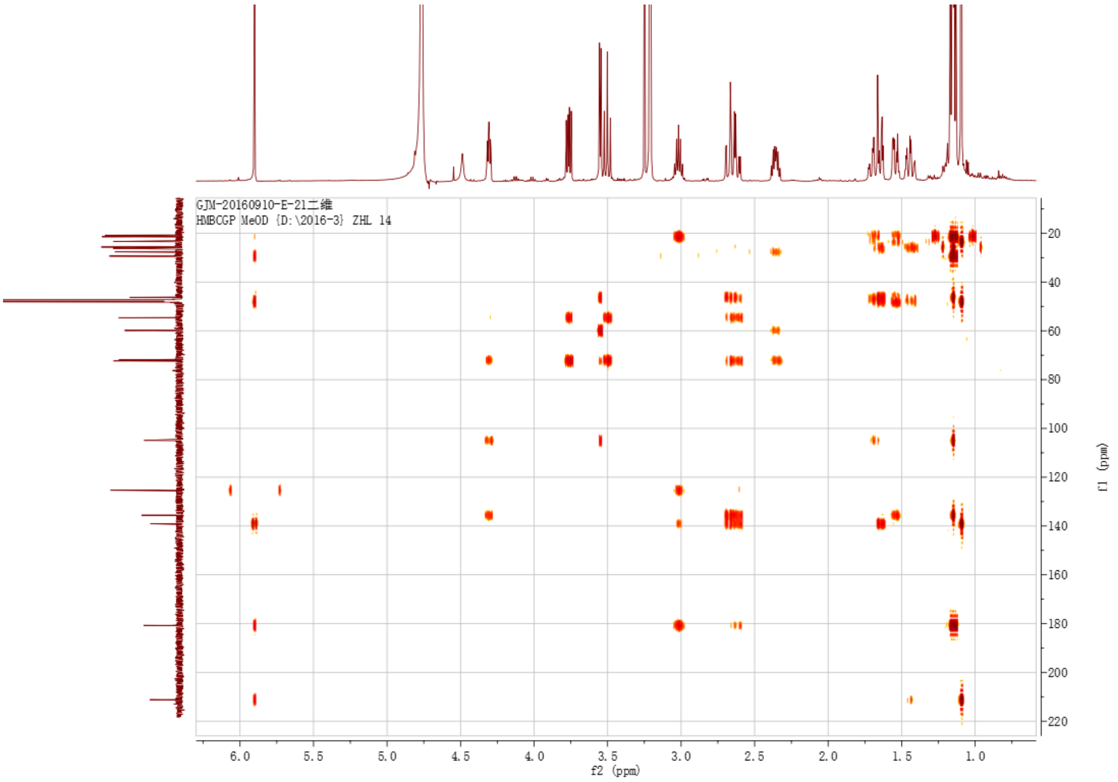

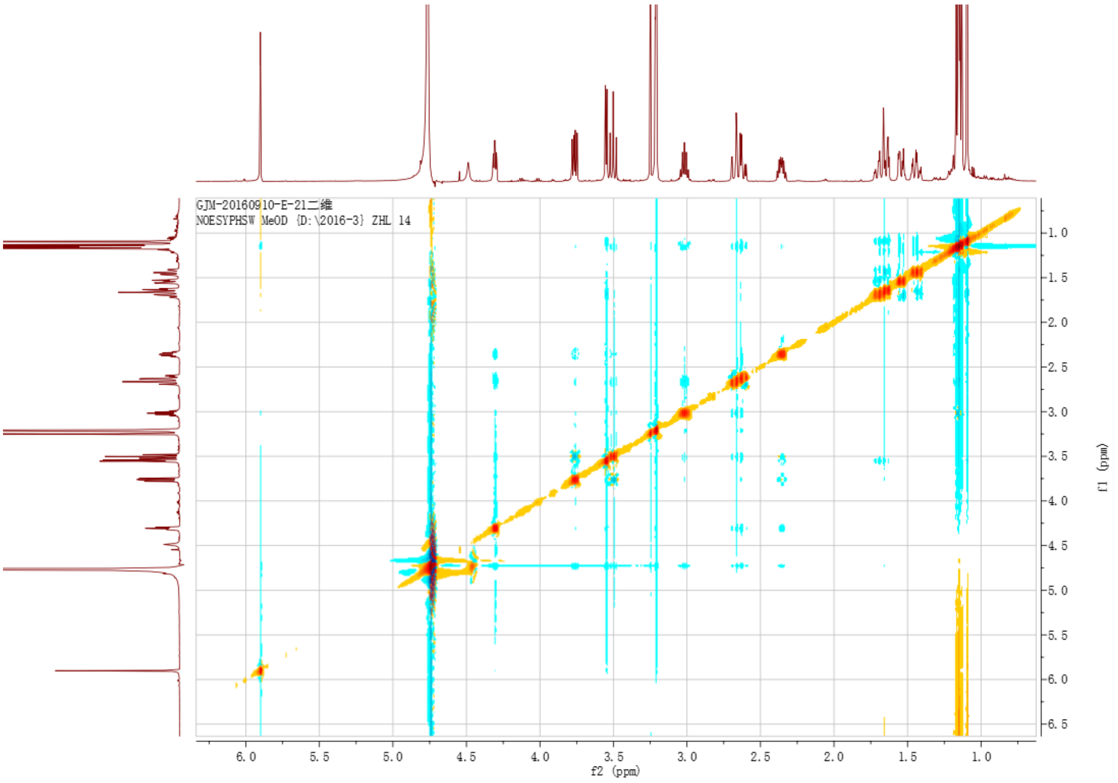

Figure S19. HRESIMS spectrum of compound **4**.

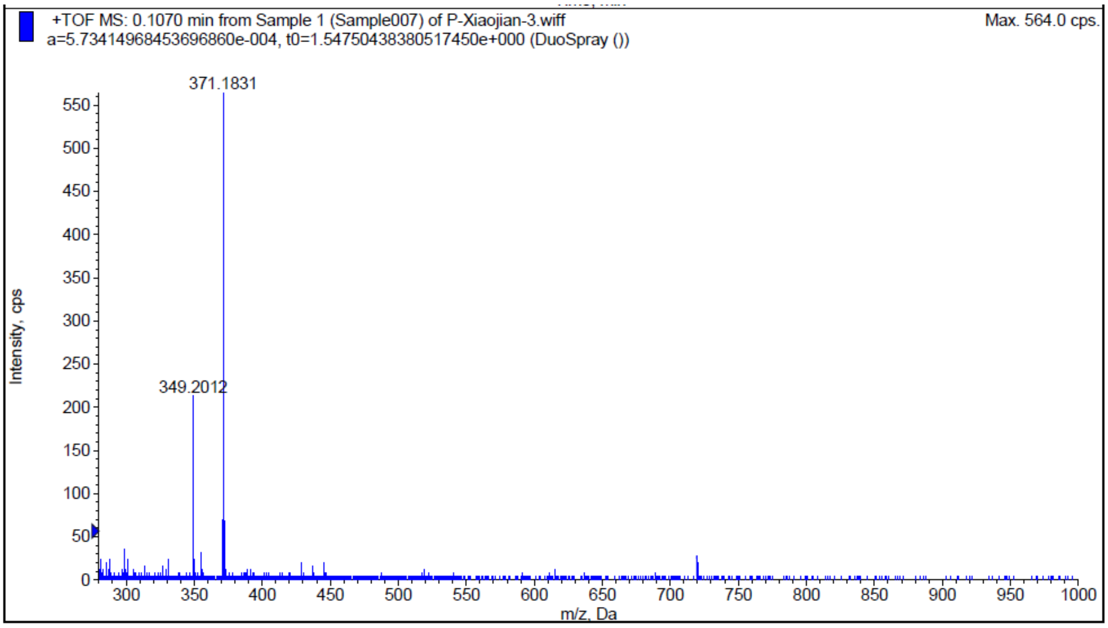

Figure S20. IR and UV spectra of compound **4**.

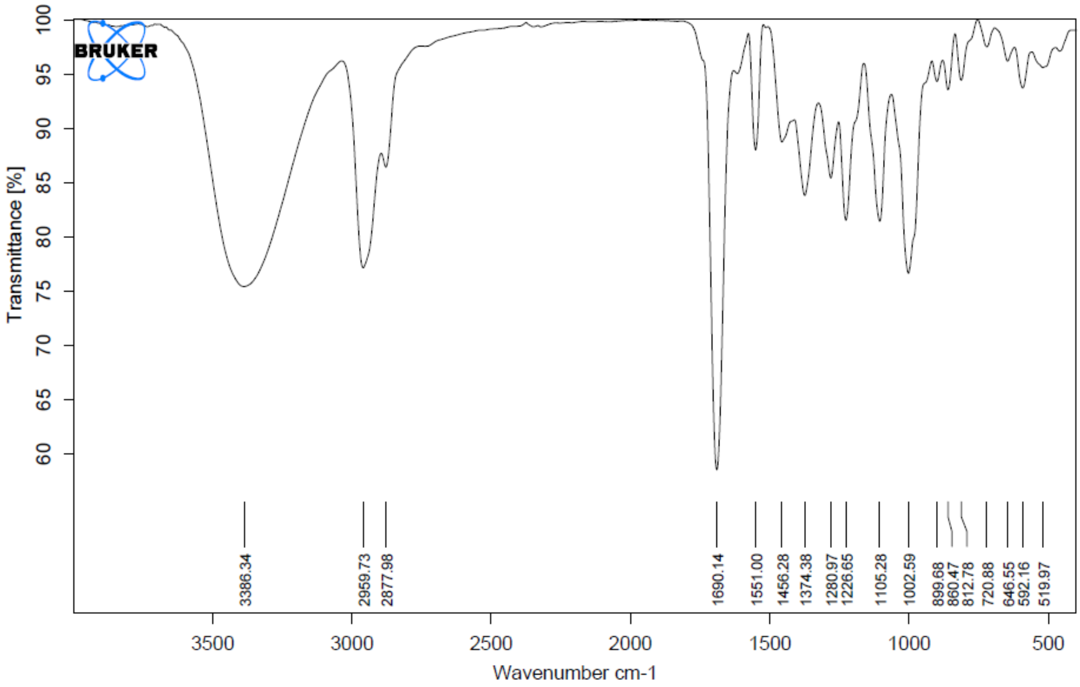

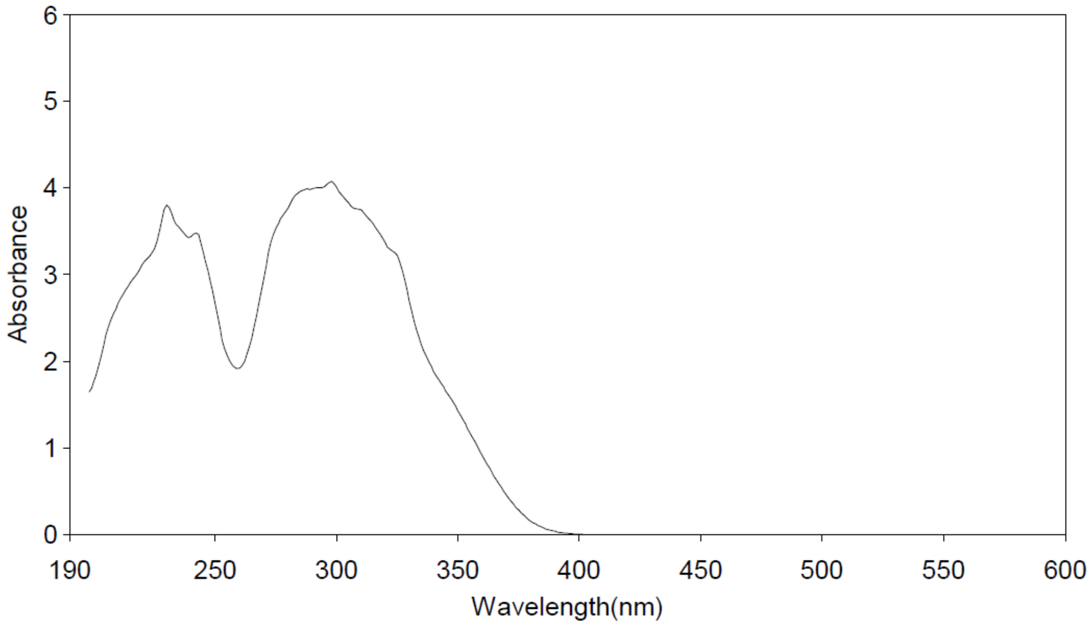

Figure S21. ^{1}H NMR and ^{13}C spectra of compound **5** in CD_3OD.

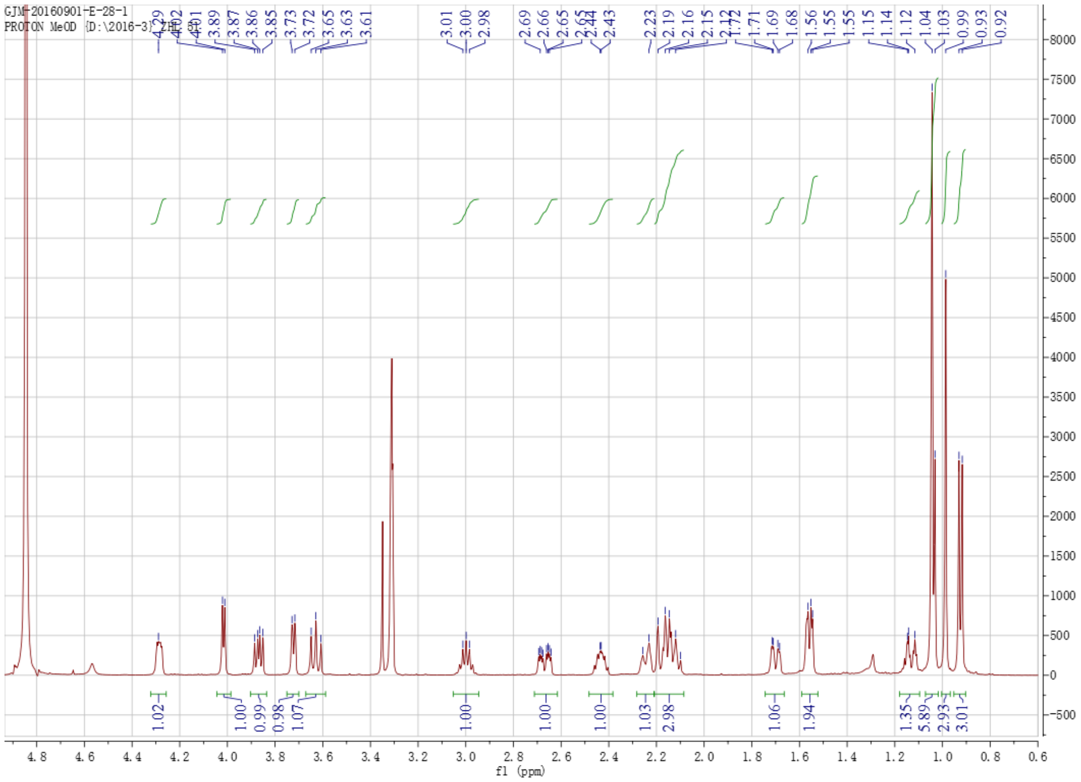

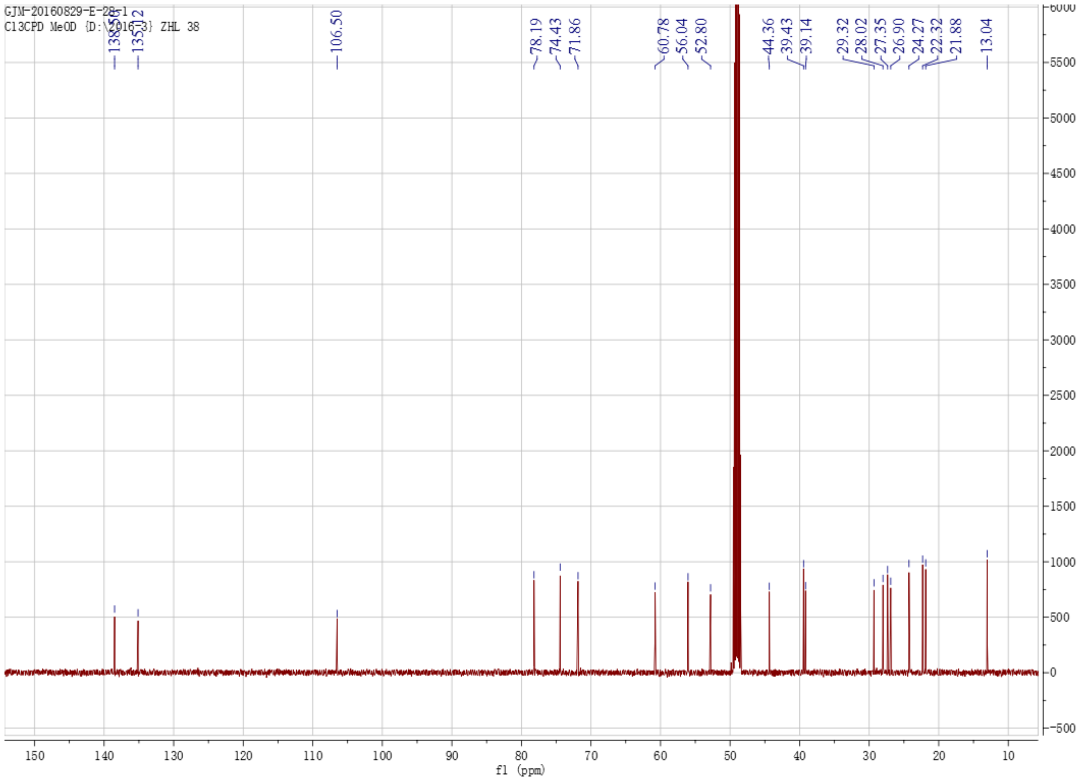

Figure S22. HSQC and ^{1}H-^{1}H COSY spectra of compound **5** in CD_3OD.

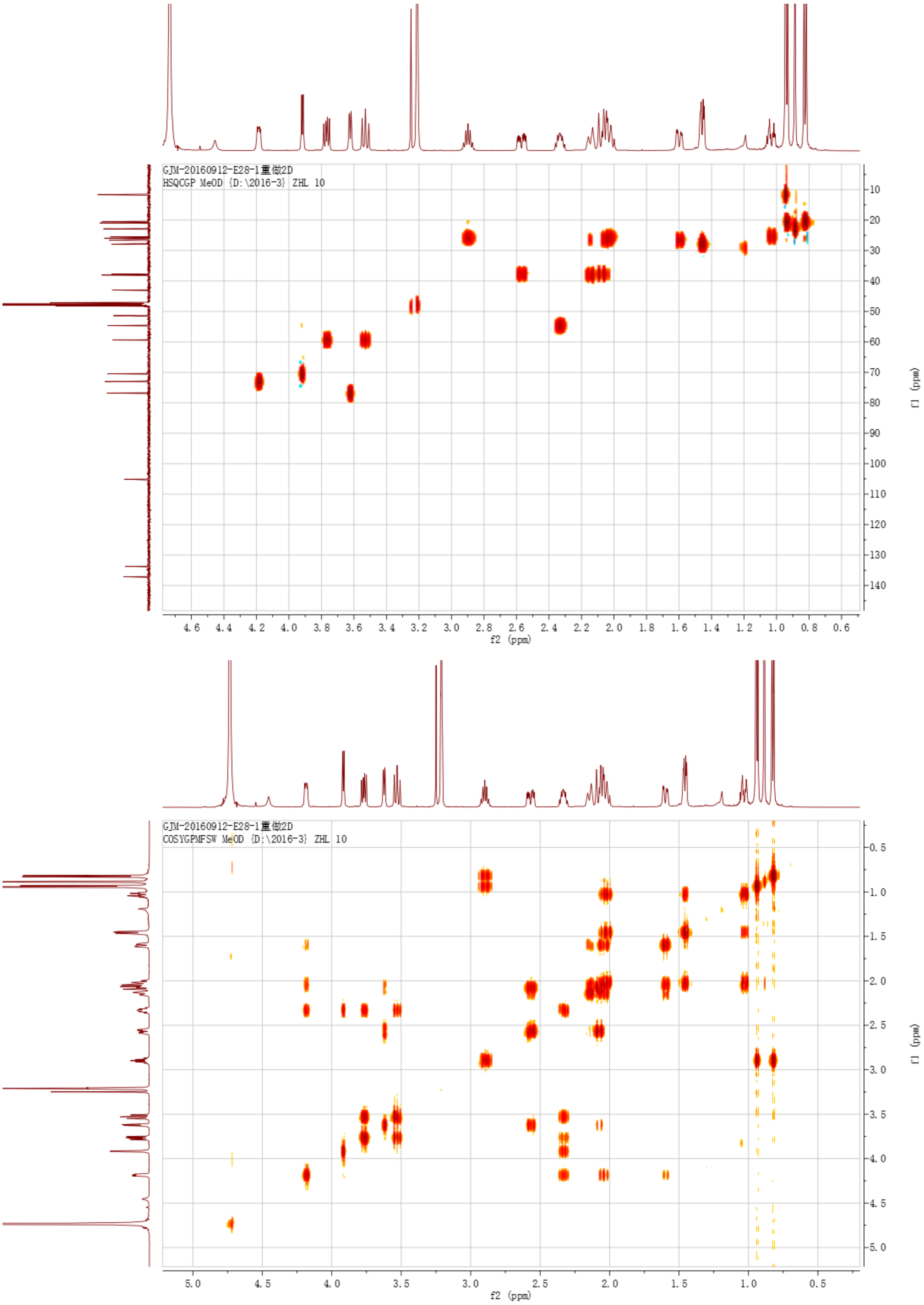

Figure S23. HMBC and NOESY spectra of compound **5** in CD_3OD.

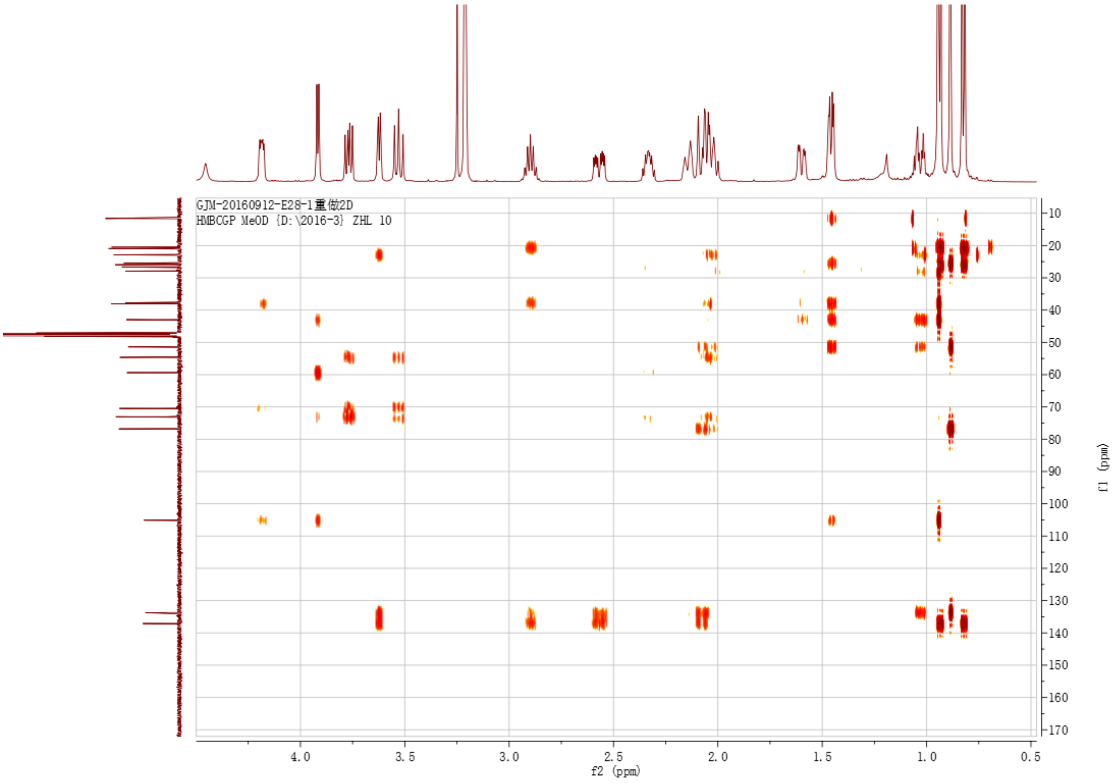

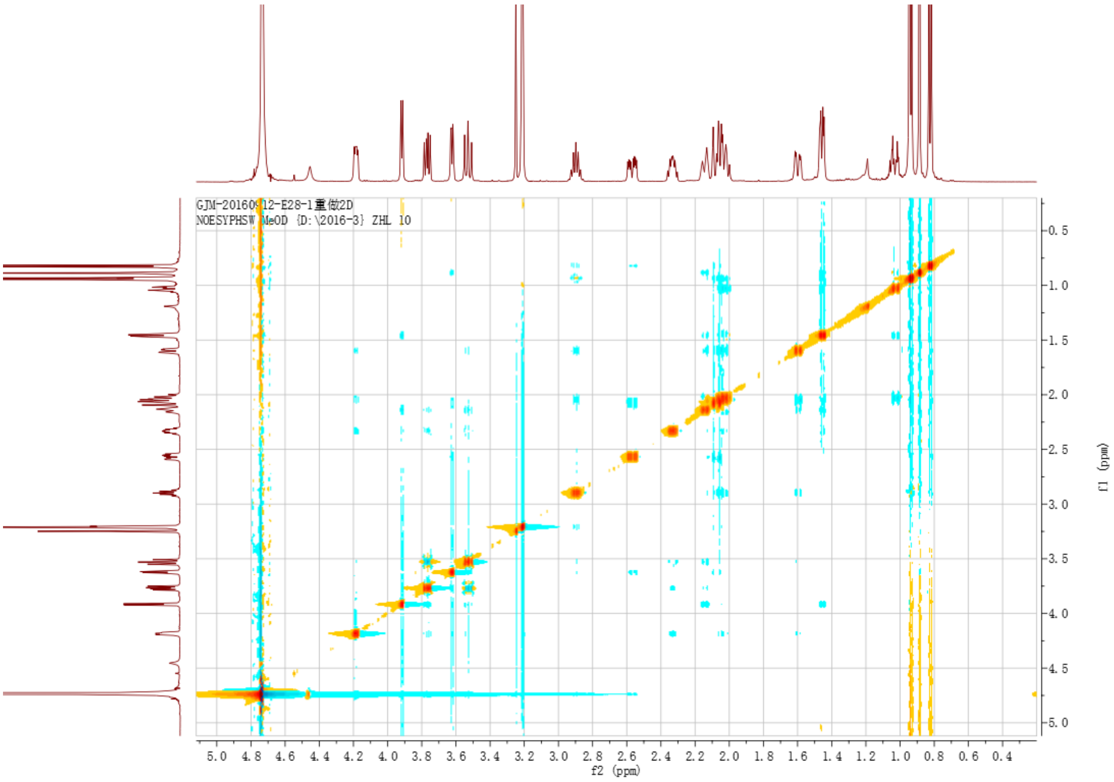

Figure S24. HRESIMS spectrum of compound **5**.

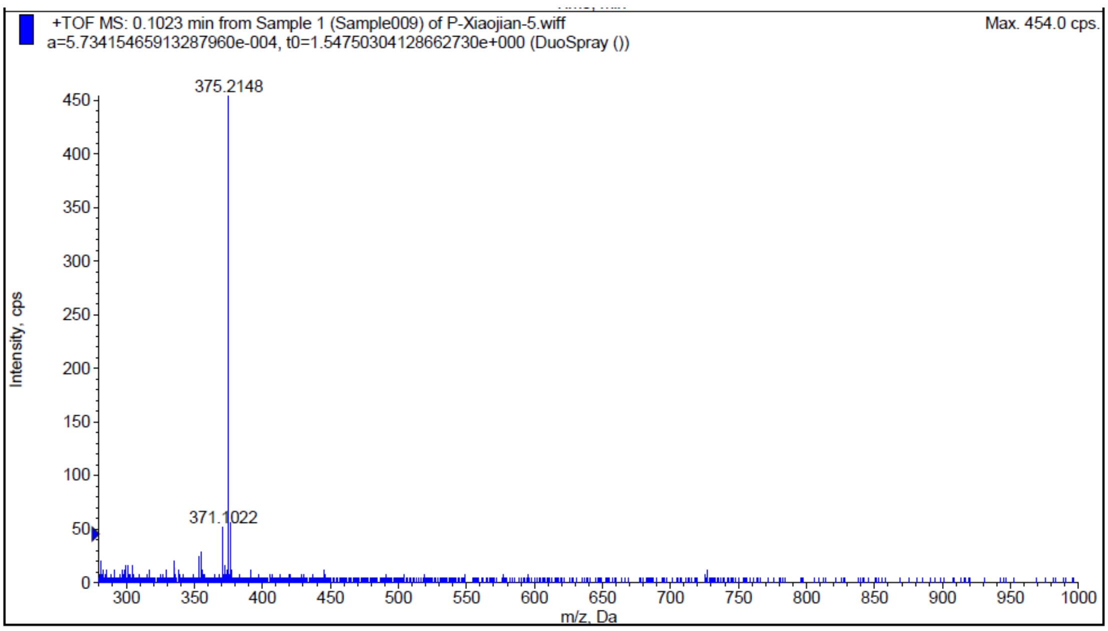

Figure S25. IR and UV spectra of compound **5**.

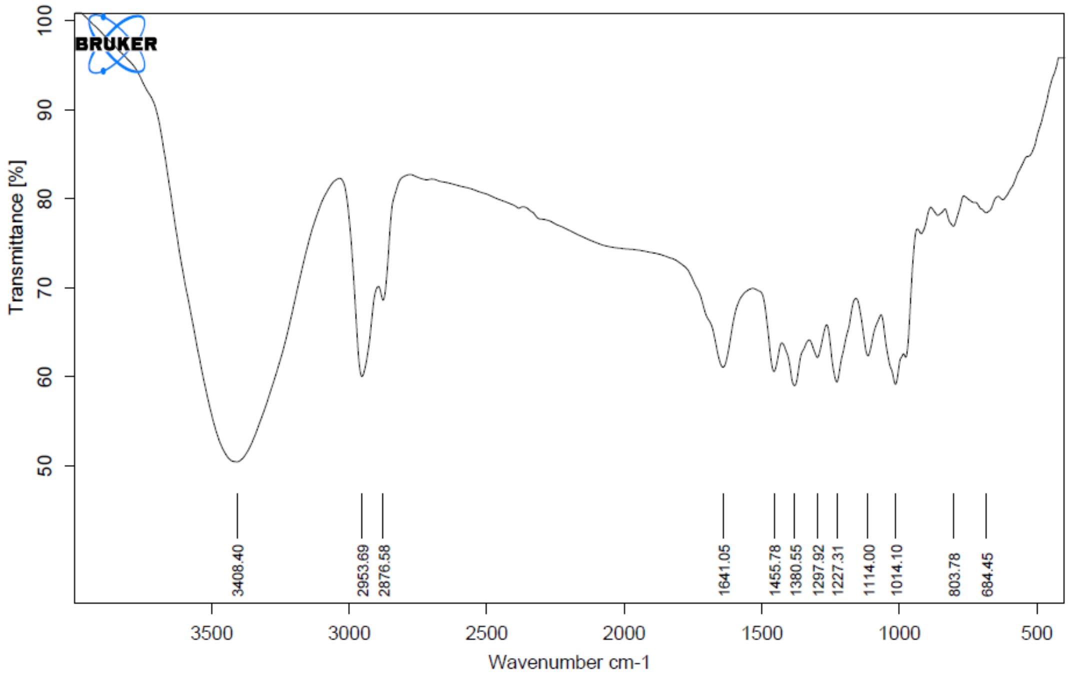

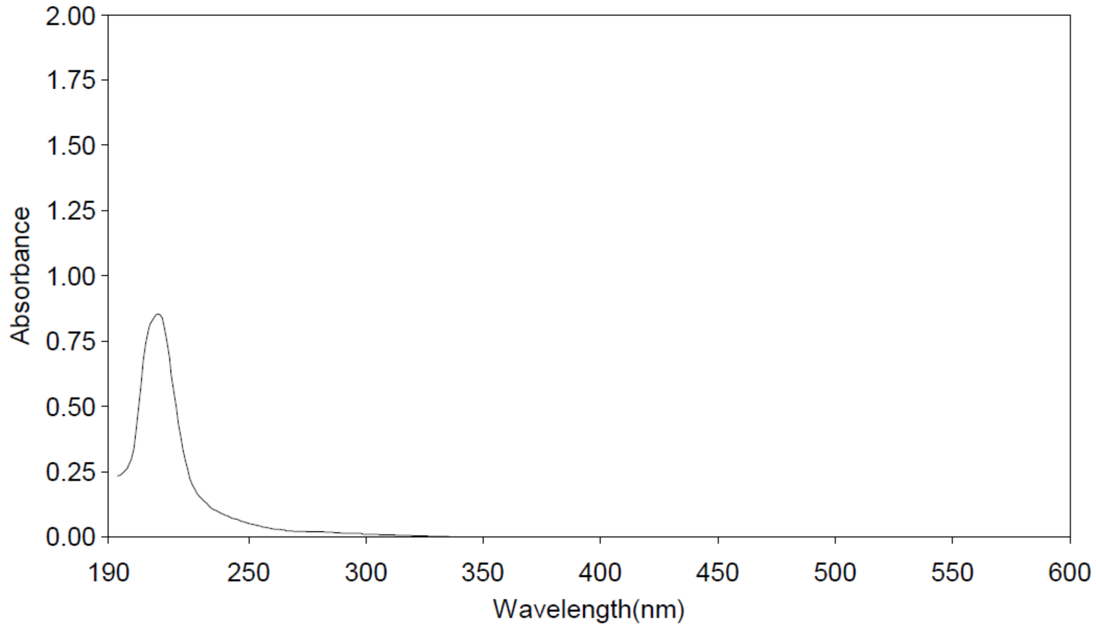

Figure S26. ^{1}H NMR and ^{13}C spectra of compound **6** in CD_3OD.

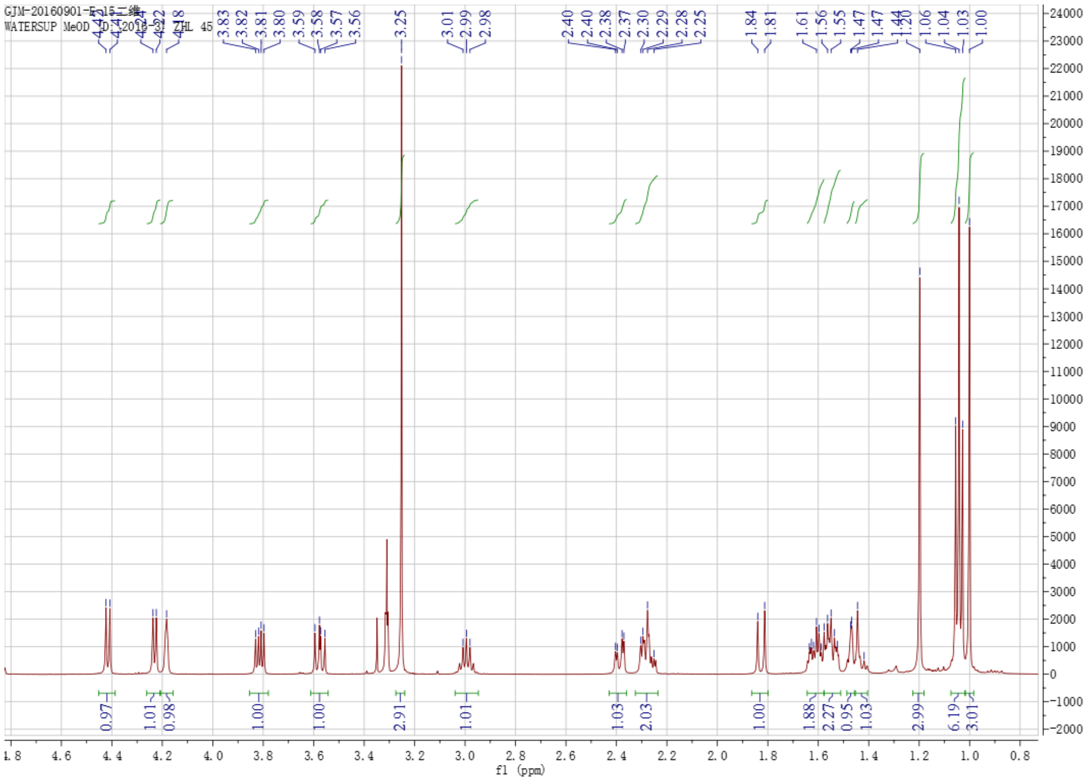

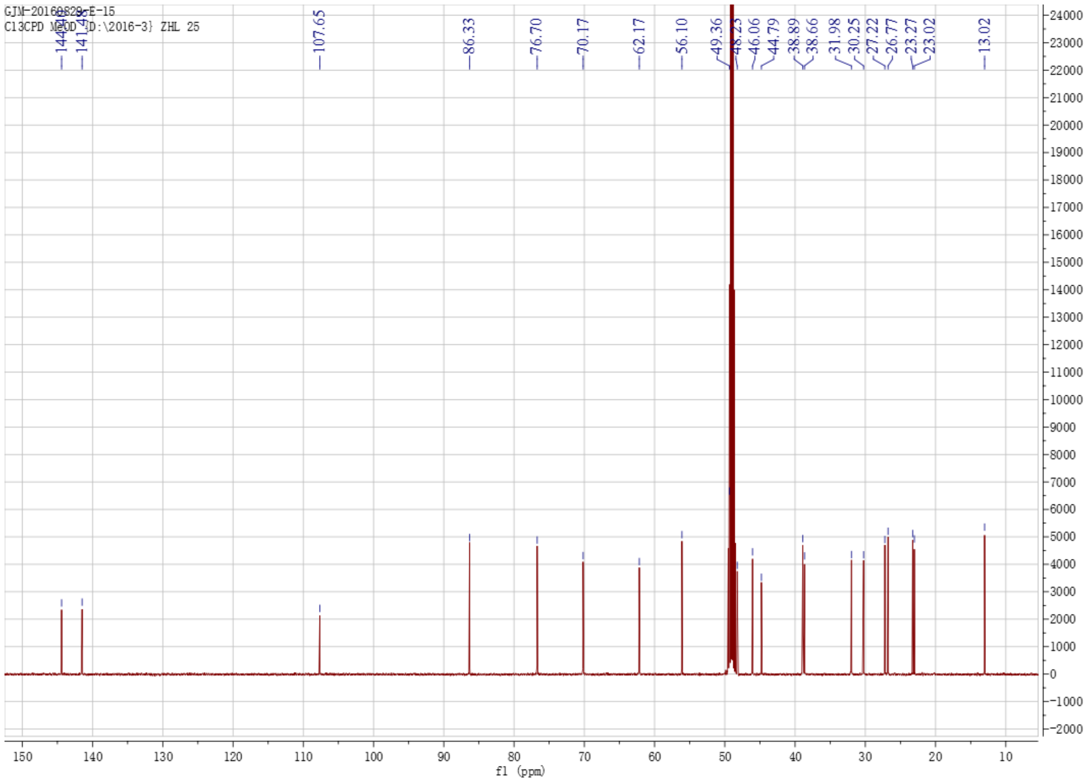

Figure S27. HSQC and ^{1}H-^{1}H COSY spectra of compound **6** in CD_3OD.

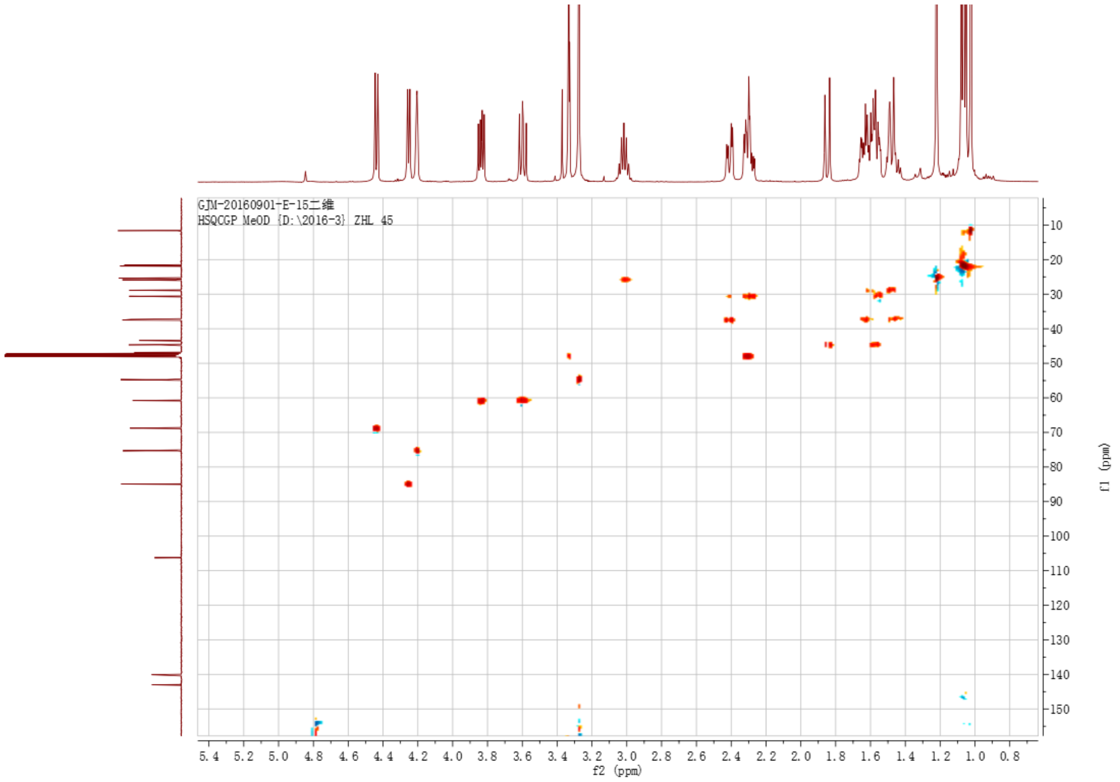

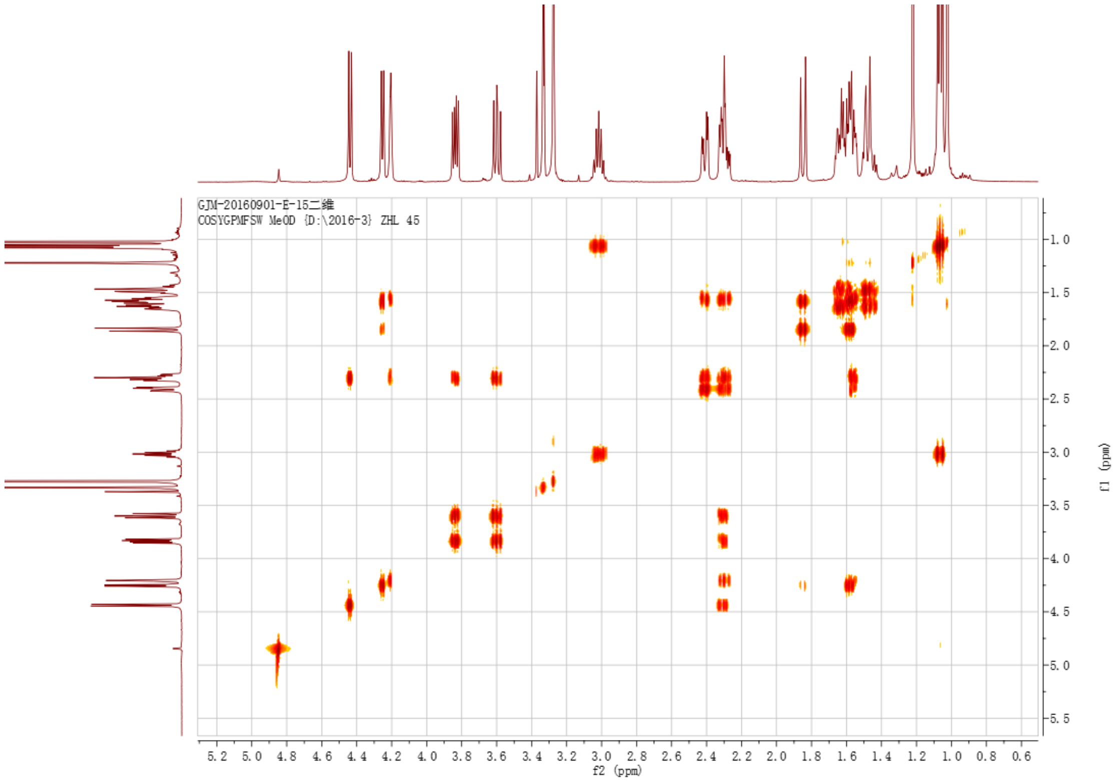

Figure S28. HMBC and NOESY spectra of compound **6** in CD_3OD.

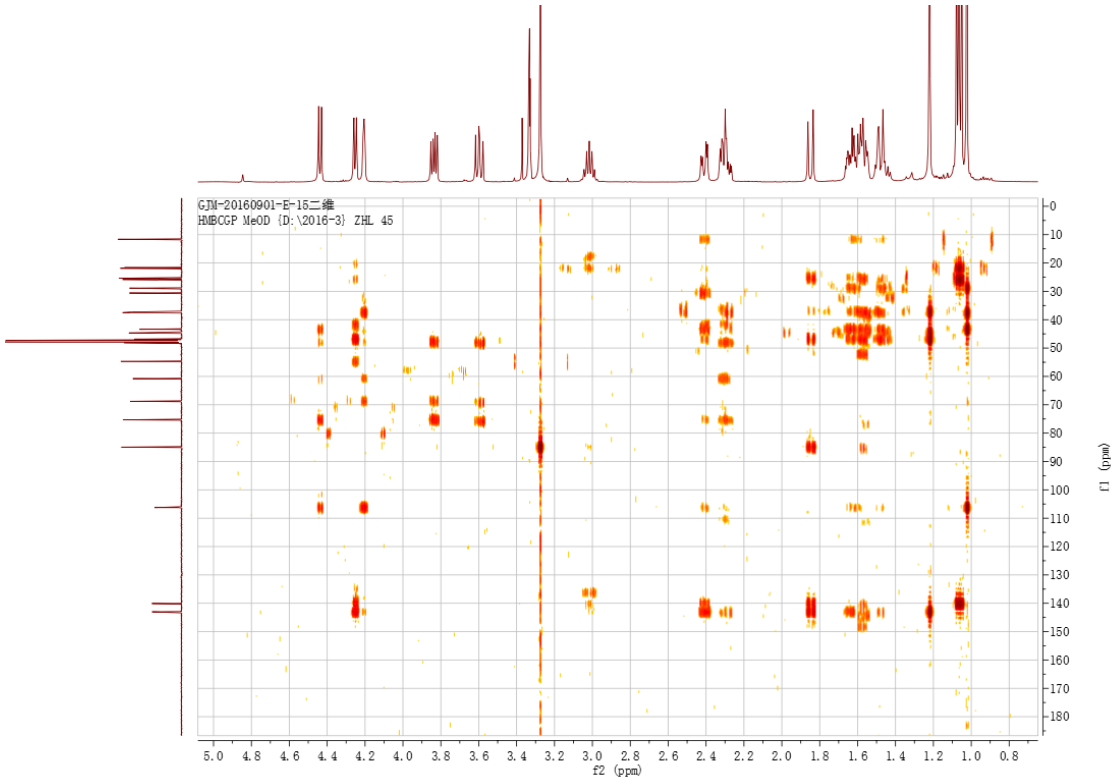

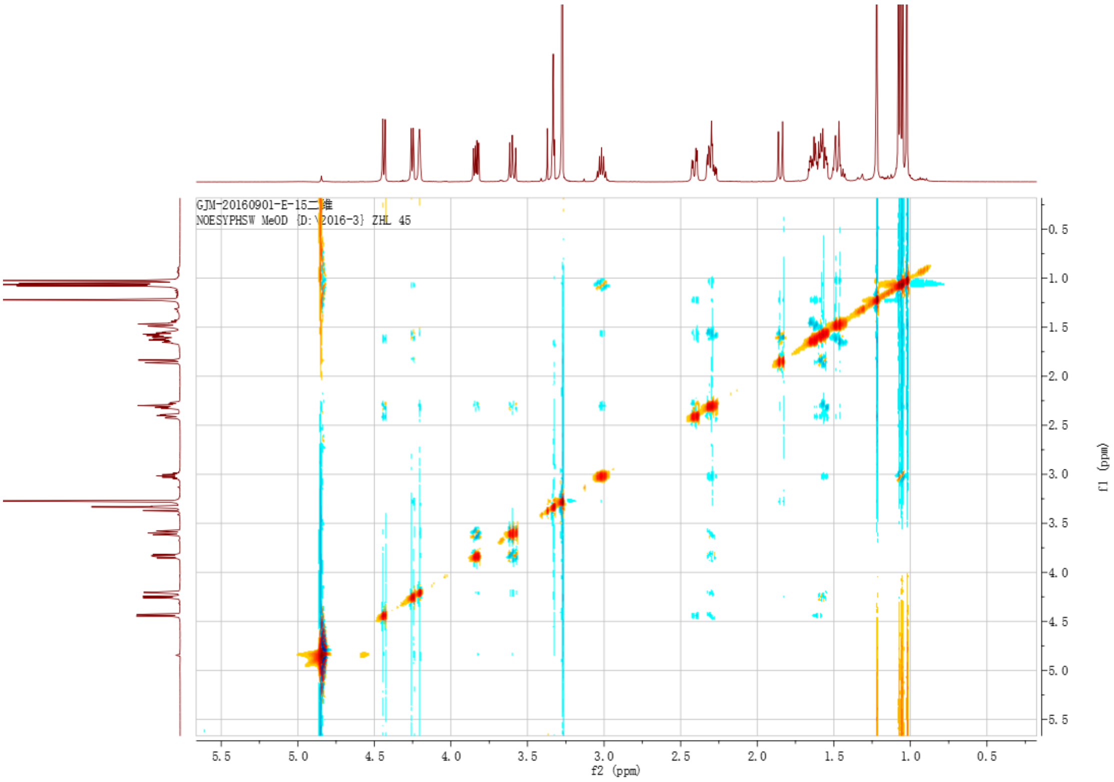

Figure S29. HRESIMS spectrum of compound **6**.

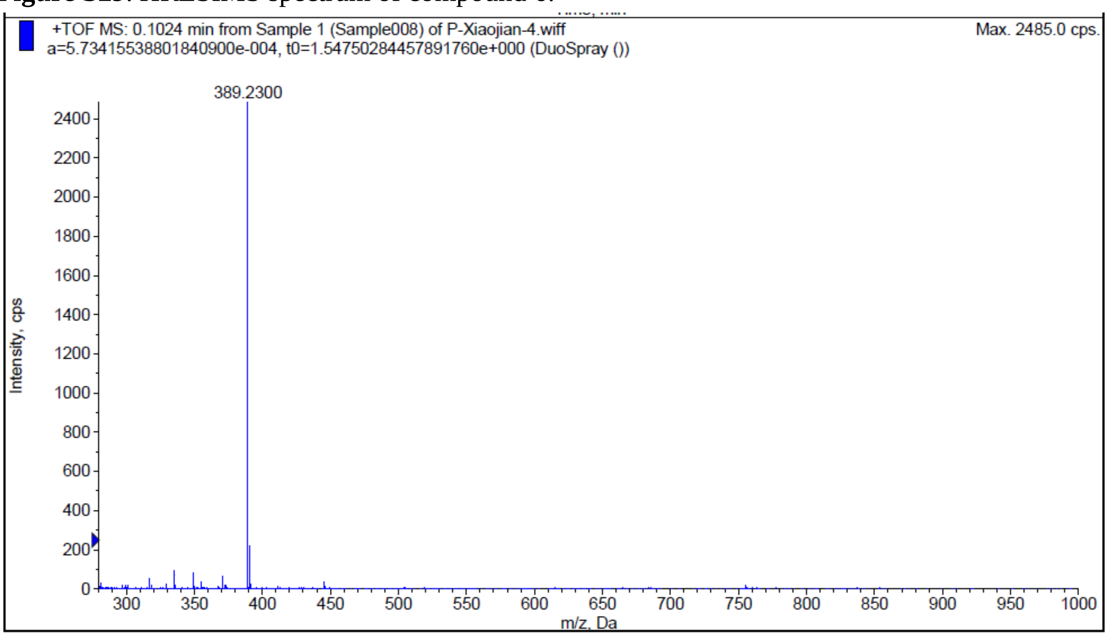

Figure S30. IR and UV spectra of compound **6**.

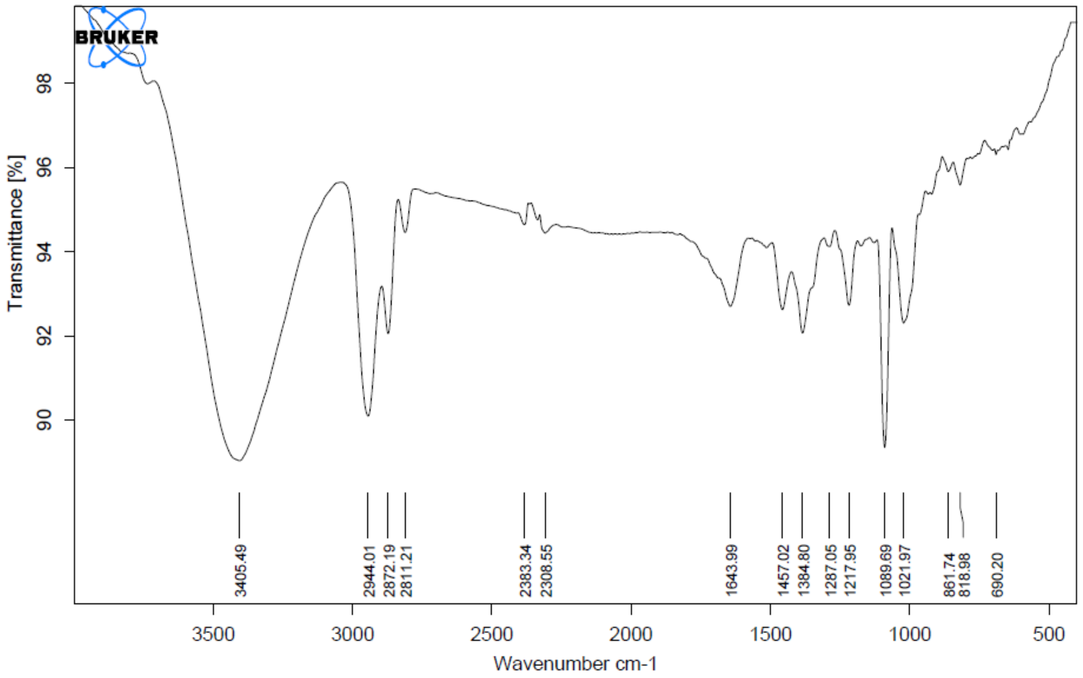

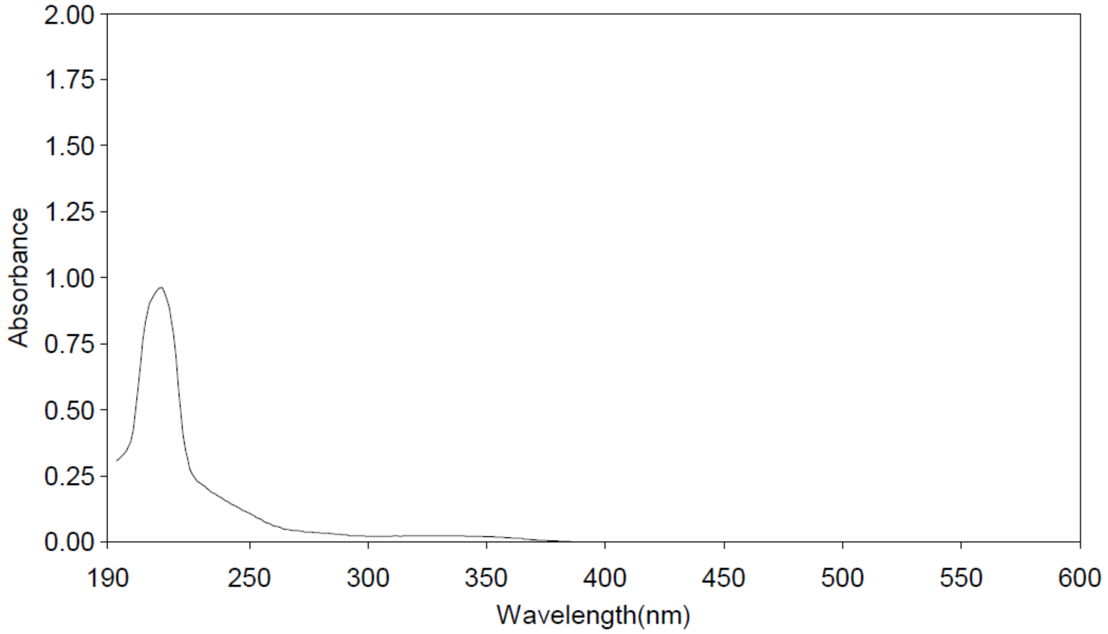

Figure S31. ^{1}H NMR and ^{13}C spectra of compound **7** in CD_3OD.

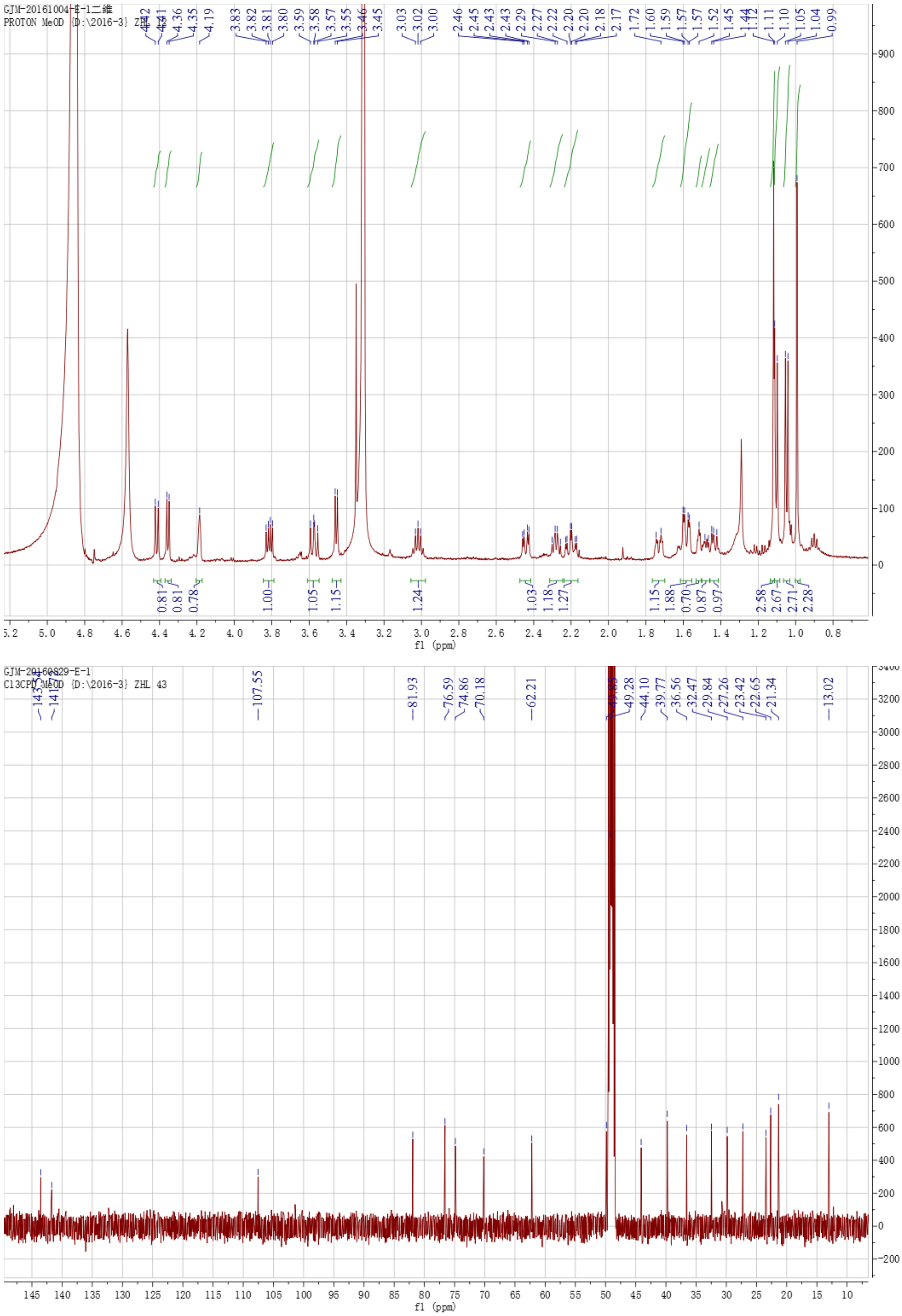

Figure S32. HSQC and ^{1}H-^{1}H COSY spectra of compound **7** in CD_3OD.

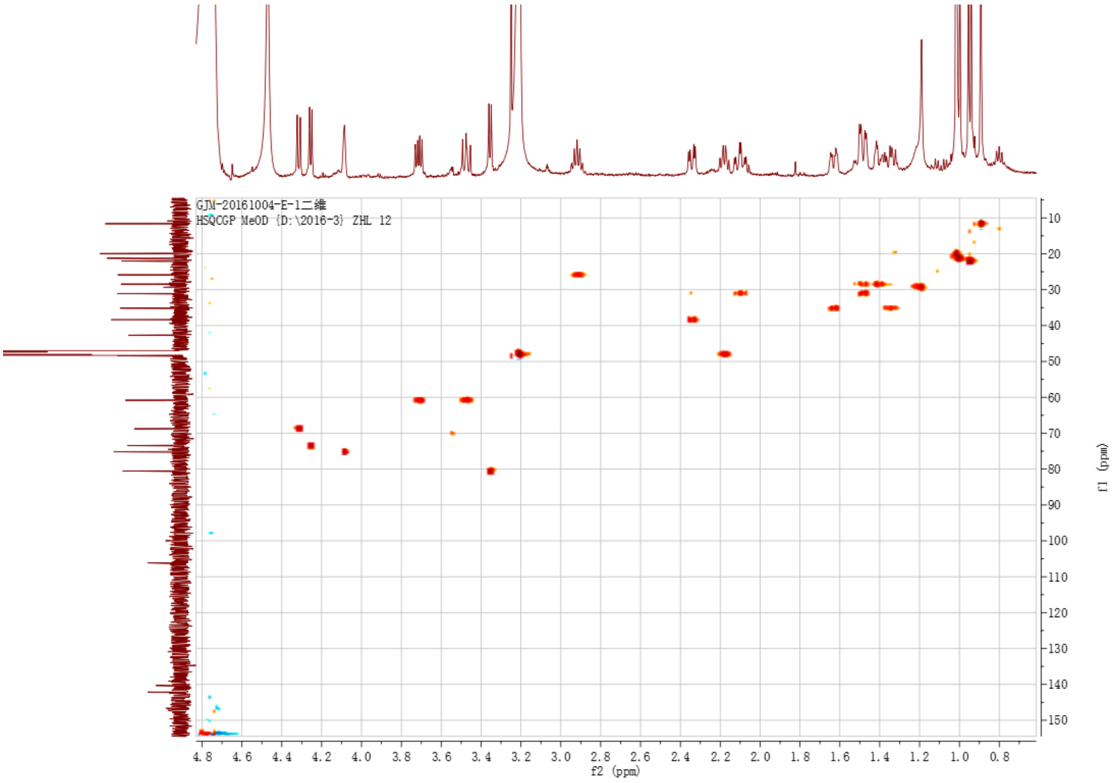

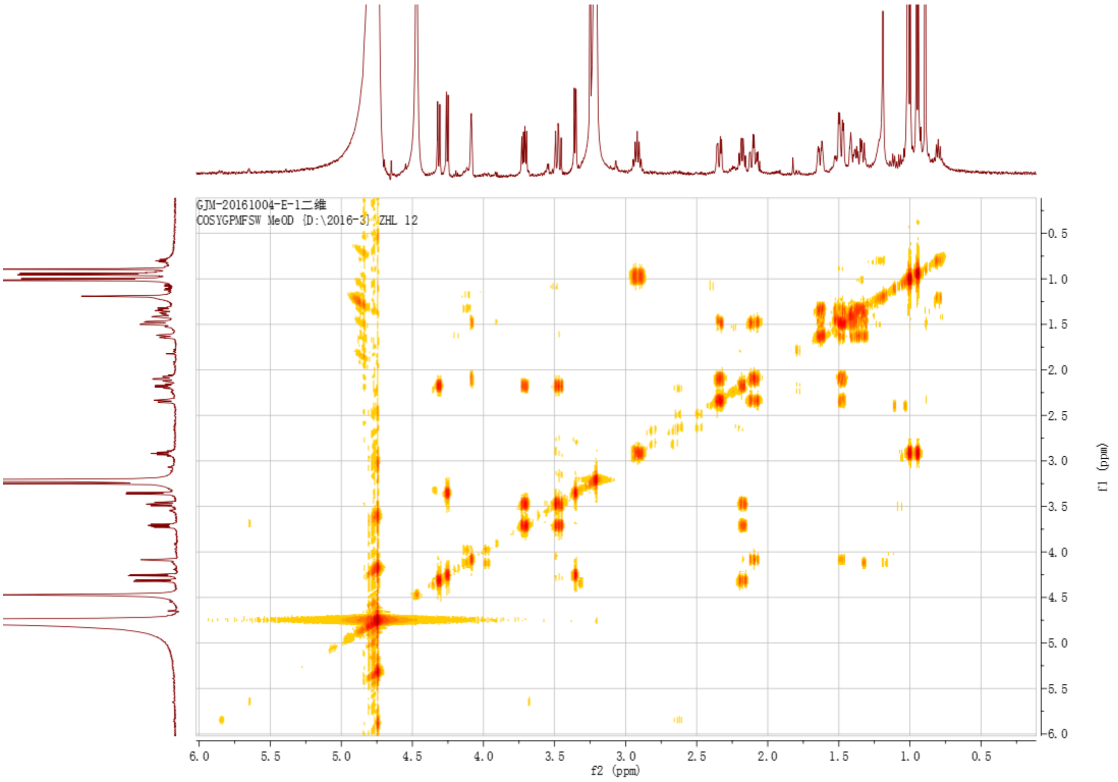

Figure S33. HMBC and NOESY spectra of compound **7** in CD_3OD.

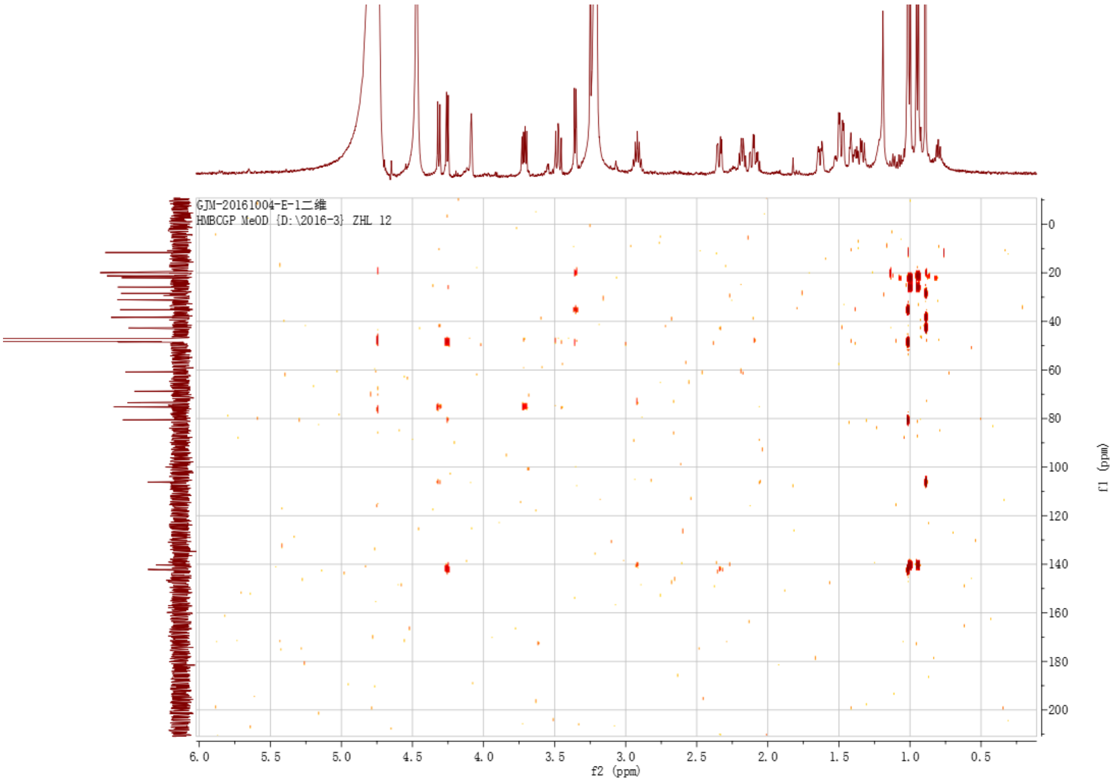

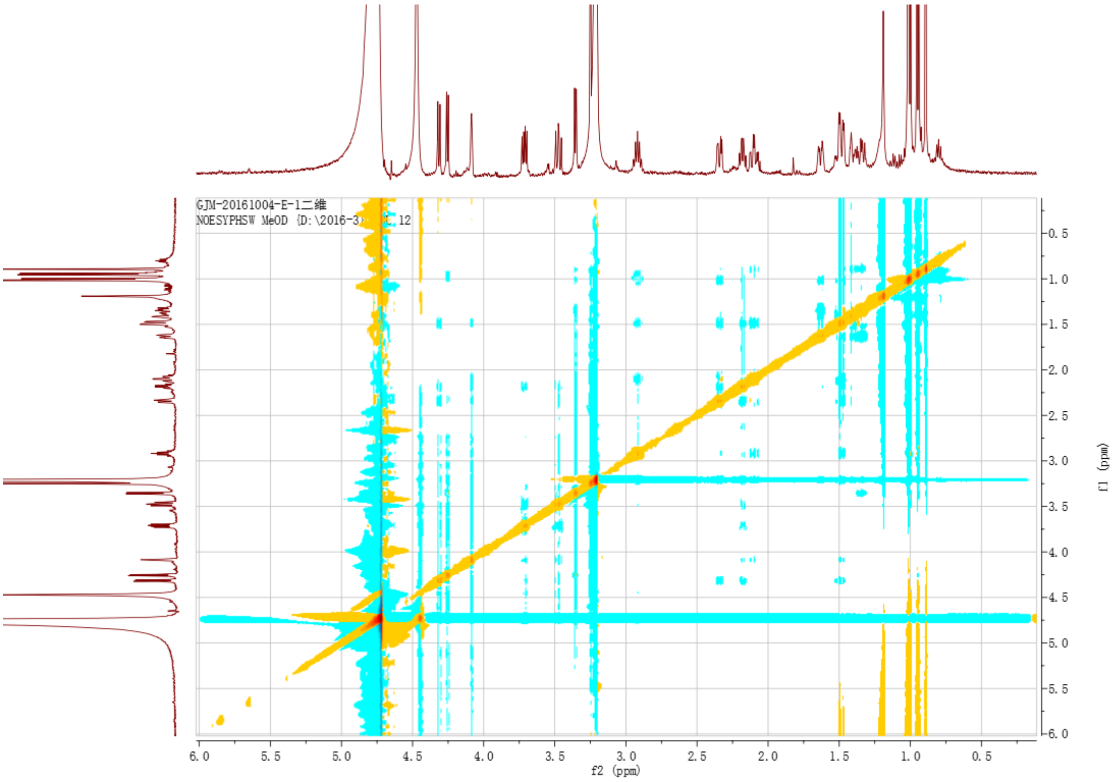

Figure S34. HRESIMS spectrum of compound **7**.

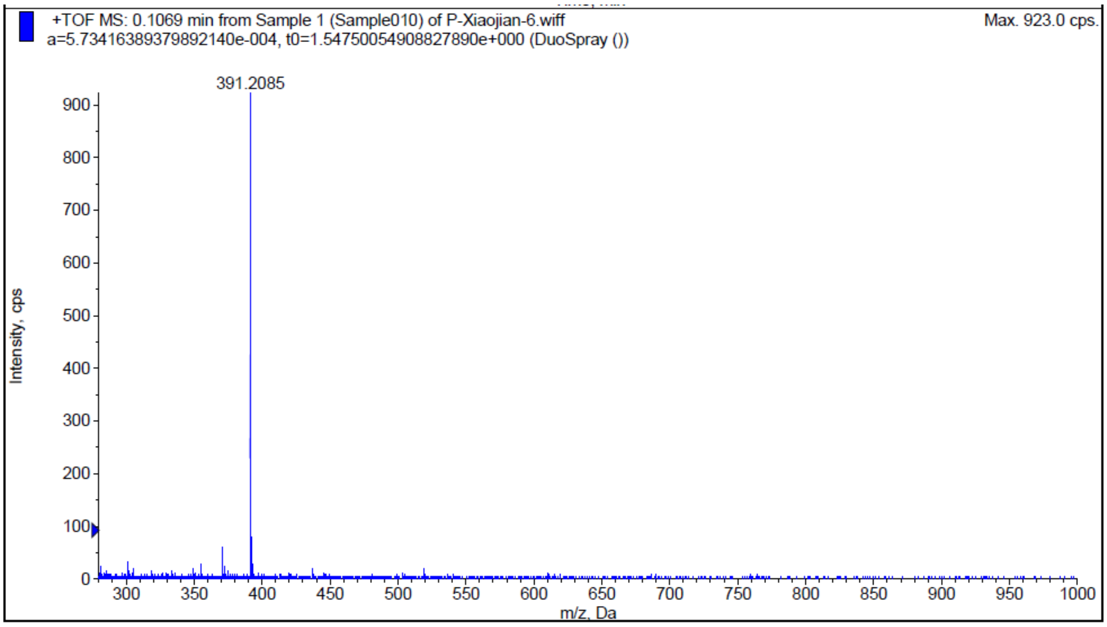

Figure S35. IR and UV spectra of compound**7**.

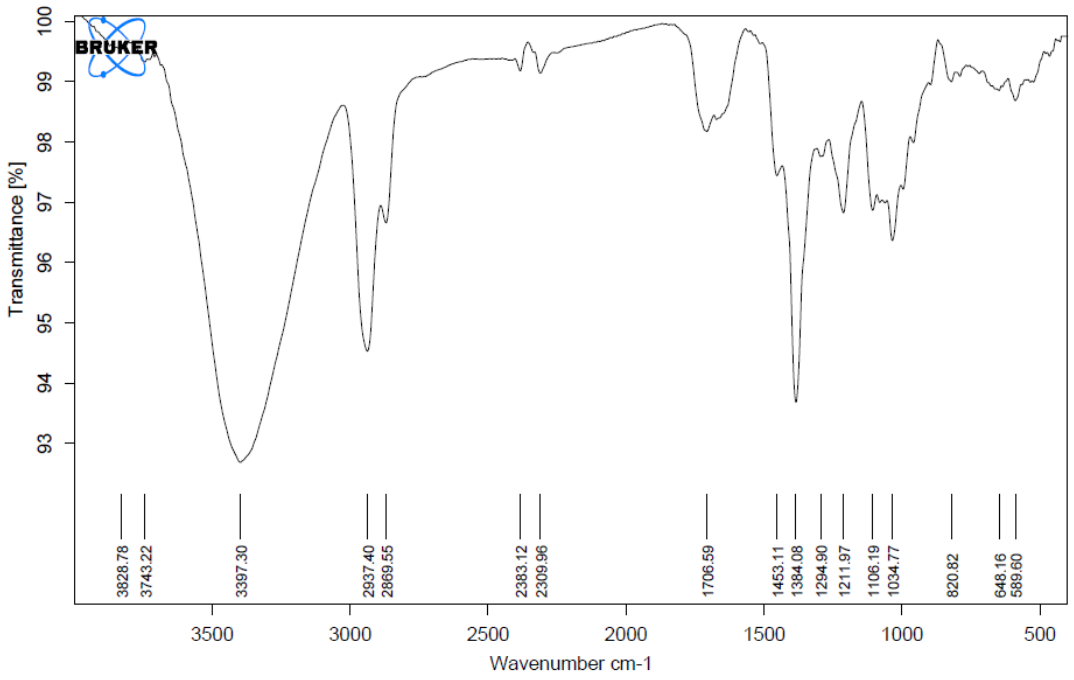

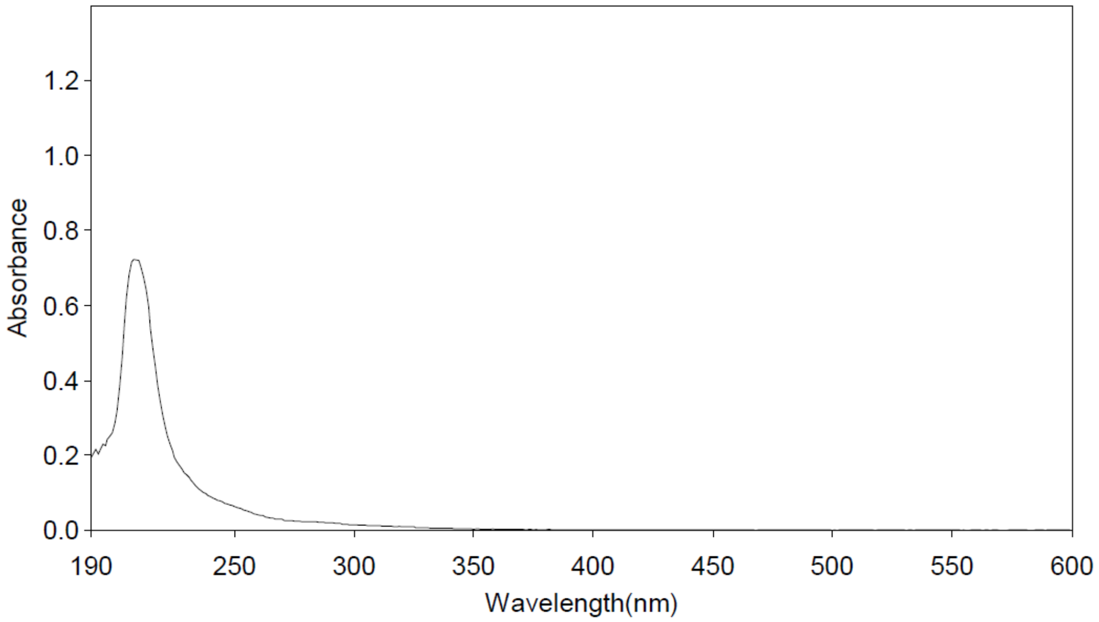

Figure S36. ^{1}H NMR and ^{13}C spectra of compound **8** in CD_3OD.

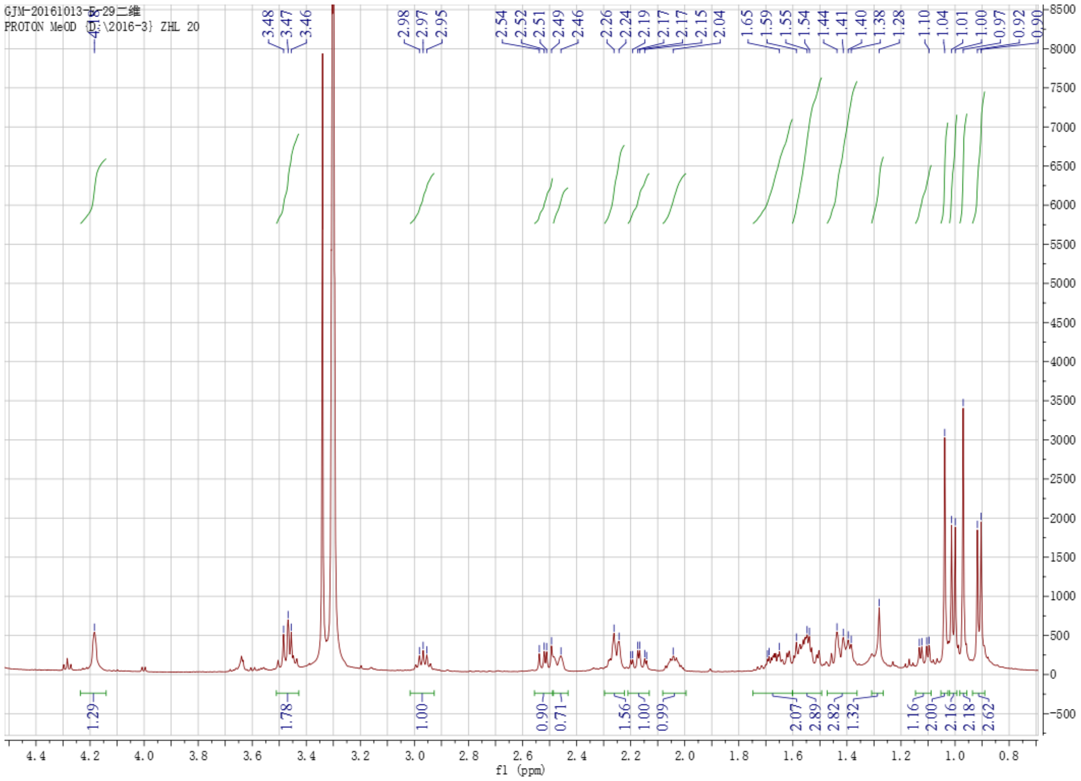

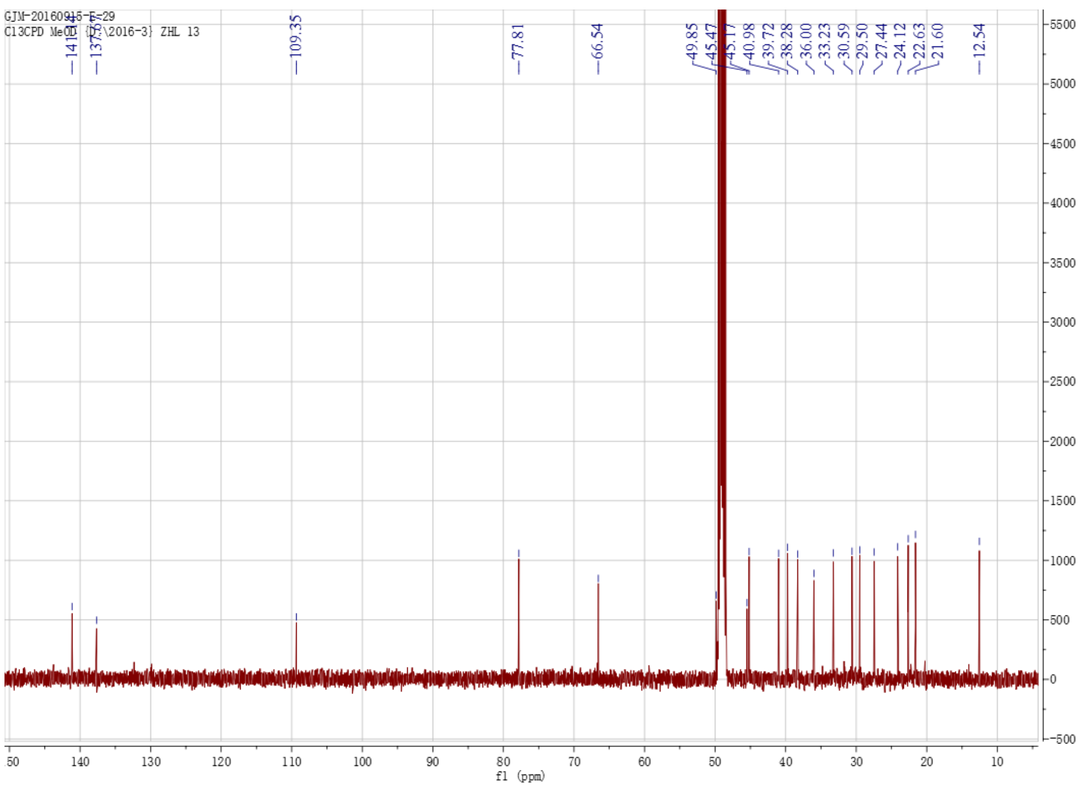

Figure S37. HSQC and ^{1}H-^{1}H COSY spectra of compound **8** in CD_3OD.

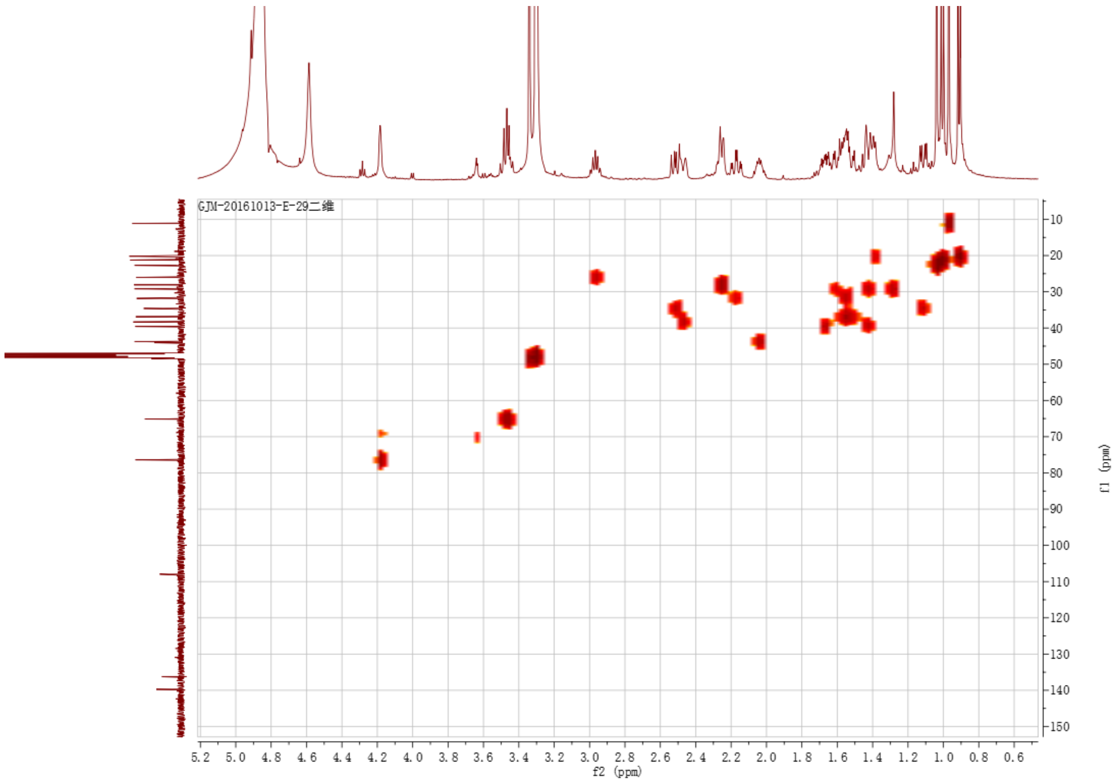

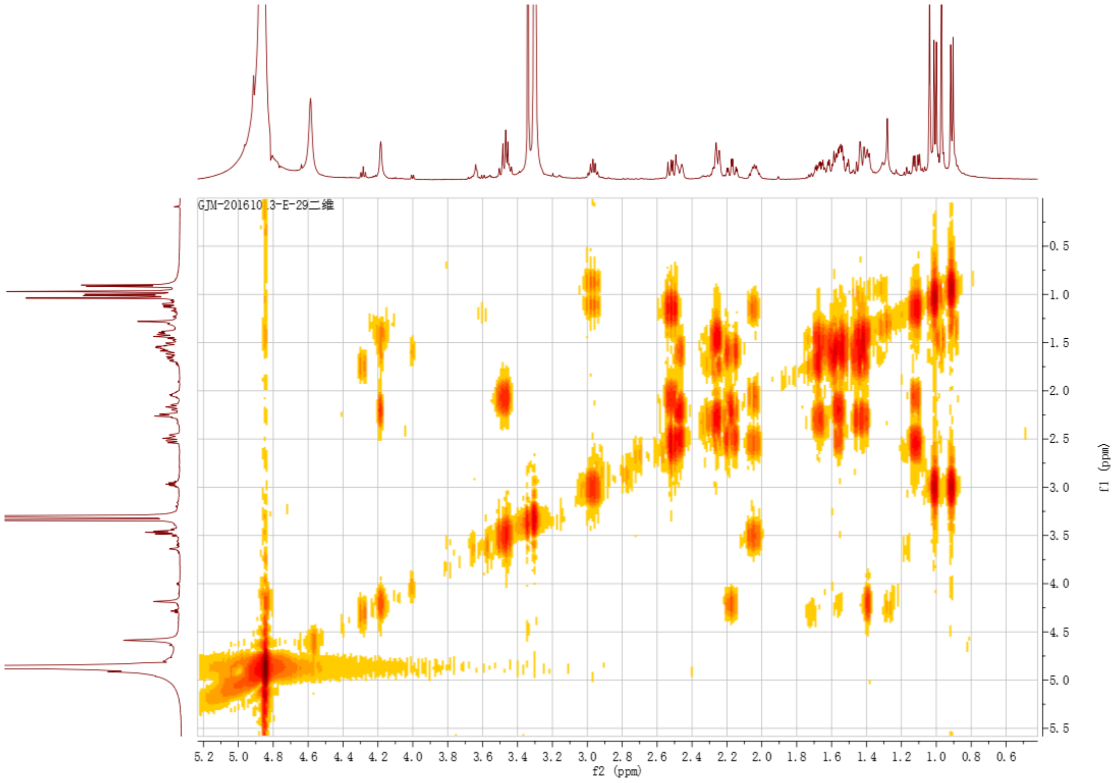

Figure S38. HMBC and NOESY spectra of compound **8** in CD_3OD.

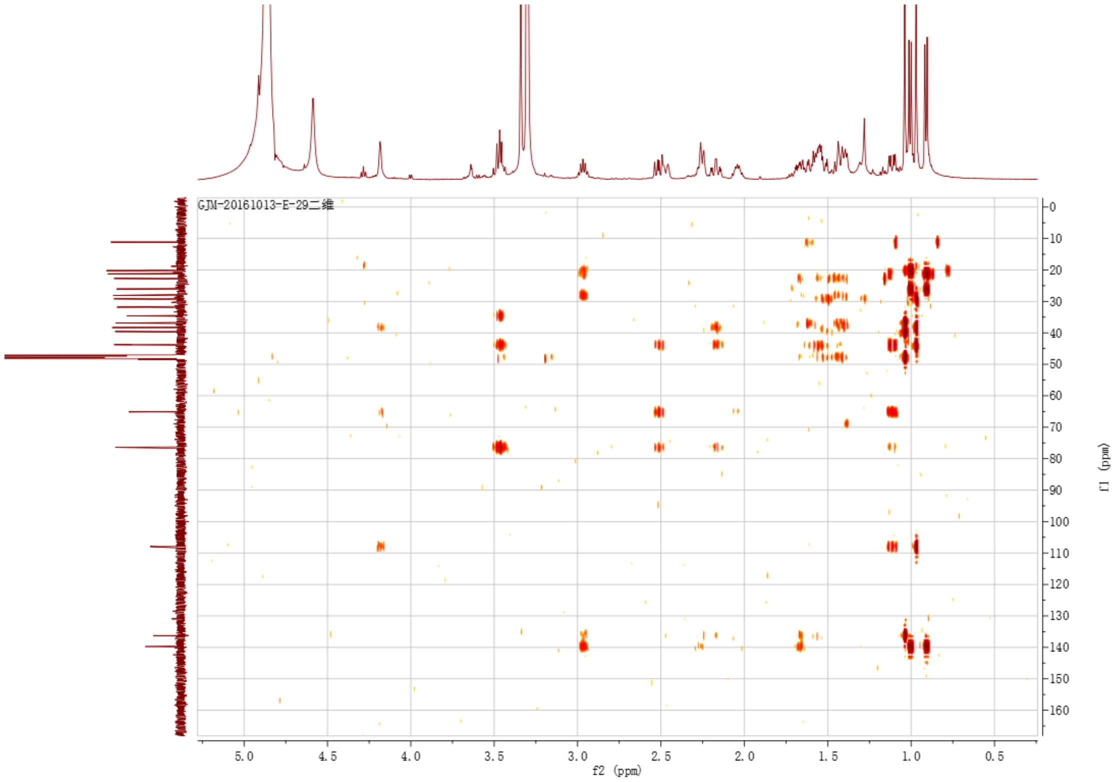

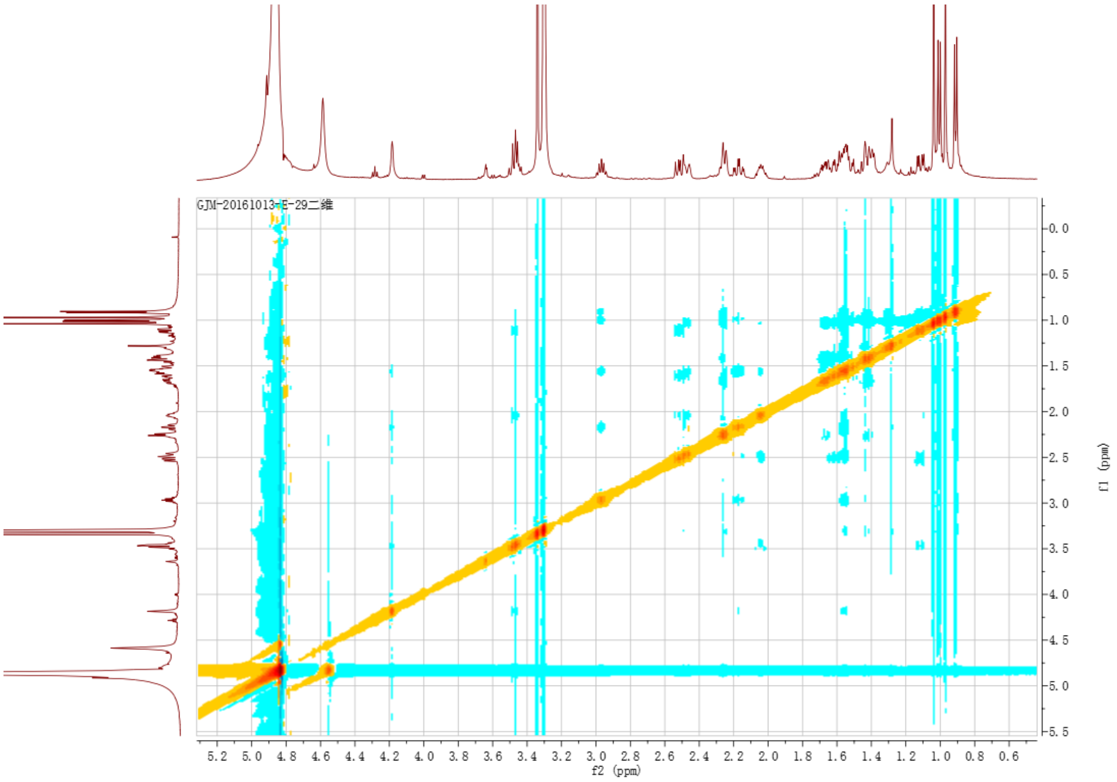

Figure S39. HRESIMS spectrum of compound **8**.

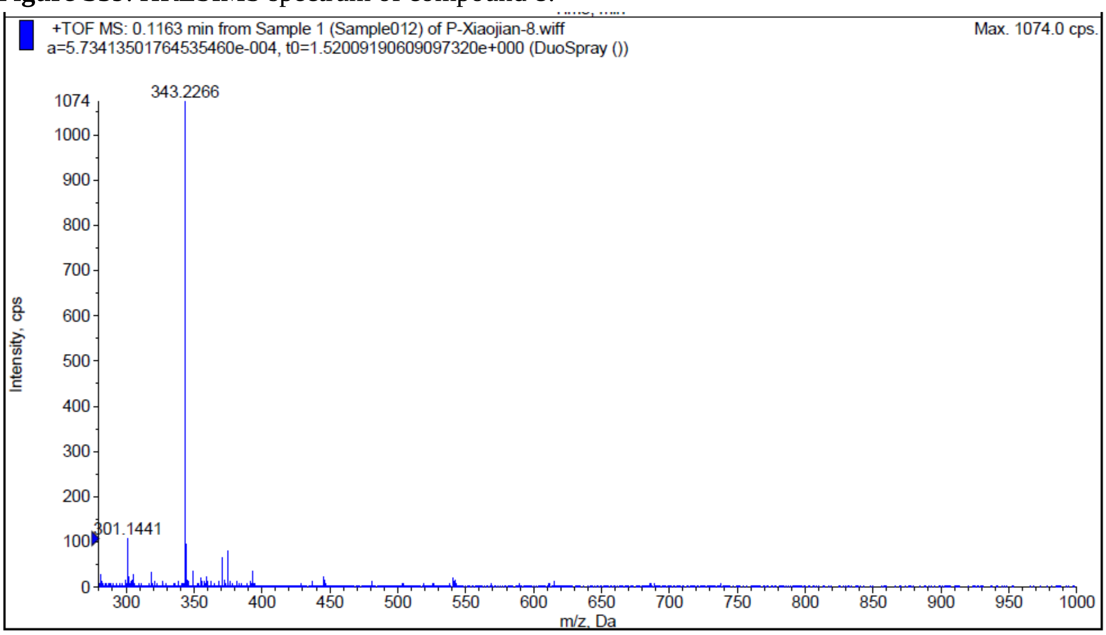

Figure S40. IR and UV spectra of compound **8**.

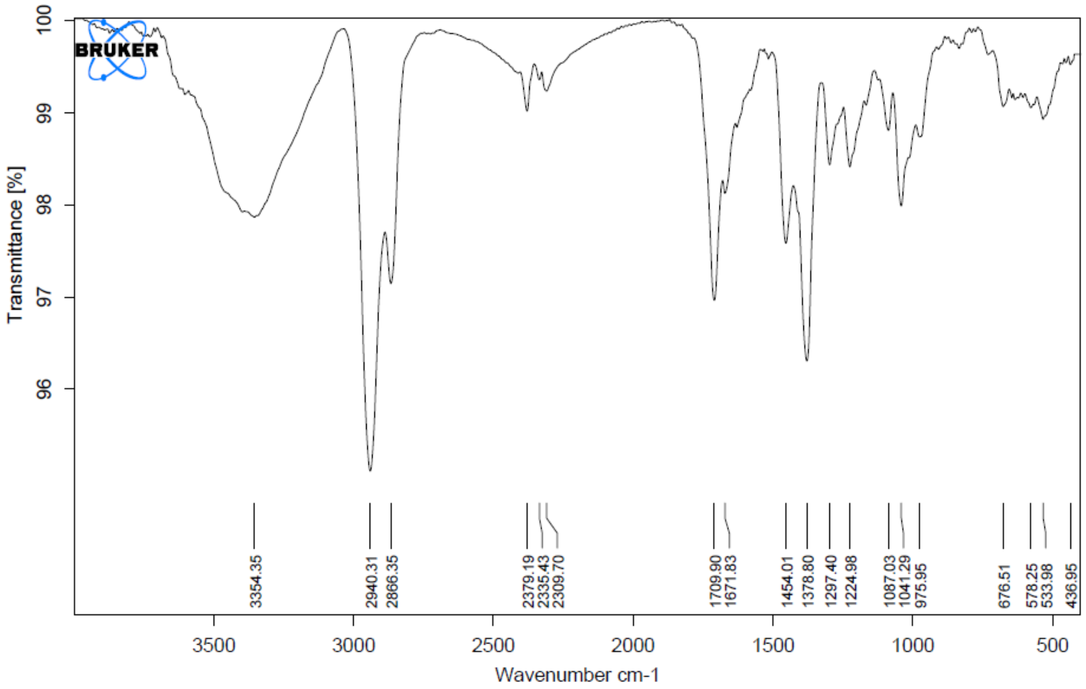

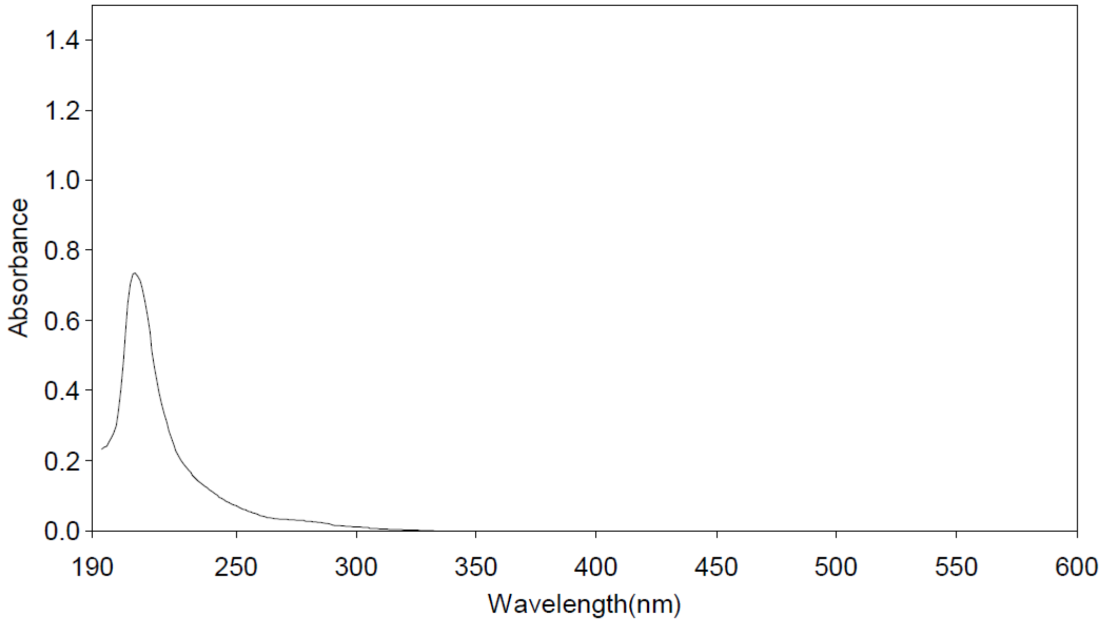

Figure S41. ^{1}H NMR and ^{13}C spectra of compound **9** in CD_3OD.

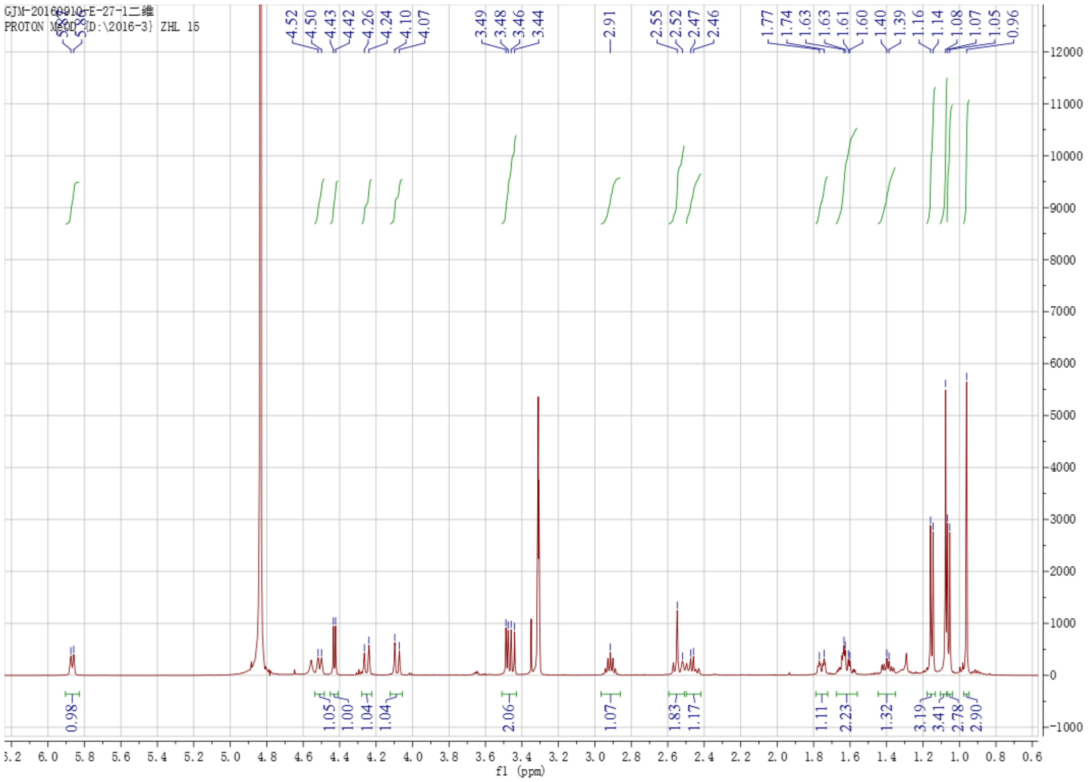

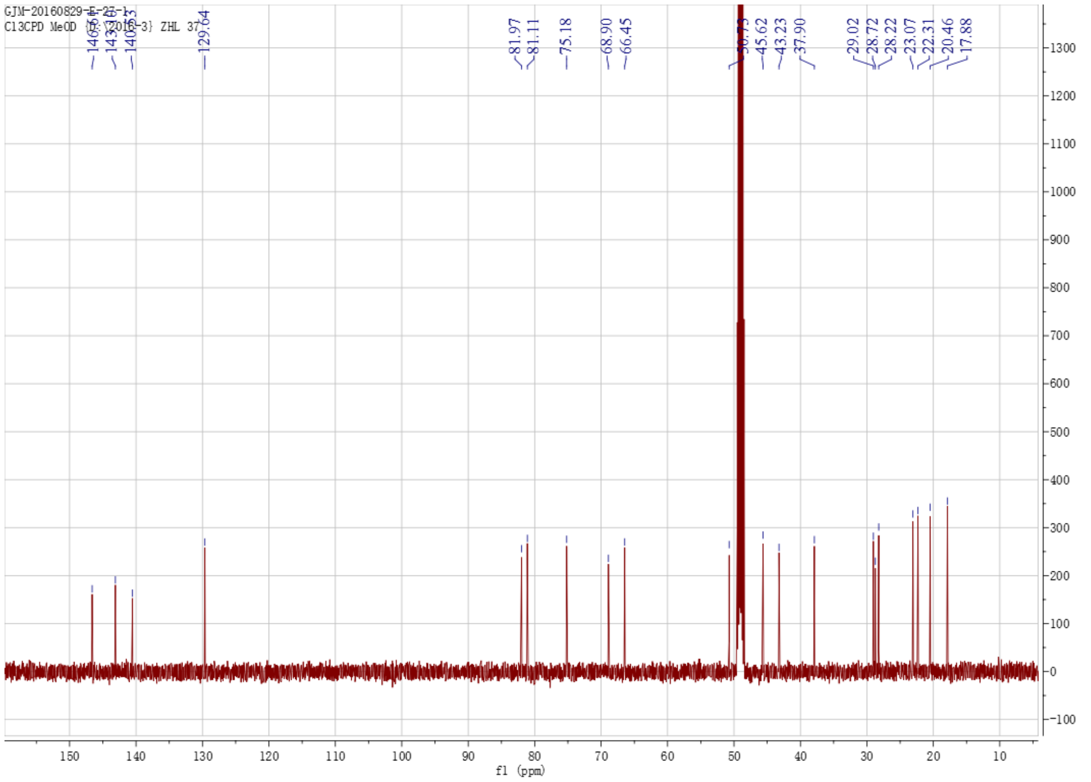

Figure S42. HSQC and ^{1}H-^{1}H COSY spectra of compound **9** in CD_3OD.

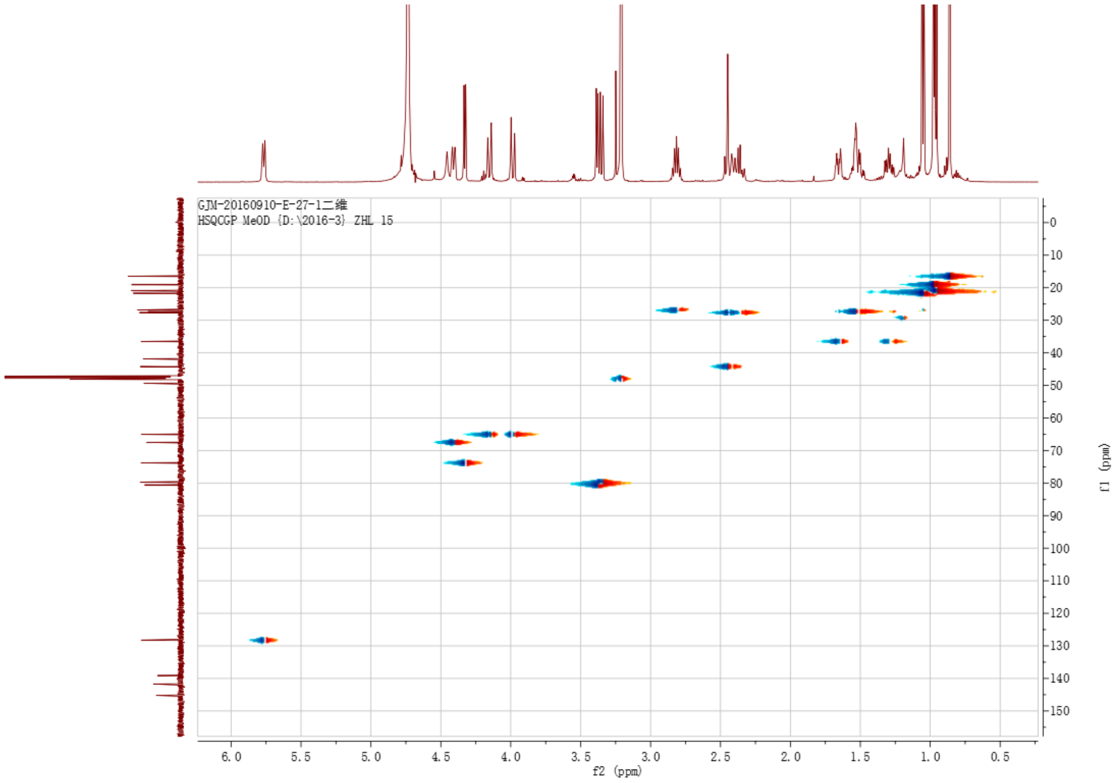

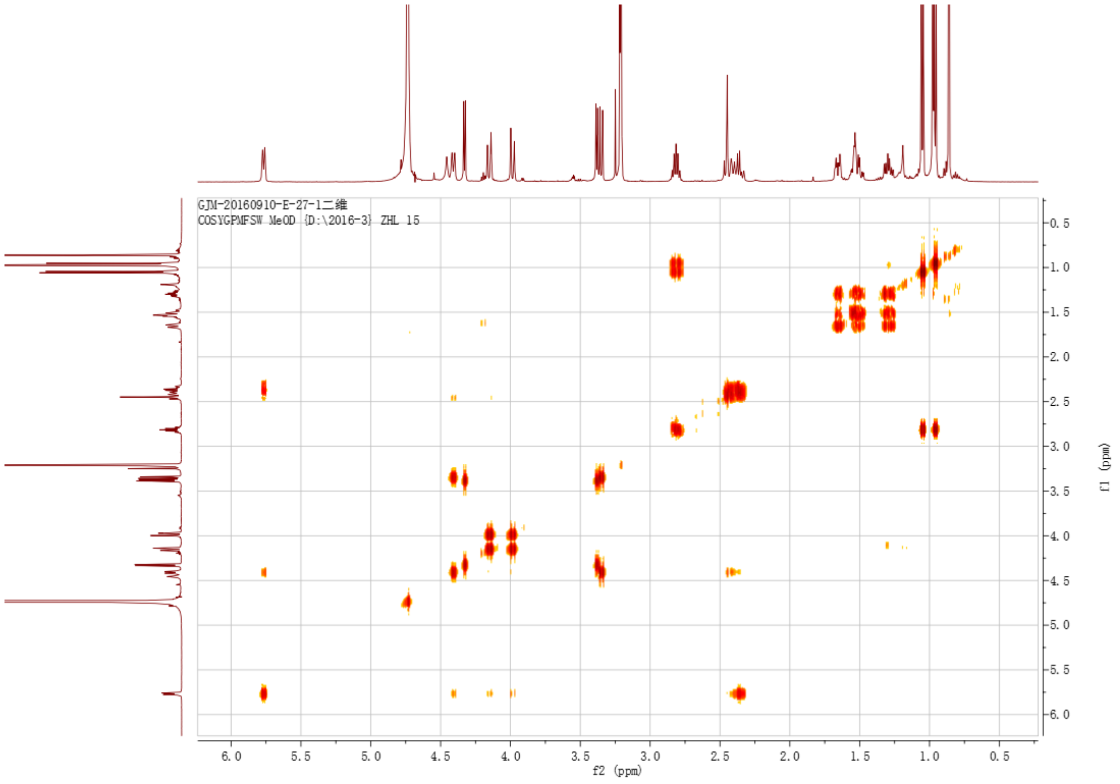

Figure S43. HMBC and NOESY spectra of compound **9** in CD_3OD.

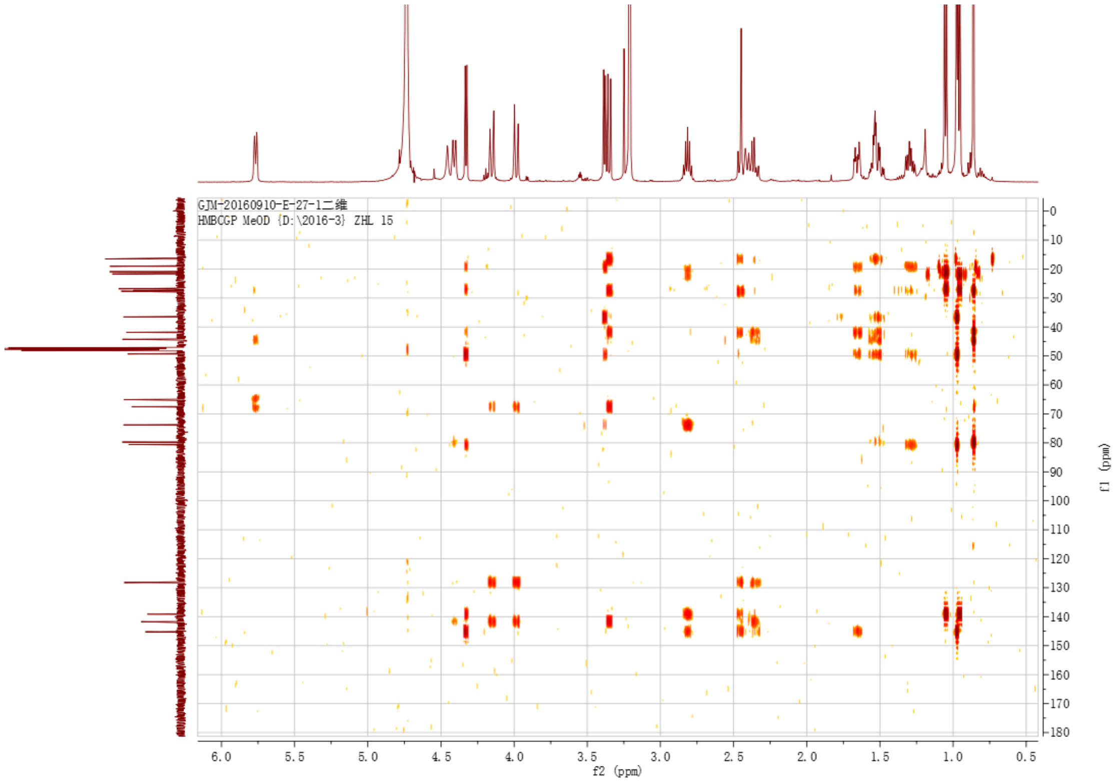
GJM-20160910-E-27-1二維
HMBCGP MeOD {D:\2016-3} ZHL 15
f2 (ppm)
f1 (ppm)

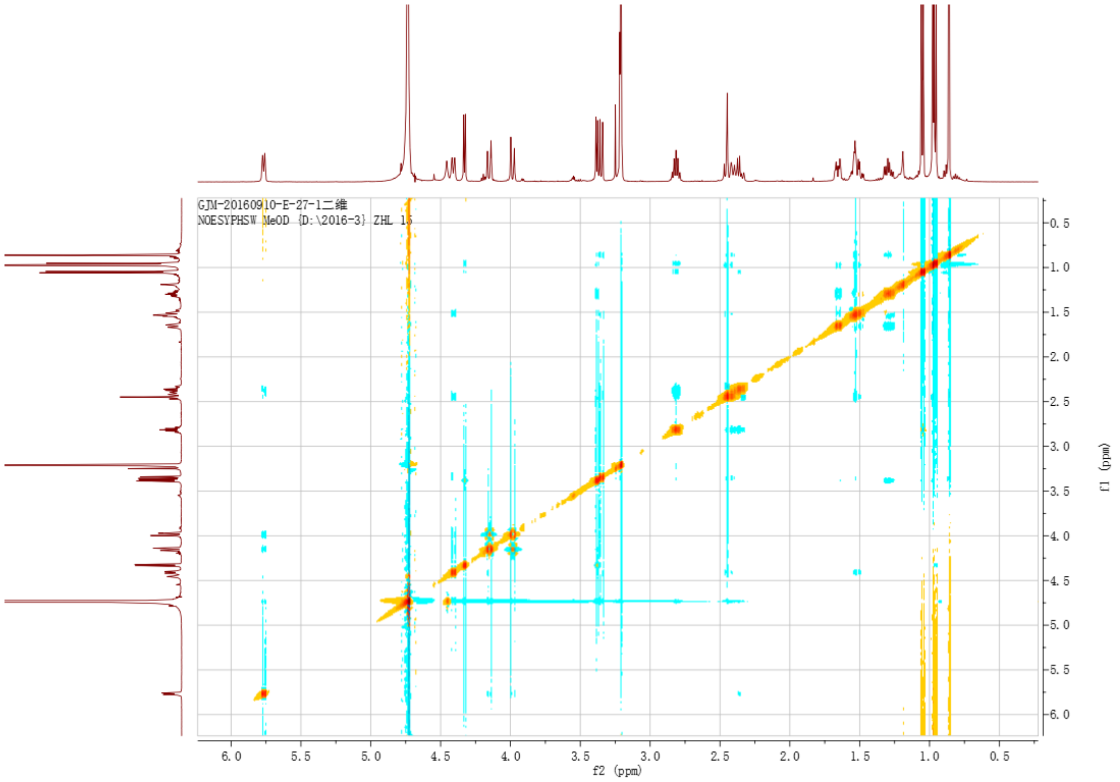
GJM-20160910-E-27-1二維
NOESYPHSW MeOD {D:\2016-3} ZHL 15
f2 (ppm)
f1 (ppm)

Figure S44. HRESIMS spectrum of compound **9**.

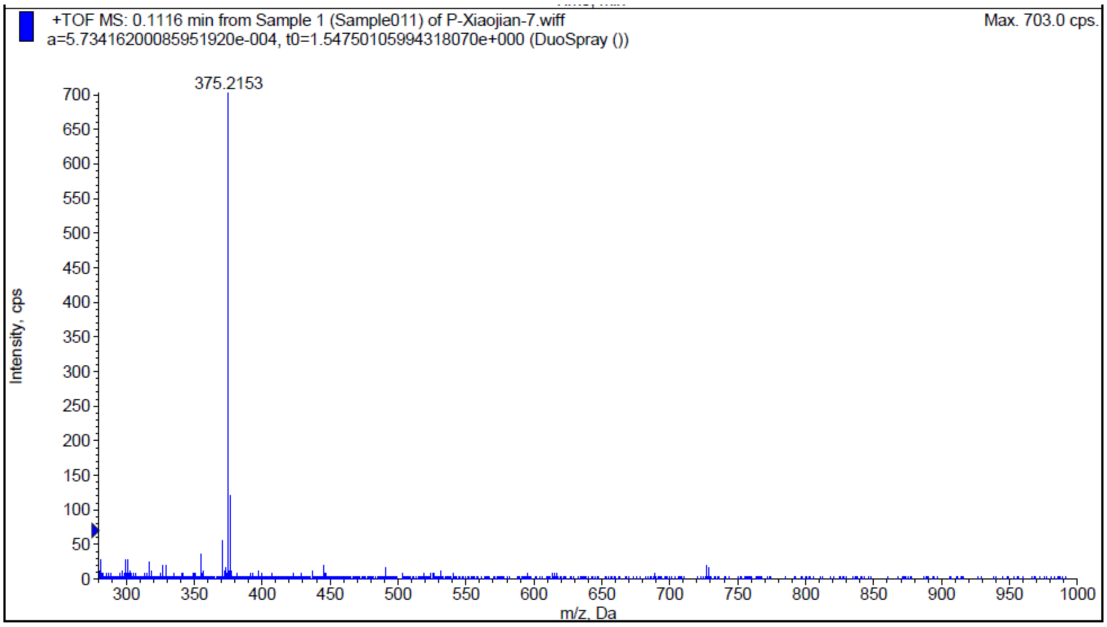

Figure S45. IR and UV spectra of compound **9**.

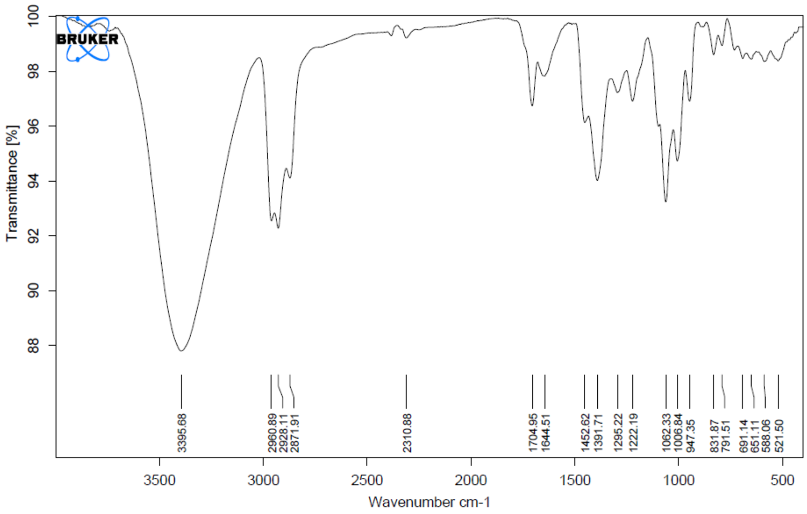

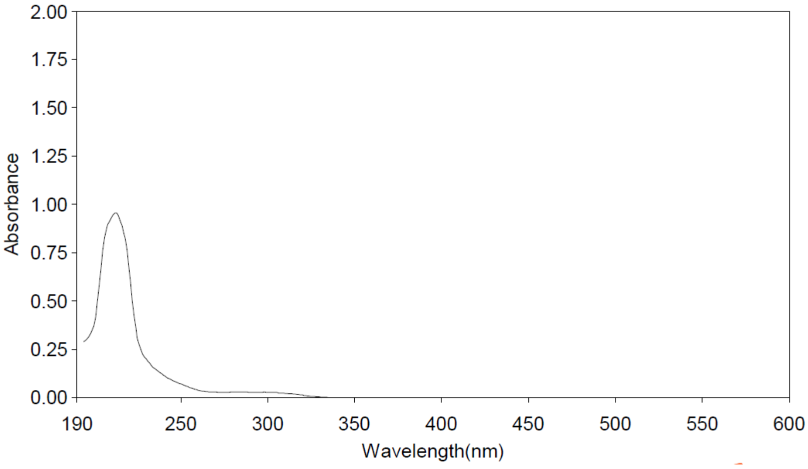

Figure S46. ^{1}H NMR and ^{13}C spectra of compound **10** in CD_3OD.

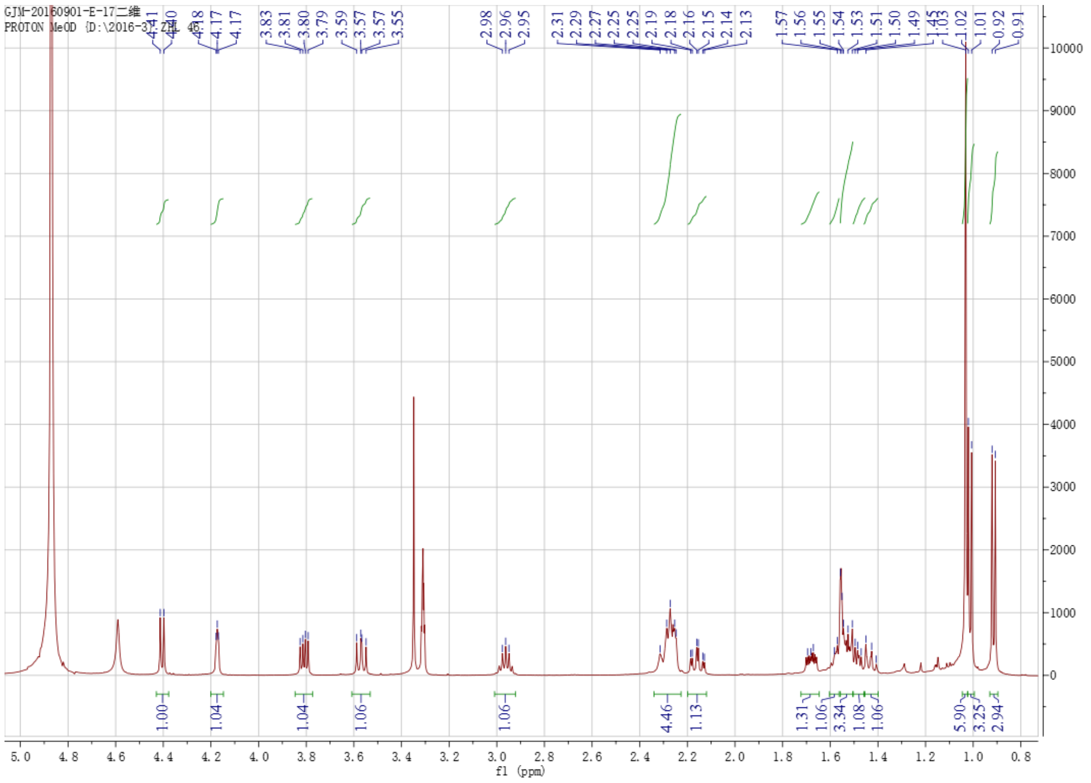

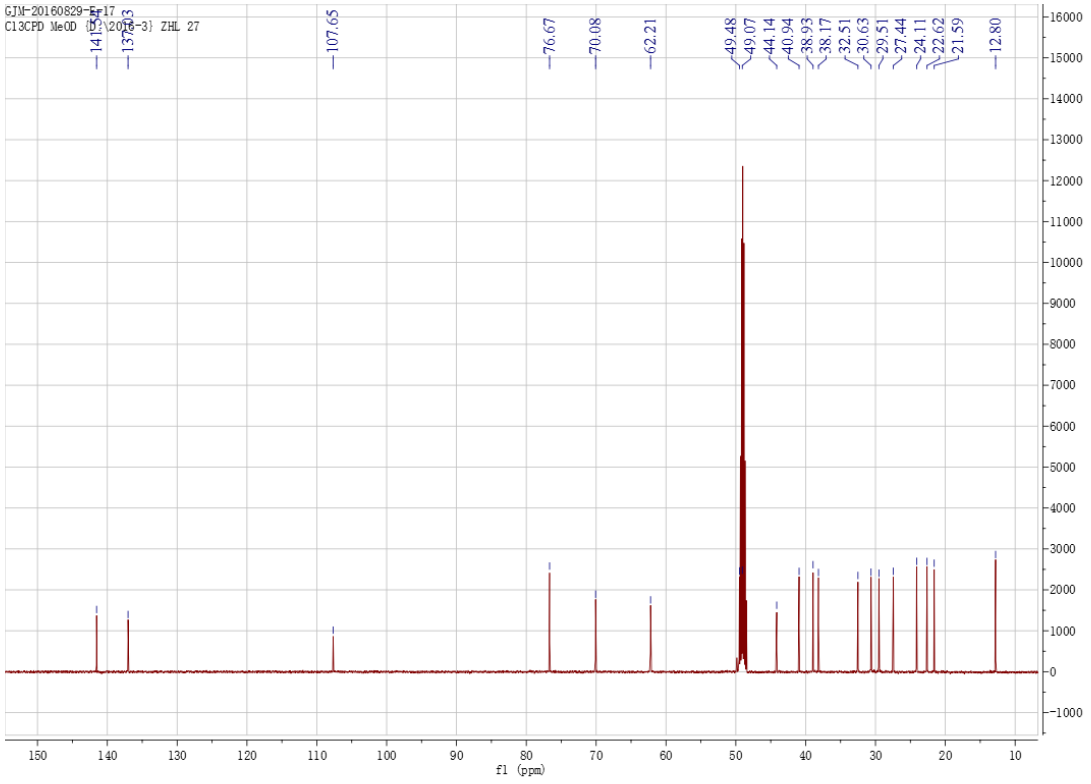

Figure S47. ESIMS spectrum of compound **10**.

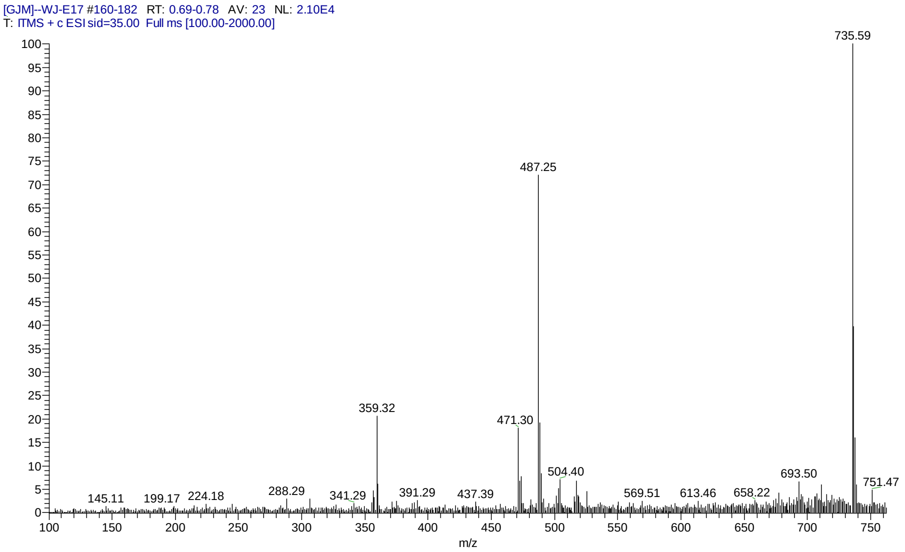

Figure S48. ^{1}H NMR and ^{13}C spectra of compound **11** in CD_3OD.

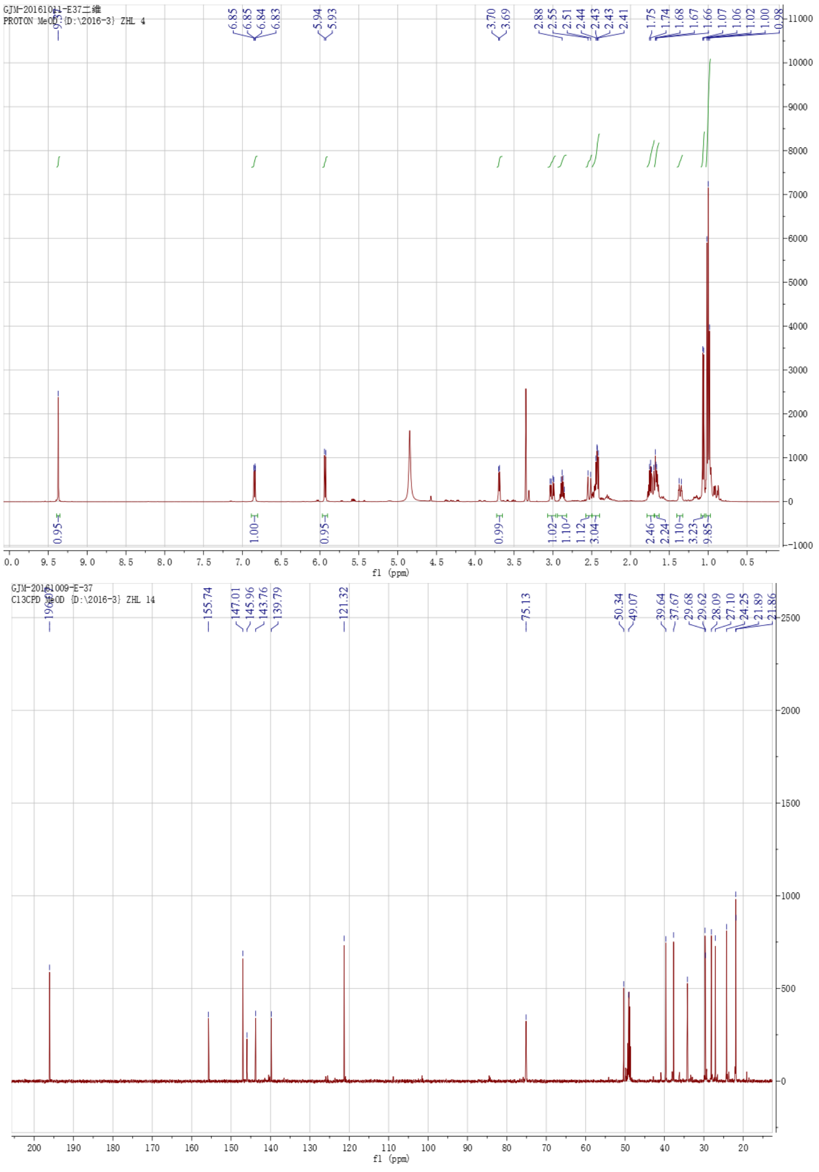

Figure S49. ESIMS spectrum of compound **11**.

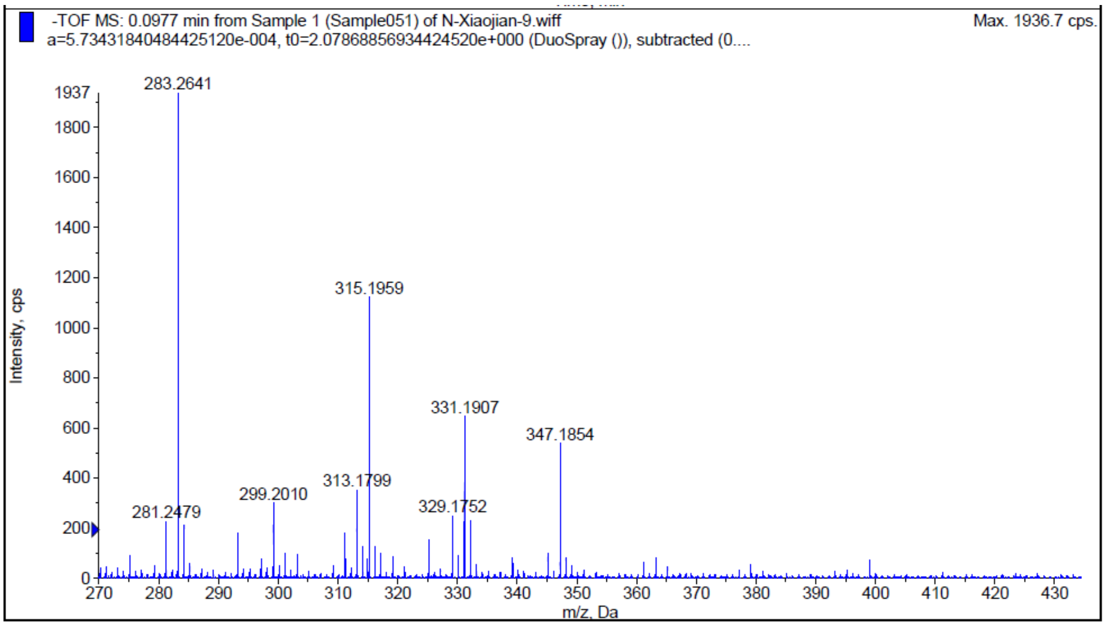

Figure S50. ^{1}H NMR and ^{13}C spectra of compound **6a** in CD_3CN.

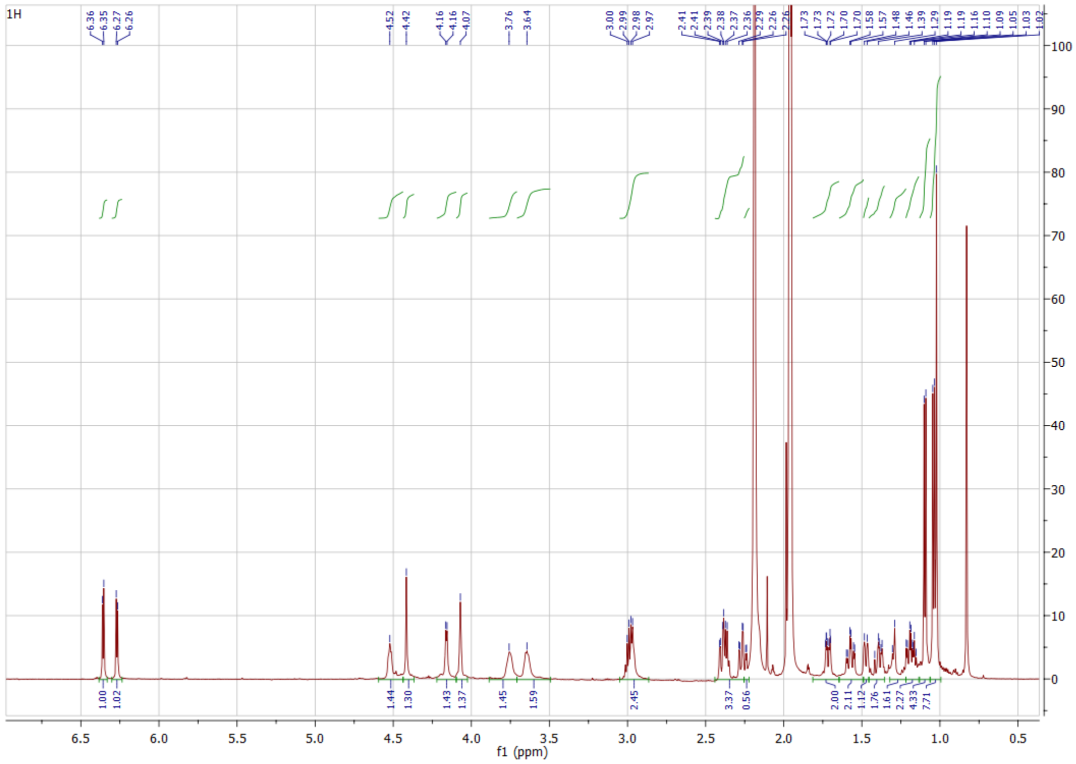

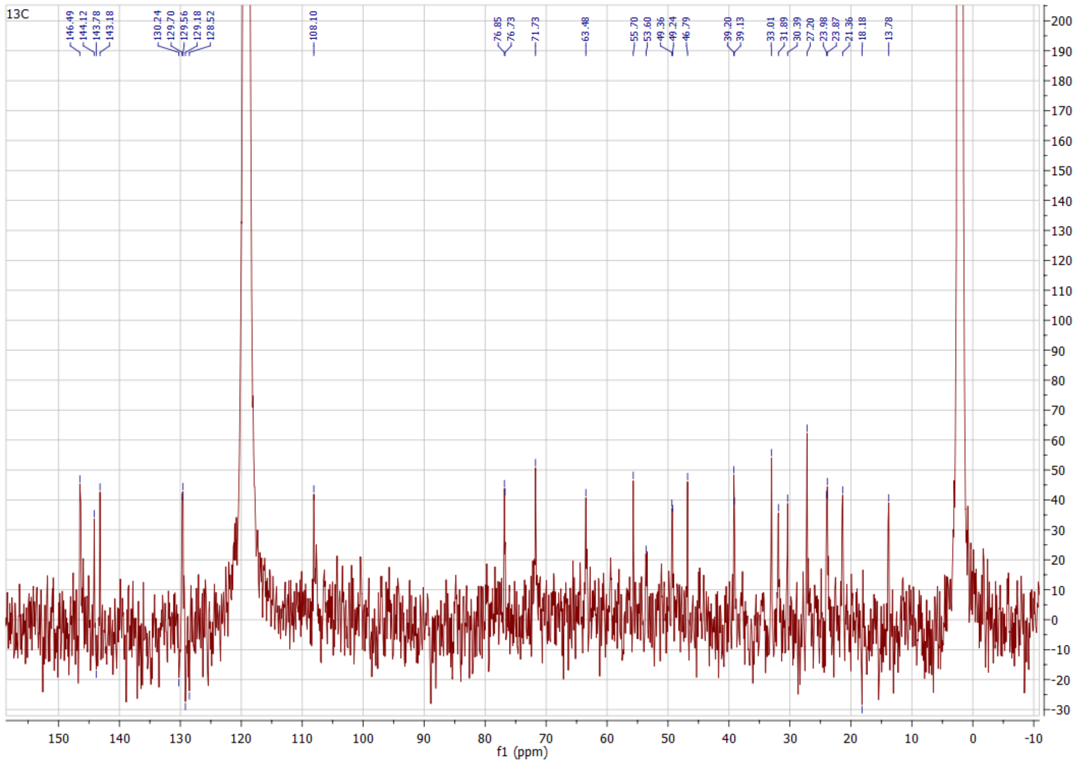

Figure S51. HSQC and ^{1}H-^{1}H TOCSY spectra of compound **6a** in CD_3CN

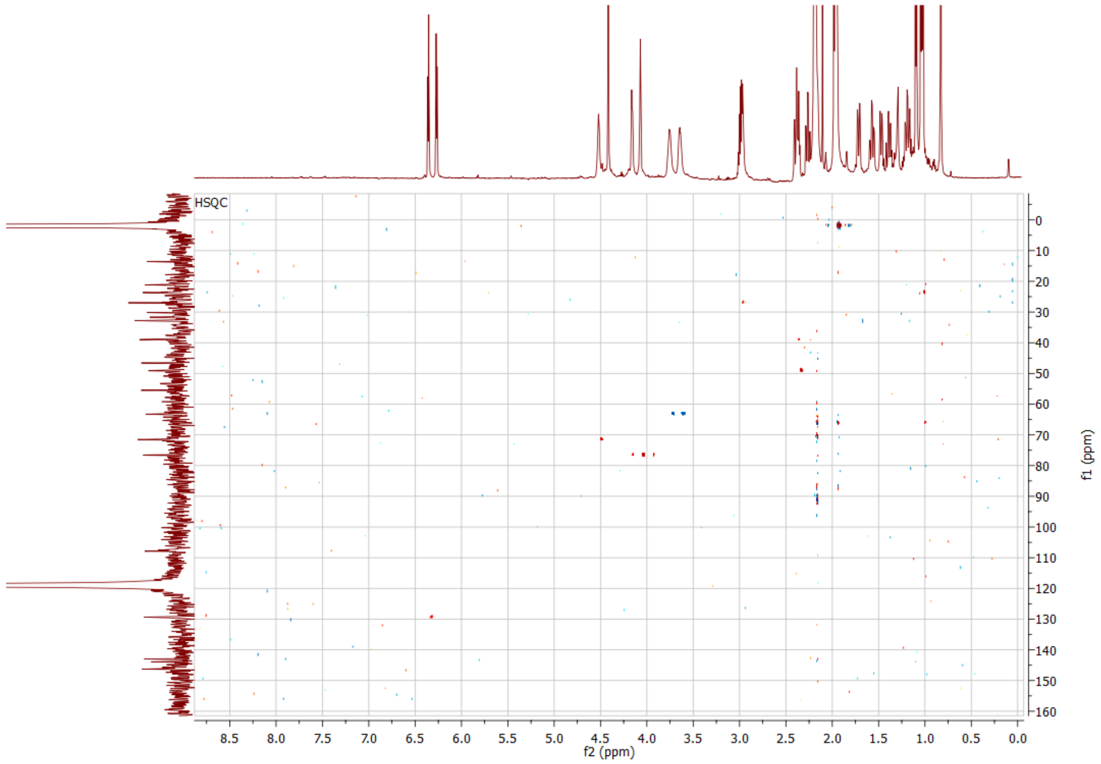

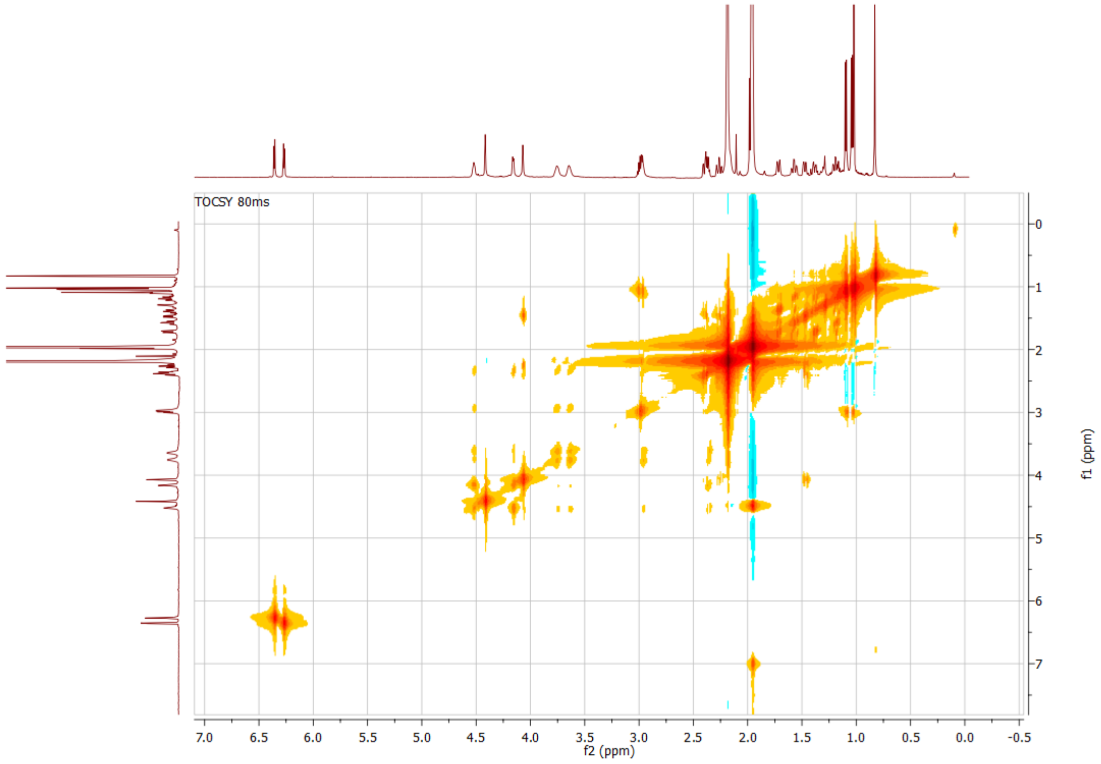

Figure S52. HMBC and ROESY (mixing 200 ms) spectra of compound **6a** in CD_3CN

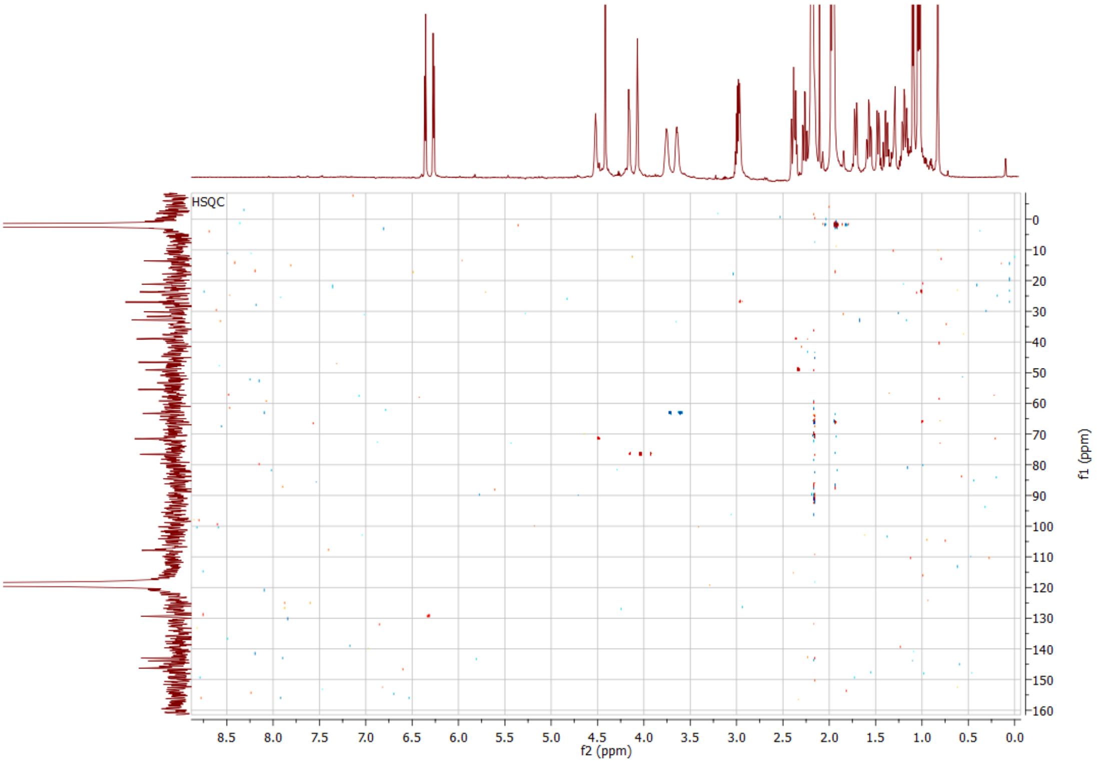

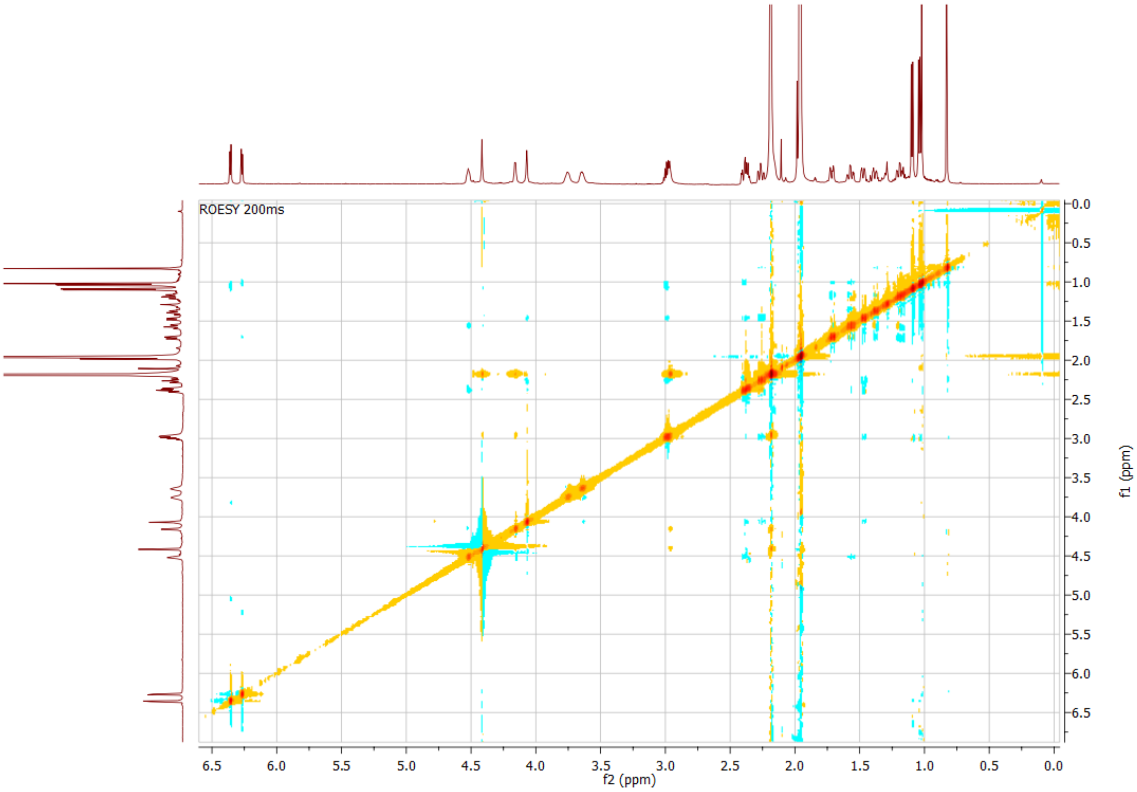

Figure S53. Cytotoxicity of the isolated compounds **1-10** to PC-12 cells

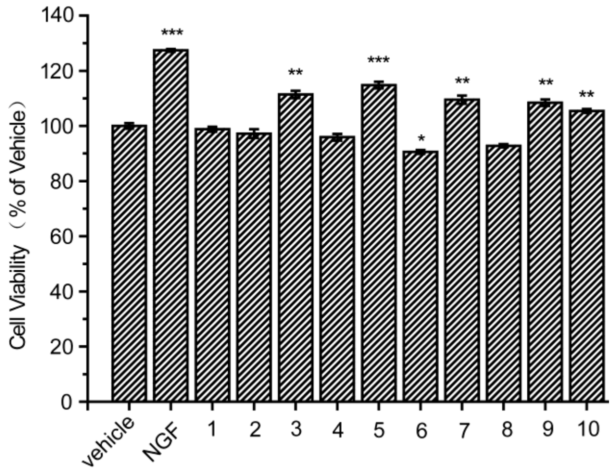

The test compounds at 10 μM for 48 h did not show any toxicity.

Figure S54. Effect of compound **1** on the NGF-promoted neurite outgrowth in PC-12 cells

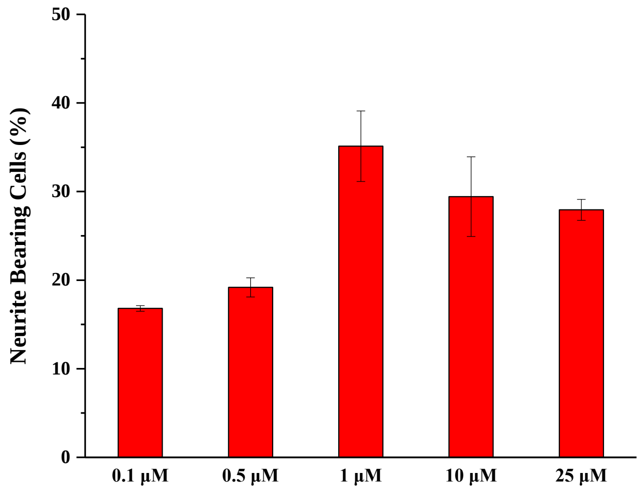

Table S1. ^{1}H and ^{13}C data of **9-11**, and cyathin I in CD_3OD.

No.	**9**		**10**		**11**		cyathin I[a]	
	δ_H	δ_C	δ_H	δ_C	δ_H	δ_C	δ_H	δ_C
1	3.49 d (6.0)	81.9	1.70 m<br>1.43 m	40.9	1.75 m<br>1.68 m	39.6	6.25 (d, 5.5)	145.8
2	4.43 d (6.0)	75.1	2.25 m	29.5	2.43 m	29.6	6.34 (d, 5.5)	128.9
3		140.5		141.5		145.9		143.0
4		146.6		137.0		143.7		143.1
5	2.55 m	45.6	2.31 m	38.9		155.7	2.45 (dd, 3.5, 13.0)	38.8
6		43.2		44.1		49.0		46.5
7	1.63 2H m	28.7	1.56 m<br>1.49 m	30.6	2.44 m<br>1.37 m	34.1	1.44 (m); 1.60 (td, 4.2, 13.6)	29.8
8	1.77 m<br>1.40 m	37.9	1.58 m	38.1	1.73 m<br>1.66 m	37.6	1.24(td, 4.6, 13.6); 1.72 (m)	32.4
9		50.7		49.0		50.3		55.1
10	2.47 2H m	29.0	2.16 td (12.7, 3.5)<br>1.53 m	32.5	5.94 d (8.1)	121.3	1.52 (m); 2.34 (m)	31.6
11	5.87 m	129.6	4.17 m	76.6	6.85 dd (8.1, 2.0)	147.0	4.24 (t, 3.0)	76.9
12		143.1	2.29 m	49.4		139.7	2.37 (m)	49.5
13	4.52 d (9.4)	68.9	4.41 d (7.7)	70.0	3.02 dd (17.6, 6.3)<br>2.55 d (17.6)	29.6	4.54 (d, 8.0)	70.3
14	3.46 d (9.4)	81.1		107.6	3.70 d (6.3)	75.1		107.8
15	4.26 d (12.7)<br>4.10 d (12.7)	66.4	3.81 dd (10.9, 5.9)<br>3.57 dd (10.9, 8.7)	62.2	9.37 s	196.0	3.61 (dd, 9.0, 10.5); 3.85 (dd, 5.5, 10.5)	62.2
16	0.96 s	17.8	1.03 s	12.8	1.00 s	27.1	0.89 (s)	12.9
17	1.08 s	20.4	1.03 s	24.1	1.02 s	24.2	1.04 (s)	20.6
18	2.91 m	28.2	2.96 m	27.4	2.88 m	28.0	3.00 (sept, 6.8)	26.9
19	1.16 d (6.9)	23.0	0.92 d (6.8)	21.5	1.07 d (6.9)	21.8	1.07 (d, 6.8)	23.0
20	1.07 d (6.9)	22.3	1.02 d (6.8)	22.6	0.98 d (6.9)	21.8	1.12 (d, 6.8)	23.1

[A] data from literature.

Table S2. Logarithms of Free Binding Energy of the NO Inhibitor to the Active Cavities of iNOS (PDBcode: 3E7G) and Targeting Residues with Hydrogen of the Binding Site.

	iNOS	
compound	log(FBE) kcal/mol	targeting residues
**11**	-9.22	ILE462
